# Draft genome of *Tanacetum cinerariifolium*, the natural source of mosquito coil

**DOI:** 10.1038/s41598-019-54815-6

**Published:** 2019-12-03

**Authors:** Takanori Yamashiro, Akira Shiraishi, Honoo Satake, Koji Nakayama

**Affiliations:** 1 Dainihon Jochugiku Co., Ltd., 1-1-11 Daikoku-cho, Toyonaka, Osaka, 561-0827 Japan; 2 Bioorganic Research Institute, Suntory Foundation for Life Sciences, Kyoto, 619-0284 Japan

**Keywords:** Plant evolution, Secondary metabolism

## Abstract

Pyrethrum (*Tanacetum cinerariifolium*), which is a perennial Asteraceae plant with white daisy-like flowers, is the original source of mosquito coils and is known for the biosynthesis of the pyrethrin class of natural insecticides. However, the molecular basis of the production of pyrethrins by *T. cinerariifolium* has yet to be fully elucidated. Here, we present the 7.1-Gb draft genome of *T. cinerariifolium*, consisting of 2,016,451 scaffolds and 60,080 genes predicted with high confidence. Notably, analyses of transposable elements (TEs) indicated that TEs occupy 33.84% of the genome sequence. Furthermore, TEs of the *sire* and *oryco* clades were found to be enriched in the *T. cinerariifolium*-specific evolutionary lineage, occupying a total of 13% of the genome sequence, a proportion approximately 8-fold higher than that in other plants. InterProScan analysis demonstrated that biodefense-related toxic proteins (e.g., ribosome inactivating proteins), signal transduction-related proteins (e.g., histidine kinases), and metabolic enzymes (e.g., lipoxygenases, acyl-CoA dehydrogenases/oxygenases, and P450s) are also highly enriched in the *T. cinerariifolium* genome. Molecular phylogenetic analysis detected a variety of enzymes with genus-specific multiplication, including both common enzymes and others that appear to be specific to pyrethrin biosynthesis. Together, these data identify possible novel components of the pyrethrin biosynthesis pathway and provide new insights into the unique genomic features of *T. cinerariifolium*.

## Introduction

**Figure 1:**
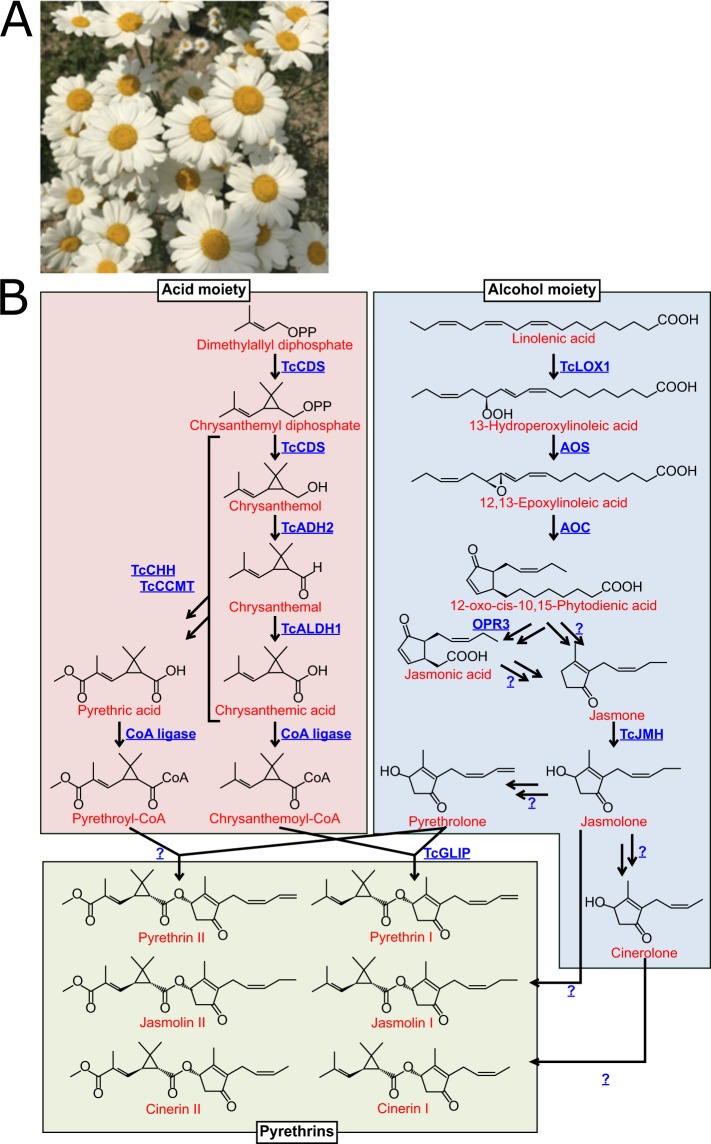
Flower of *T. cinerariifolium* (**A**) and biosynthetic pathway of pyrethrins (**B**). Proposed biosynthetic pathway of pyrethrins. Chrysanthemic acid and pyrethric acid as acid moieties are synthesized from dimethylallyl diphosphates by *T. cinerariifolium* chrysanthemyl diphosphate synthase (TcCDS), *T. cinerariifolium* alcohol dehydrogenase 2 (TcADH2), *T. cinerariifolium* aldehyde dehydrogenase 1 (TcALDH1), *T. cinerariifolium* chrysanthemol 10-hydroxylase (TcCHH), and *T. cinerariifolium* 10-carboxychrysanthemic acid 10-methyltransferase (TcCCMT). Pyrethrolone, jasmolone, and cinerolone as alcohol moieties are synthesized from linolenic acid by enzymes related to oxylipin pathway, including *T. cinerariifolium* jasmone hydroxylase (TcJMH), and unknown enzyme(s). Finally, chrysanthemoyl-CoA (the CoA-activated version of chrysanthemic acid) and pyrethrolone are esterified by *T. cinerariifolium* GDSL (Gly-Asp-Ser-Leu motif) lipase (TcGLIP) to produce pyrethrin I. Compounds and enzymes are indicated by red and blue text, respectively. Unidentified enzymes are indicated as question marks.

Insect-borne infection is one of the most familiar and prevalent diseases worldwide. To date, a wide variety of insecticides and insect repellents have been developed to prevent insect-borne infection. Mosquito coils, which were first commercialized by Dainihon Jochugiku Co., Ltd., in 1902, are the origin of potent insecticides and insect repellents that are harmless to humans. The natural source of the original mosquito coil was the flower head of pyrethrum (*Tanacetum cinerariifolium*), a perennial Asteraceae plant with a white daisy-like flower (Fig. 1A). The major insecticidal compounds are *T. cinerariifolium*-specific specialized metabolites designated pyrethrins. These phytochemicals are biosynthesized by esterification of acid moieties (chrysanthemic acid and pyrethric acid) and alcohol moieties (pyrethrolone, jasmolone, and cinerolone)^1^, yielding a total of six compounds, pyrethrin I and II, jasmolin I and II, and cinerin I and II, by combination of the acid and alcohol moieties^1^ in *T. cinerariifolium*. Pyrethrins exhibit highly selective neurotoxicity to insects and are degraded rapidly in the presence of sunlight and oxygen^2,3^. Characterization of pyrethrins led to the development of pyrethroids, structurally related compounds that are easily synthesized^4^. Both pyrethrins and pyrethroids have been extensively employed as common insecticides and insect repellents that have provided protection against insect-borne diseases such as malaria and dengue fever in many countries^5,6^.

To date, some pyrethrin biosynthesis-related enzymes have been identified^1,7–11^ (Fig. 1B). Regarding the generation of alcohol moieties of pyrethrins, linolenic acid is converted to 13-hydroperoxylinolenic acid by *T. cinerariifolium* 13-lipoxygenase (TcLOX1) and subsequently converted to jasmone by allene oxide cyclase (AOC), allene oxide synthase (AOS), 3-oxo-2-(2-pentenyl)-cyclopentane-1-octanoic acid reductase 3 (OPR3), and additional unknown enzymes. Jasmone is converted to jasmolone by *T. cinerariifolium* jasmone hydroxylase (TcJMH). Although pyrethrolone and cinerolone are thought to be derived from jasmolone, the mechanisms and enzymes responsible for this conversion have yet to be elucidated. Regarding the synthesis of the acid moieties of pyrethrins, two molecules of dimethylallyl diphosphate are condensed to chrysanthemyl diphosphate by *T. cinerariifolium* chrysanthemyl diphosphate synthase (TcCDS); the product subsequently is oxidized to chrysanthemic acid by *T. cinerariifolium* alcohol dehydrogenase 2 (TcADH2) and *T. cinerariifolium* aldehyde dehydrogenase 1 (TcALDH1). The other acid moiety, pyrethric acid, is generated by *T. cinerariifolium* chrysanthemol 10-hydroxylase (TcCHH) and *T. cinerariifolium* 10-carboxychrysanthemic acid 10-methyltransferase (TcCCMT). Finally, CoA-activated chrysanthemic acid (chrysanthemoyl-CoA) and pyrethrolone are esterified to pyrethrin I by *T. cinerariifolium* GDSL (Gly-Asp-Ser-Leu motif) lipase (TcGLIP). While TcGLIP has been isolated and identified as the enzyme catalyzing the esterification of pyrethrin I, the enzymes that catalyze the synthesis of pyrethrin II, jasmolin I, jasmolin II, cinerin I, and cinerin II have yet to be identified. In this context, elucidation of *T. cinerariifolium* genome sequence is expected to provide crucial clues for clarification of the whole biosynthetic pathway of the pyrethrins and of the evolutionary processes. The genome sequence also is expected to facilitate the development of molecular tools for the generation of transgenic *T. cinerariifolium* and other systems for efficient and sustainable production of natural pyrethrins that can be difficult to chemically synthesize.

Genomes of various members of the Asteraceae family, including *Chrysanthemum seticuspe*^12^, *Artemisia annua*^13^ and *Helianthus annuus*^14^, have been reported over the past few years, but no pyrethrin-producing member of the Asteraceae family have been sequenced^15^. The genome of *T. cinerariifolium* was estimated to be approximately 7.1 Gb by a flow cytometric method^16^, indicating a genome that is more than twice the sizes of other Asteraceae genomes (e.g., 2.72 Gb for *C. seticuspe*, 1.74 Gb for *A. annua* and 3.6 Gb for *H. annuus*). Assembling such large genomes still remains challenging due to the presence of highly repetitive sequences, multiplied paralogous genes, and heterozygosity^17^. Here, we report a draft genome of *T. cinerariifolium* derived by massively parallel sequencing using Hiseq4000 and X. The repetitive sequence content and multiplied pyrethrin-biosynthetic genes included in the assembled genome sequence also were analyzed.

## Result

### Sequence assembly and annotation of *T. cinerariifolium* genome

**Figure 2:**
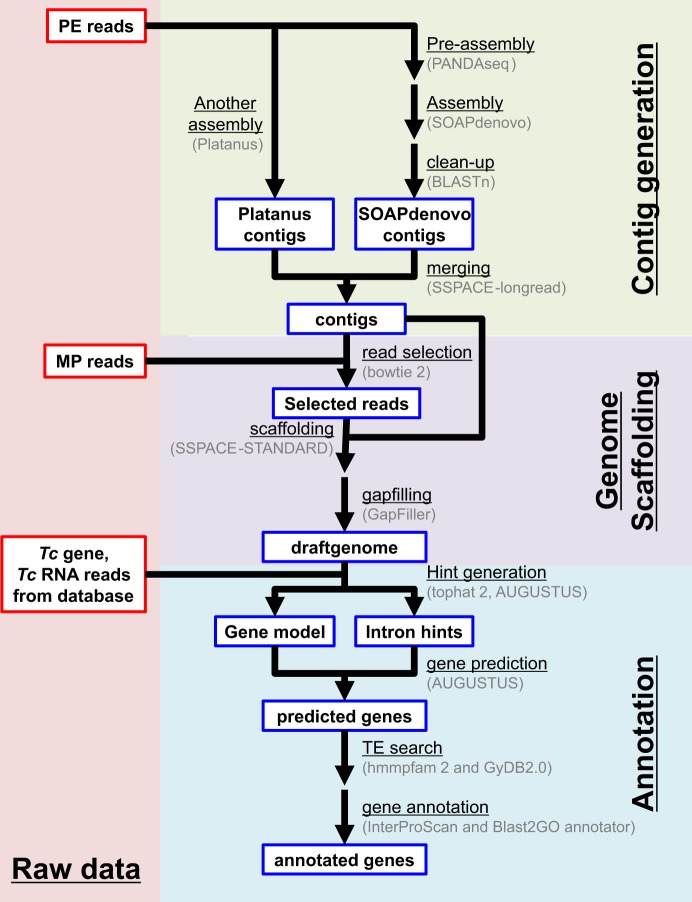
Flowchart of genome assembly and gene prediction. The PE reads were subjected to contig assembly by a four-step process, including “pre-assembly” using PANDAseq, “contig assembly” using SOAPdenovo and Platanus, “clean-up” using BLASTN, and “merging with other assembly result” using SSPACE-longread. Then, the MP reads were subjected to scaffolding by a three-step process, including “read selection” using bowtie2, “scaffolding” using SSPACE-STANDARD, and “gapfilling” using GapFiller. These processes yielded the complete draft genome. The coding sequences in the draft genome then were annotated by a three-step process, including “gene prediction” using Augustus, “transposable elements (TEs) detection” using hmmpfam, and “protein superfamilies prediction” using InterProScan.

Paired-end (PE) libraries and mate-pair (MP) libraries (with 3-, 5- and 8-kb insert sizes; MP-3 kb, MP-5 kb, and MP-8 kb, respectively) of the *T. cinerariifolium* genome were generated and then sequenced using Hiseq X and Hiseq4000 instruments. The total bases of the sequence reads for PE, MP-3 kb, MP-5 kb, and MP-8 kb amounted to 1,497 Gb, 135 Gb, 166 Gb, and 95 Gb, respectively (Table 1). Subsequently, the output reads were subjected to contig assembly and scaffolding steps to estimate the genome sequence (Fig. 2). The 3,892,368 contigs with total length of 7.7 Gb (Table 2) were constructed by assembling PE reads using SOAPdenovo^18^ and Platanus^19^ and merging the contigs using SSPACE-Longread^20^. The assembled contigs then were scaffolded with PE and MP reads using SSPACE^21^, which concatenates contig sequences with pair information of the reads, and Gapfiller^22^, which fills the inter-contig unknown bases (‘N’s) in scaffolds with ‘A/T/G/C’ (Fig. 2). The total length of the resultant scaffolds was 7.1 Gb, which corresponded with the flow cytometry-estimated genome size for *T. cinerariifolium*^16^. The N50 value of the scaffolds was 14 Kb, and the maximum length of the scaffolds was 1.2 Mb (Table 2). Subsequently, the draft genome was subjected to Augustus^23^ with ‘intron hints’, which were generated with previous *T. cinerariifolium* transcriptomes^24,25^, resulting in the prediction of 935,992 putative genes.

**Table 1:** Statistics of sequence reads.

Library	Insert size (bp)	Read length (bases)	Number of reads	Total read length (bases)	Expected depth (=Total read length/7.1 Gb)
PE	350	151	4,956,379,975	1,496,826,752,450	210.82
MP	3,000	101	665,902,933	134,512,392,466	18.95
MP	5,000	101	822,858,639	166,217,445,078	23.41
MP	8,000	101	471,281,672	95,198,897,744	13.41

*PE, paired-end; MP, mate-pair.

**Table 2:** Statistics of genome assembly.

	Contigs	Scaffolds
Total number of sequence fragments	3,892,368	2,016,451
Total length (bp)	7,710,571,398	7,084,225,540
N50 (bp)	8,078	13,813
Length of longest contig (bp)	1,189,490	1,185,691
GC content (%)	35.1	35.1

To evaluate the completeness of the genome, the draft genome sequences were subjected to analysis with BUSCO^26^, which counts complete (C), fragmented (F), and missing (M) conserved genes in genome sequences. The analysis with 1440 conserved core plant genes showed that 91.8% of conserved genes (82.3% as complete and 9.5% as fragmented) were present in the *T. cinerariifolium* genome assembly (Table 3). Since these scores indicated quality as high as those of other plant genomes (with reported scores of 83.8% in *Cuscuta campestris*^27^, 77.5% in *Ruellia speciosa*^28^, 86.9% in *Erigeron breviscapus*^29^, and 89.0% in *Secale cereal*^30^), we used the *T. cinerariifolium* draft genome for subsequent analysis.

**Table 3:** Annotation statistics for draft genome.

Number of predicted genes	935,992	
BUSCO v3	C:82.3% (Single: 72.8%, Duplicated:9.5%) F:9.5% M:8.2%	
Number of predicted TEs	525,098	
Region of TEs	Total	2,397 Mbp (33.84%)
	Ty1/Copia Ty3/Gypsy Retroviridae Caulimoviridae Bel/Pao others	1,135 Mbp (16.02%) 907 Mbp (12.81%) 222 Mbp (3.13%) 88 Mbp (1.24%) 18 Mbp (0.25%) 28 Mbp (0.39%)
Number of predicted genes encoding products with known protein signatures	60,080	

^*^C: percentage of full-length conserved genes in BUSCO notation; F: percentage of fragmented conserved genes in BUSCO notation; M: percentage of missing genes in BUSCO notation; TE: transposable element.

To annotate the predicted genes, transposable elements (TEs) were first annotated using hmmpfam^31^ against the Gypsy database (GyDB)^32^, revealing the presence of 525,098 TEs occupying 2,397 Mbp (33.84%) of the total genome. Further classification of TEs showed that 1,135 Mbp (16.02%), 907 Mbp (12.81%), 222 Mbp (3.13%), 88 Mbp (1.24%), 18 Mbp (0.25%), and 28 Mbp (0.39%) were occupied by Ty1/Copia, Ty3/Gypsy, Retroviridae, Caulimoviridae, Bel/Pao, and other TEs. Among Ty1/Copia and Ty3/Gypsy, the *sire* (Ty1/Copia, 11.16%), *athila* (Ty3/Gypsy, 5.76%), *del* (Ty3/Gypsy, 4.05%), and *oryco* (Ty1/Copia, 2.14%) clades were abundant (Table 3). The remaining 410,894 non-TE genes were subjected to InterProScan 5.6–48.0^33^, revealing 60,080 putative genes exhibiting known protein signatures. Thus, a high-quality 7.1-Gb *T. cinerariifolium* draft genome was assembled and shown to include a total of 525,098 TEs and 60,080 plausible genes based on 1,497 Gb of PE reads and 396 Gb of MP reads.

### Inter-genus comparative analysis of TE classification

**Figure 3:**
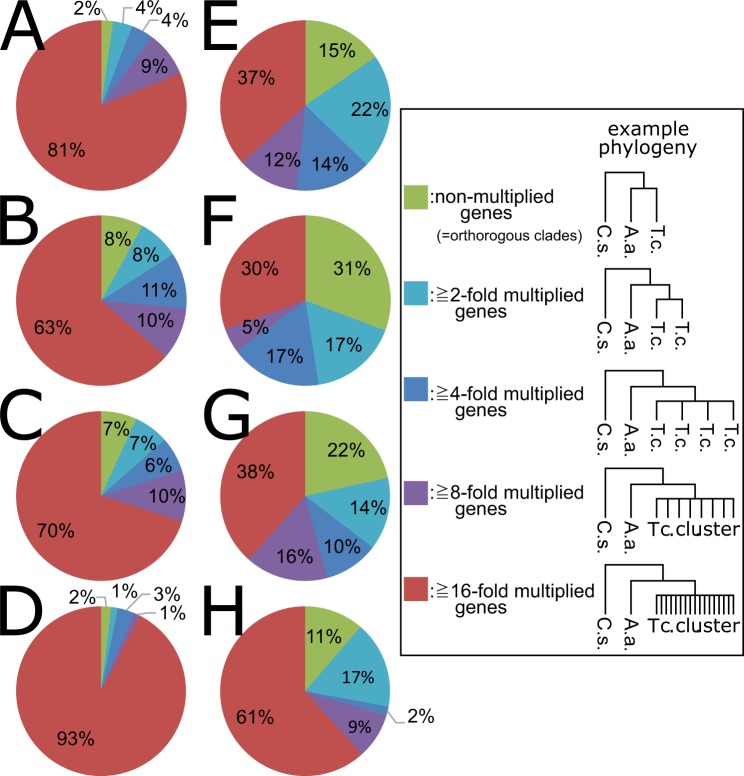
Multiplication analysis of *sire*-clade (**A–D**) and *oryco*-clade (**E,F**) transposable elements (TEs). Based on molecular phylogenetic trees for *sire* TEs (Supplemental Fig. 1) and *oryco* TEs (Supplemental Fig. 2), the number of co-clustered genes in single-genus clusters, which reflects the number of genus-specific duplication events, were counted for each genus. The counted number of co-clustered genes for *T. cinerariifolium* (**A,E**), *C. seticuspe* (**B,F**), *A. annua* (**C,G**), and *H. annuus* (**D,H**) are shown in the form of pie charts.

We compared *T. cinerariifolium* TE clades with those of other species by predicting the TEs in the genomes of *Arabidopsis thaliana*, *Nicotiana tabacum*, and *Oryza sativa*, revealing the presence in *T. cinerariifolium* of two TE clades that were enriched by more than 2-fold compared to these other plant genomes (Table 4). In *T. cinerariifolium*, members of the *sire-*clade TEs were found to be present at proportions 14.3-, 8.5-, and 8.1-fold higher than in *A. thaliana*, *N. tabacum*, and *O. sativa*, respectively (Table 4). Similarly, members of the *oryco*-clade TEs were found to be present at proportions 2.2-, 4.6-, and 2.6-fold higher, respectively. These results indicated that *sire*- and *oryco*-clade TEs constitute a considerably larger proportion of the *T. cinerariifolium* genome than is observed in these other plants. Furthermore, a comparison of the proportions of these TEs in *T. cinerariifolium* to those of these TEs in other Asteraceae (*C. seticuspe*, *A. annua* and *H. annuus*) (Table 5) demonstrated that the *sire*- and *oryco*-clade TEs are remarkably enriched in all of these Asteraceae genera. To assess whether *sire*- and *oryco*-clade TEs had multiplied in a common ancestor of the Asteraceae or independently in individual genera, we analyzed molecular phylogenetic trees of the reverse transcriptase (RT) domains encoded by the *sire* and *oryco* sequences (Supplemental Figs. 1 and 2); specifically, we evaluated the number of co-clustered genes in single-genus clusters, which should reflect the number of genus-specific duplication events (Fig. 3). If these TEs had multiplied in a common ancestor, most TEs would be expected to be positioned in orthologous clusters with bootstrap values greater than 50% (see Fig. 3, example of non-multiplied clusters). Otherwise, most TEs would be expected to be positioned in multiplied clusters (Fig. 3). In fact, 98%, 92%, 93%, and 98% of *sire* TEs were separately multiplied in *T. cinerariifolium* (Fig. 3A), *C. seticuspe* (Fig. 3B), *A. annua* (Fig. 3C), and *H. annuus* (Fig. 3D), respectively, indicating that most of the *sire* TEs had multiplied in the individual genera. Likewise, 85%, 69%, 78%, and 89% of *oryco* TEs were multiplied in the respective organisms (Fig. 3E–H), indicating that most of the *oryco* TEs also had multiplied in the individual genera. Moreover, 90% of *sire* TEs (Fig. 3A) and 49% of *oryco* TEs (Fig. 3E) clustered with eight or more multiplied genes. Collectively, these results suggest that *sire* and *oryco* TEs were multiplied in the individual lineages of the respective Asteraceae genera; in the case of *T. cinerariifolium*, TEs of these clades occupy approximately 13% of the genome.

**Table 4:** Abundance score of TEs in other plants of the Asteraceae family.

Family	Clade	Tc	Nt	Os	At
Ty1/Copia	*sire*	11.16%	1.31%	1.38%	0.78%
		(790,364,218)	(47,853,545)	(5,299,804)	(936,463)
Ty3/Gypsy	*athila*	5.76%	4.30%	1.26%	3.21%
		(408,032,509)	(156,681,791)	(4,813,373)	(3,843,212)
Ty3/Gypsy	*del*	4.05%	17.59%	3.08%	0.96%
		(286,557,581)	(640,748,439)	(11,774,484)	(1,146,571)
Ty1/Copia	*oryco*	2.14%	0.47%	0.83%	0.96%
		(151,866,453)	(17,163,885)	(3,191,553)	(1,150,943)
Retro-viridae	*lentiviridae*	1.67%	0.53%	0.84%	1.02%
		(118,004,479)	(19,437,617)	(3,226,018)	(1,219,271)

^*^Parenthesized numbers indicate the size (bp) of the region categorized in each clade. Tc, *Tanacetum cinerariifolium*; Nt, *Nicotiana tabacum*; Os, *Oryza sativa*; At, *Arabidopsis thaliana*; TE: transposable element.

**Table 5:** Abundance score of TEs in Asteraceae plants.

Family	Clade	Tc	Cs	Aa	Ha
Ty1/Copia	*sire*	11.16%	9.87%	6.34%	2.30%
		(790,364,218)	(268,554,348)	(113,663,806)	(83,863,222)
Ty3/Gypsy	*athila*	5.76%	3.36%	5.71%	1.21%
		(408,032,509)	(91,399,675)	(102,303,891)	(44,010,125)
Ty3/Gypsy	*del*	4.05%	1.55%	1.91%	8.80%
		(286,557,581)	(42,223,327)	(34,214,927)	(320,646,159)
Ty1/Copia	*oryco*	2.14%	1.58%	1.33%	1.04%
		(151,866,453)	(42,884,278)	(23,928,063)	(37,770,638)
Retro-viridae	*lentiviridae*	1.67%	1.56%	1.04%	2.04%
		(118,004,479)	(42,471,725)	(18,691,180)	(74,263,619)

^*^Parenthesized numbers indicate the size (bp) of the region categorized in each clade. Tc, *Tanacetum cinerariifolium*; Cs, *Chrysanthemum seticuspe*; Aa, *Artemisia annua*; Ha, *Helianthus annuus*; TE: transposable element.

**Figure 4:**
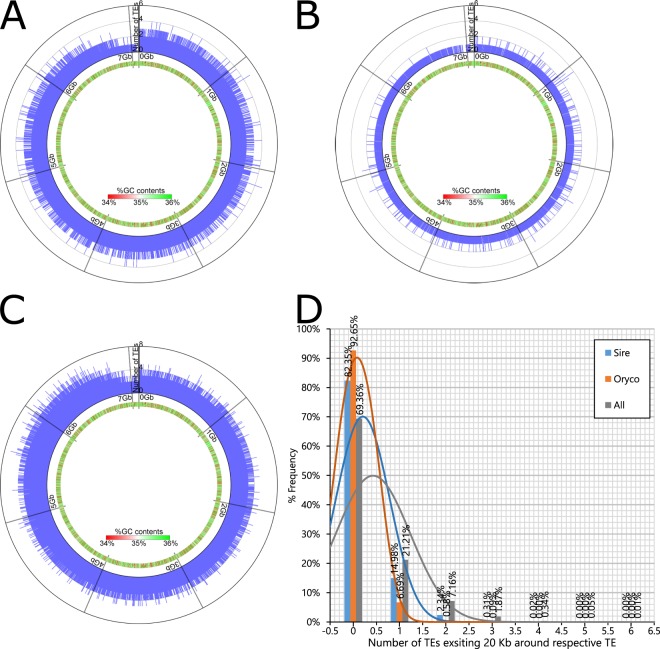
The distribution of transposable elements (TEs) in the *T. cinerariifolium* genome. The distributions of TEs for *sire* (**A**), *oryco* (**B**) and all clade (**C**) were shown with the circles. The number of TEs coded in each scaffold regions within each 20 Kb region was plotted on the outer circle and %GC content in each 20 Kb was represented with heatmap on the inner circle. (**D**) The number of *sire*, *oryco* and all clade existing 20 Kb around respective TE was shown with histogram.

We also analyzed the distribution of TEs in genome scaffolds by counting the number of *sire* and *oryco* genes encoded in each 20-Kb region. As shown in Fig. 4, uniform distributions of *sire* and *oryco* genes were observed using frequency plots for *sire* (Fig. 4A) and *oryco* (Fig. 4B) genes in each 20-Kb interval. Furthermore, no local distribution was detected by frequency plots for any of the TE clades (Fig. 4C). We next analyzed the number of TEs present within 20 Kb of another member of the respective TE clade. As shown in Fig. 4D, 82.35% and 92.65% of *sire* and *oryco* genes (respectively) were separated by more than 20 Kb from other TEs of the respective clade. Together, these data indicated that the TEs that multiplied in *T. cinerariifolium*, are scattered throughout the entire genome.

### Functional annotation of the *T. cinerariifolium* genes and inter-genus comparative analysis

Next, we investigated multiplication ratios of protein superfamilies in *T. cinerariifolium* compared with those in other species. This analysis was performed using InterProScan^33^. Specifically, the protein data sets of *C. seticuspe, A. annua, H. annuus, N. tabacum, O. sativa*, and *A. thaliana* were subjected to analysis using InterProScan and the multiplication odds scores were calculated for each combination of superfamily and species (Table 6). A positive value of the multiplication odds score indicates that a genus possesses a higher number of multiplied genes in a given superfamily than do other genera. As shown in Table 6, the highest multiplication odds scores for *T. cinerariifolium* were observed for the biodefense-, signaling-, and metabolism-related superfamilies.

**Table 6:** Superfamilies with highest multiplication odds scores in *T. cinerariifolium*.

Category	InterPro ID	Superfamily name	Tc	Cs	Aa	Ha	Nt	Os	At
Biodefense	IPR036041	Ribosome-inactivating protein	2.05 (98)	−1.05 (7)	−0.24 (16)	−2.31 (0)	−2.31 (0)	−0.17 (17)	−2.31 (0)
Biodefense	IPR035992	Ricin B-like lectins	1.57 (69)	−0.18 (17)	0.11 (22)	−1.06 (7)	−0.64 (11)	−0.94 (8)	−1.32 (5)
Signaling	IPR036097	Signal transduction histidine kinase, dimerization/phosphoacceptor domain	1.39 (101)	−0.64 (21)	−0.39 (26)	−0.3 (28)	0.33 (46)	−1.76 (7)	−0.76 (19)
Signaling	IPR024792	Rho GDP-dissociation inhibitor domain	1.32 (34)	−0.06 (10)	−0.51 (6)	−0.27 (8)	−0.06 (10)	−0.97 (3)	−0.97 (3)
Signaling	IPR035983	HECT, E3 ligase catalytic domain	1.14 (72)	0.4 (41)	−0.22 (25)	−0.6 (18)	0.09 (32)	−1.54 (7)	−0.95 (13)
Metabolism	IPR012347	Ferritin-like	1.42 (35)	0.1 (11)	−0.58 (5)	−0.44 (6)	−0.73 (4)	−1.09 (2)	−0.44 (6)
Metabolism	IPR036849	Enolase-like, C-terminal domain	1.28 (50)	−0.25 (14)	−0.18 (15)	−0.41 (12)	0.08 (19)	−1.04 (6)	−0.92 (7)
Metabolism	IPR036909	Cytochrome c-like domain	1.22 (39)	−0.43 (9)	−0.78 (6)	−0.15 (12)	0.22 (17)	−0.66 (7)	−0.66 (7)
Metabolism	IPR036226	Lipoxygenase, C-terminal domain	1.16 (86)	0.45 (51)	−0.14 (32)	−0.45 (25)	0.01 (36)	−1.18 (13)	−1.77 (7)
Metabolism	IPR037069	Acyl-CoA dehydrogenase/oxidase, N-terminal domain	1.13 (36)	−0.53 (8)	−0.23 (11)	−0.14 (12)	0.29 (18)	−0.91 (5)	−0.77 (6)
Metabolism	IPR032466	Metal-dependent hydrolase	1.11 (94)	−0.71 (23)	−0.47 (28)	0.1 (44)	0.37 (54)	−1.19 (15)	−0.56 (26)
Metabolism	IPR036396	Cytochrome P450	0.37 (745)	0.35 (732)	0.26 (688)	−0.02 (568)	0.06 (600)	−0.86 (314)	−0.66 (361)

*Parenthesized numbers indicate the number of genes categorized in each superfamily. Tc, *Tanacetum cinerariifolium*; Cs, *Chrysanthemum seticuspe*; Aa, *Artemisia annua*; Ha, *Helianthus annuus*; Nt,* Nicotiana tabacum*; Os, *Oryza sativa*; At, *Arabidopsis thaliana*.

For biodefense-related superfamilies, “Ribosome-inactivating protein (RIP)” (IPR036041) and “Ricin B-like lectins” (IPR035992), both of which are categorized as type II RIPs, showed multiplication odds scores of 2.05 and 1.57 in *T. cinerariifolium*, respectively (Table 6). RIPs, including ricin, show high toxicity to a wide range of species, serving as biodefense molecules for the producing plant. For example, tobacco RIP, isolated and purified from *N. tabacum* leaves, has been reported to possess strong antibacterial activity against *Pseudomonas solanacearum*, *Erwinia amylovora*, and *Shigella asonei*^34^. Pokeweed antiviral protein, purified from the extracts of plant leaves of *Phytolacca americana*^35^, has been reported to enhance this plant’s systemic resistance to tobacco mosaic virus infection^36^. *Sambucus nigra* agglutinin I (SNA-I) has been shown to exhibit insecticidal potency against Hemiptera^37^. Indeed, a BLASTP search of SNA-I sequence against *T. cinerariifolium* RIPs detected Tci_399175 with an E-value score of 2.99 × 10^−112^ and 37.89% identity (Supplemental Fig. 3A). Sequence alignment and BLASTP results revealed that Tci_399175 harbors the well conserved RIP domain and two RICIN domains containing Q-X-W motifs (Supplemental Fig. 3A). In other work, RIP domains have been implicated in the cleavage of rRNA glycosidic bonds, while RICIN domains have been shown to be involved in internalization via binding of target cell glycans^38^. This sequence analysis suggested that the *T. cinerariifolium*-enriched RIPs include insecticides similar to SNA-I. These findings indicated that *T. cinerariifolium* possesses not only low-molecular-weight insecticides such as sesquiterpene lactones^39^ and pyrethrins^2,3^ but also a greater number of genes (compared with other plants) encoding native proteins with toxicity against insects.

For signaling-related superfamilies “Signal transduction histidine kinase, dimerization/phosphoacceptor domain” (IPR036097), “Rho GDP-dissociation inhibitor domain” (IPR024792), and “HECT, E3 ligase catalytic domain” (IPR035983) showed specific multiplication in *T. cinerariifolium*, with multiplication scores of 1.39, 1.32, and 1.14, respectively (Table 6). Some plant genera are known to possess histidine kinases (proteins that act as ethylene receptors) as well as non-ethylene-receptor histidine kinases (e.g., cytokinin receptors)^40^. A BLASTP search using *A. thaliana* ethylene-response 1 (ETR1)^41^, an ethylene-activated kinase, as a query detected Tci_144982, a predicted protein that includes a G-X-G motif-containing HATPase_c domain, with an E-value score of 0 and 69.18% identity (Supplemental Fig. 3B). This sequence analysis suggested that *T. cinerariifolium*-enriched histidine kinases include ethylene-receptor-type histidine kinases similar to ETR1. These *T. cinerariifolium*-specific multiplications of signaling molecules suggested that *T. cinerariifolium* has genus-specific enrichment of signal transduction and regulation proteins. While the molecular mechanisms of regulation of the accumulation and composition of pyrethrins are yet to be defined, the products of these enriched signaling-related genes are obvious candidates for regulators of pyrethrin biosynthesis.

The metabolism-related superfamily showed the strongest gene multiplication, with genes encoding “Enolase-like, C-terminal domain” (IPR036849), “Cytochrome c oxidase-like domain” (IPR036909) “Lipoxygenase, C-terminal domain” (IPR036226), “Acyl-CoA dehydrogenase/oxidase N-terminal domain” (IPR037069), and “Metal-dependent hydrolase, composite domain” (IPR011059) exhibiting multiplication scores of 1.28, 1.22, 1.16, 1.13, and 1.11, respectively (Table 6). Since lipoxygenases (e.g., TcLOX1) and acyl-CoA dehydrogenases/oxidases (enzymes catalyzing reactions related to the beta-oxidation of 12-oxo-cis-10,15-phytodienic acid) are known to be involved in the oxylipin pathway leading to the synthesis of pyrethrin alcohol moieties (Fig. 1B), these multiplication scores are compatible with the specific biosynthesis of pyrethrins and jasmonates in *T. cinerariifolium*. Furthermore, “Cytochrome P450” (IPR036396) was detected with a multiplication score of 0.37, representing a total of more than 745 genes in *T. cinerariifolium* and making the corresponding loci the most abundant class of genes in this plant. Indeed, TcJMH, a protein that is categorized as a P450, is known to be involved in the biosynthesis of jasmolone (Fig. 1B); thus, the multiplication of P450s is consistent with a role of this protein class in the *T. cinerariifolium*-specific biosynthesis of jasmonates. The ferritin-like superfamily, members of which are responsible for iron transport, was found to have a multiplication score of 1.42. In *A. thaliana*, ferritins 1, 3, and 4 have been shown to regulate the expression of multiple genes encoding P450 enzymes^42^. A BLASTP search with *A. thaliana* ferritin 1 (AtFer-1) against *T. cinerariifolium* ferritin-like proteins detected Tci_154278 with an E-value of 5.64 × 10^−115^ and 69% identity, including a conserved eukaryotic ferritin domain-containing iron ion channel, a ferroxidase diiron center, and a ferrihydrite nucleation center (Supplemental Fig. 3C). In this regard, the multiplied genes encoding ferritin-like proteins in *T. cinerariifolium* are expected to include proteins that modulate the expression of various P450s, presumably including one or more that affect TcJMH expression. This result is consistent with the duplication of genes encoding metalloenzymes such as metal-dependent hydrolases, cytochrome c, aconitases, lipoxygenases, and P450s (Table 6), and suggests that *T. cinerariifolium* likely needs to import larger amounts (compared with other plants) of metals for use in various enzyme reactions and their regulation.

The lowest multiplication scores were found with “Expansin, cellulose-binding-like domain” (IPR036749), “Mitochondrial glycoprotein” (IPR036561), and “ArfGAP domain” (IPR038508), which exhibited multiplication scores of −0.53, −0.53, and −0.49, respectively (Table 7). The multiplication scores of these superfamilies in *O. sativa*, *C. seticuspe*, *A. annua*, and *H. annuus* were as low as the multiplication scores in *T. cinerariifolium* (Table 6), indicating that the depletion of the genome for members of this superfamily was not unique to *T. cinerariifolium* among the Asteraceae. In contrast, “Endochitinase-like”, a superfamily that plays a pivotal role in defense against fungal pathogens^43^, exhibited a *T. cinerariifolium*-specific low-copy status. The accumulation of RIPs (Table 6) and depletion of endochitinases suggested that the corresponding biodefense strategy has evolved specifically in *T. cinerariifolium*.

**Table 7:** Superfamilies with lowest multiplication odds scores in *T. cinerariifolium*.

Category	InterPro ID	Superfamily name	Tc	Cs	Aa	Ha	Nt	Os	At
Biodefense	IPR036861	Endochitinase-like	−1.15 (1)	−0.41 (5)	−0.28 (6)	0.51 (14)	0.26 (11)	0.07 (9)	0.35 (12)
Non-specific	IPR036749	Expansin, cellulose-binding-like domain	−0.53 (36)	−0.53 (36)	−0.08 (51)	0.02 (55)	0.61 (85)	0.18 (62)	−0.05 (52)
Non-specific	IPR036561	Mitochondrial glycoprotein	−0.53 (6)	−0.29 (8)	−0.29 (8)	−0.29 (8)	0.53 (18)	−0.09 (10)	0.53 (18)
Non-specific	IPR038508	ArfGAP domain	−0.49 (24)	−0.65 (21)	−0.3 (28)	0.56 (55)	0.67 (60)	−0.96 (16)	0.3 (45)

*Parenthesized numbers indicate the number of genes categorized in each superfamily. Tc, *Tanacetum cinerariifolium*; Cs, *Chrysanthemum seticuspe*; Aa, *Artemisia annua*; Ha, *Helianthus annuus*; Nt,* Nicotiana tabacum*; Os, *Oryza sativa*; At,* Arabidopsis thaliana*.

### Phylogenetic analysis of pyrethrin-related enzymes

One of the most characteristic features of *T. cinerariifolium* is the presence of the pyrethrin biosynthetic pathway, components of which include TcLOX1 (*T. cinerariifolium* 13-lipoxygenase), TcJMH (*T. cinerariifolium* jasmone hydroxylase), TcCDS (*T. cinerariifolium* chrysanthemyl diphosphate synthase), and TcGLIP (*T. cinerariifolium* GDSL (Gly-Asp-Ser-Leu motif) lipase). TcLOX1 and TcJMH are related to the lipoxygenase and P450 superfamilies, classes that were shown (by InterProScan analysis) to be enriched in *T. cinerariifolium*. On the other hand, since TcCDS (unlike typical terpene synthases) has non-head-to-tail cyclase activity with isoprenoid units and TcGLIP (unlike typical GDSL lipases) has acyltransferase activity, there are no corresponding InterProScan superfamilies into which these enzymes can be categorized in terms of function. Therefore, we further analyzed the expansion of genes encoding TcLOX1, TcJMH, TcCDS, and TcGLIP using a BLAST search against the predicted proteomes of 13 different plant species available on public databases, and performed a molecular phylogenetic analysis using a maximum-likelihood tree with 500 bootstraps.

**Figure 5:**
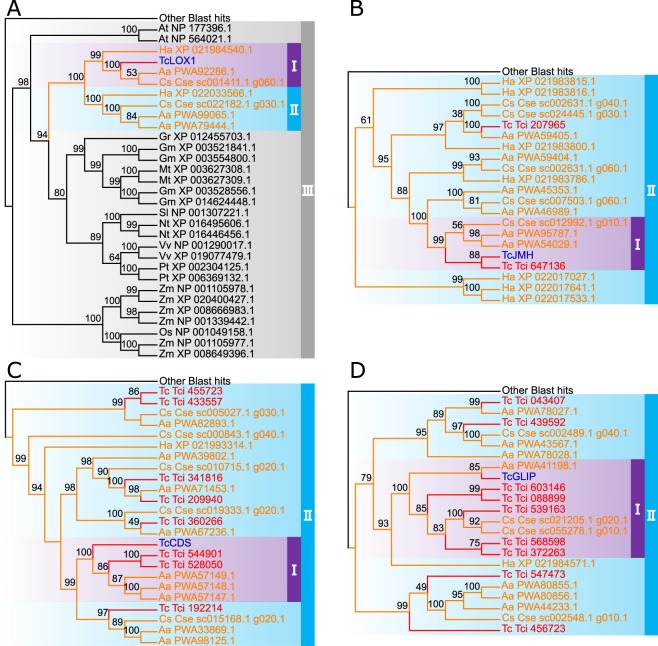
Enlarged view of molecular phylogenetic tree of pyrethrin-related enzymes. Clades I, II, and III for enzymes with similarity to *T. cinerariifolium* 13-lipoxygenase (TcLOX1) (**A**), *T. cinerariifolium* jasmone hydroxylase (TcJMH) (**B**), *T. cinerariifolium* chrysanthemyl diphosphate synthase (TcCDS) (**C**), and *T. cinerariifolium* GDSL lipase (TcGLIP) (**D**) are shown based on the results provided in Supplemental Figs. 4–7, respectively. The nodes for pyrethrin-related enzymes are indicated in blue; *T. cineariifolium* nodes and edges are indicated in red; other Asteraceae nodes and edges are indicated in orange.

TcLOX1 is a member of a class of widely prevalent plant enzymes; TcLOX1 has been shown to be responsible for production of the alcohol moieties of pyrethrins via conversion of linolenic acid to 13-hydroperoxylinolenic acid^7^ (Fig. 1B). A BLASTP search using the TcLOX1 sequence as a query detected 134 proteins, including 9 *T. cinerariifolium* proteins, 29 other Asteraceae proteins, and 96 non-Asteraceae proteins, with 42.03–57.58%, 42.31–95.26%, and 35.09–75.38% identity to TcLOX1, respectively. Molecular phylogenetic analysis of these proteins revealed that TcLOX1 formed an orthologous clade with *C. seticuspe, A. annua*, and *H. annuus* proteins (Fig. 5A and Supplemental Fig. 4, clade I). This clade was positioned proximally to another clade consisting only of Asteraceae proteins (Fig. 5A and Supplemental Fig. 4, clade II), indicating that these LOX families were duplicated in Asteraceae. In addition, these clades (Fig. 5A and Supplemental Fig. 4, Clades I and II) belonged to a larger lipoxygenase clade (Fig. 5A and Supplemental Fig. 4, clade III) that included *A. thaliana* LOX3 and 4, which have the same activity as TcLOX1. This LOX clade was consistent with the report that Asteraceae plants share the linolenic acid lipoxygenase activity^7^.

TcJMH catalyzes the conversion of jasmone to jasmolone^8^ (Fig. 1B), which has been reported only in the pyrethrin-synthesizing genus. A BLASTP search using the TcJMH sequence as a query detected 136 proteins, including 9 *T. cinerariifolium* proteins, 30 other Asteraceae proteins, and 97 non-Asteraceae proteins with 34.22–81.53%, 37.67–85.01%, and 36.44–58.01% identity to TcJMH, respectively. Molecular phylogenetic analysis of these proteins revealed that TcJMH formed a subclade with one other *T. cinerariifolium* protein, two *A. annua* proteins, and one *C. seticuspe* protein (Fig. 5B and Supplemental Fig. 5, Clade I) within a large clade consisting of two *T. cinerariifolium* proteins and 18 Asteraceae proteins (Fig. 5B and Supplemental Fig. 5, Clade II). This inclusion within a large clade is in contrast with the fact that the conversion of jasmone to jasmolone has been reported only in the *Tanacetum* genus^8^. These results suggested that such non-*Tanacetum* TcJMH-like protein genes are non-functional or are involved in a pathway for biosynthesis of phytochemicals other than those observed in *T. cinerariifolium*.

TcCDS participates in the formation of terpenoid acid moieties by conversion of dimethylallyl diphosphate to chrysanthemyl diphosphate^9^ (Fig. 1B), which also has been reported to be a genus-specific pyrethrin-synthesizing reaction. A BLASTP search using the TcCDS sequence as a query detected 122 proteins, including 9 *T. cinerariifolium* proteins, 28 other Asteraceae proteins, and 85 non-Asteraceae proteins with 61.27–88.54%, 36.36–93.52%, and 20.21–66.57% identity to TcCDS, respectively. Molecular phylogenetic analysis of these proteins revealed that TcCDS formed a subclade with three *A. annua* proteins and three other *T. cinerariifolium* proteins (Fig. 5C and Supplemental Fig. 6, Clade I), within a large cluster consisting of seven *T. cinerariifolium* proteins and 17 Asteraceae proteins (Fig. 5C and Supplemental Fig. 6, Clade II). The phylogenetic tree for TcCDS-like proteins did not include explicit orthologous clades; this result was consistent with a study demonstrating that the production of genus-specific monoterpenes, including chrysanthemyl diphosphate, has diverged in individual Asteraceae genera^44^. Previously, *A. annua* was shown to have a large number of terpene-related loci in its genome; the products of these genes are responsible for the biosynthesis of various genus-specific terpenes^13^. These findings support the view that these terpene-biosynthesis genes also have multiplied and diverged in *T. cinerariifolium*, as reported for those of *A. annua*^13^.

TcGLIP synthesizes pyrethrin I by esterification of chrysanthemic acid moieties and pyrethrolone moieties in reactions specific to the pyrethrin-synthesizing genus^1^ (Fig. 1B). A BLASTP search using the TcGLIP sequence as a query detected 135 sequences, including 9 *T. cinerariifolium* proteins, 30 other Asteraceae proteins, and 96 non-Asteraceae proteins with 48.73–81.78%, 31.87–79.35%, and 30.38–53.35% identity to TcGLIP, respectively. Molecular phylogenetic tree analysis of these proteins revealed that TcGLIP formed a subclade with one *A. annua* protein, two *C. seticuspe* proteins, and five other *T. cinerariifolium* proteins (Fig. 5D and Supplemental Fig. 7, Clade I), within a large cluster consisting of nine *T. cinerariifolium* proteins and 12 Asteraceae proteins (Fig. 5D and Supplemental Fig. 7, Clade II). This phylogenetic tree demonstrated that *T. cinerariifolium* has massively multiplied TcGLIP-related proteins.

### Distribution analysis of pyrethrin-related enzymes

**Figure 6:**
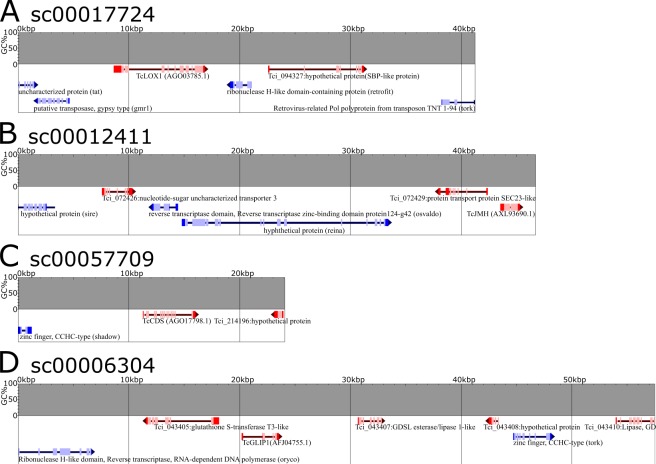
Scaffolds including loci encoding pyrethrin-related enzymes. The predicted genes on the scaffolds containing genes encoding *T. cinerariifolium* 13-lipoxygenase (TcLOX1) (**A**), *T. cinerariifolium* jasmone hydroxylase (TcJMH) (**B**), *T. cinerariifolium* chrysanthemyl diphosphate synthase (TcCDS) (**C**), and *T. cinerariifolium* GDSL lipase (TcGLIP) (**D**) were visualized using GenomeJack. %GC content, transposable element (TE) coding regions, and non-TE coding regions are shown as bar-graphs, blue arrows, and red arrows, respectively. Red and pink boxes on non-TE gene arrows represent exons and protein-coding regions, respectively. Blue and sky-blue boxes on TE gene arrows represent exons and protein-coding regions, respectively.

Recent studies have shown that genes encoding components of a particular metabolic pathway, especially those responsible for specialized plant metabolites, are genomically co-localized and co-regulated as Metabolic Gene Clusters (MGCs)^45,46^. To find genes co-localized with pyrethrin-related MGCs, we initially subjected the genome sequences to PhytoClust^46^ with default settings for cluster range and flanking region. However, no MGCs that included loci encoding pyrethrin biosynthesis-related enzymes were detected in the *T. cinerariifolium* genome. To permit further analysis, the distribution of genes within the scaffolds that included loci encoding TcLOX1, TcJMH, TcCDS and TcGLIP were analyzed using the GenomeJack software program. The TcLOX1-encoding gene was located on scaffold sc00017724 and showed co-localization with one gene encoding a hypothetical protein (Tci_094327) and four TE genes (Fig. 6A). A BLASTP search using the Tci_094327 sequence as a query detected the *Artemisia annua* squamosa promoter-binding (SPB) transcription factor (PWA57483.1) with an E-value of 2 × 10^−35^ and 52.35% identity (Supplemental Fig. 8A). Since the *A. annua* SPB transcription factor regulates the expression of proteins responsible for artemisinin B biosynthesis^47^, Tci_094327 is a candidate regulator of the oxylipin pathway. The TcJMH-encoding gene was positioned on scaffold sc00012411 and showed co-localization with two genes encoding putative transporters (Tci_072426 and Tci_072429) and three TE genes (Fig. 6B). The TcCDS-encoding gene was positioned on scaffold sc00057709 and exhibited co-localization with the gene encoding Tci_214196 (a protein that lacks apparent homologs by BLASTP) and one TE gene (Fig. 6C). These results indicated that genes encoding other pyrethrin-associated enzymes were not distributed proximal to the loci encoding either TcJMH or TcCDS. In contrast, the gene encoding TcGLIP was positioned on scaffold sc00006304 and co-localized with loci encoding two GDSL family lipases (Tci_043407 and Tci_043410), one glutathione S-transferase (Tci_043405), one hypothetical protein (Tci_043408), and two TE genes (Fig. 6D). Tci_043407 and Tci_043410 showed 50.53% and 41.10% identity to TcGLIP (Supplemental Fig. 8B), respectively, suggesting that these genes had been generated by gene duplication and might encode enzymes with activities similar to that of TcGLIP. Thus, our results collectively indicated that a gene encoding the SPB transcription factor was proximal to the locus encoding TcLOX, and that genes encoding two GDSL lipases lie proximal to the locus encoding TcGLIP, suggesting that these novel genes may be co-regulated with those encoding pyrethrin-related enzymes known to be involved in pyrethrin biosynthesis.

## Discussion

*T. cinerariifolium* is characterized by species-specific biosynthesis of the pyrethrins, a class of potent natural insecticides that are non-harmful to humans. A wide variety of specialized metabolites, including the pyrethrins, has been found to play defensive roles against several threats such as pests, pathogens, and herbivores^2,3,13,48^. Furthermore, the markedly larger genome size (7.1 Gb) of *T. cinerariifolium*, compared with those of other plants of the Asteraceae genera (2.72 Gb for *C. seticuspe*^12^, 1.74 Gb for *A. annua*^13^, and 3.6 Gb for *H. annuus*^14^), suggests the occurrence of *T. cinerariifolium*-specific evolutionary processes. To address these issues, the present study explored the draft genome sequence of *T. cinerariifolium*. In general, the assembly of large-sized genomes, such as that of *T. cinerariifolium*, is frequently hindered or precluded by numerous sequence errors due to inappropriate sequencing methods and the presence of repetitive sequences^17^. Therefore, we sequenced the *T. cinerariifolium* genome to high depth, including 211-fold coverage by PE reads (to provide error correction) and 56-fold coverage by MP reads (to permit the assembly of repetitive sequences) (Table 1). Furthermore, PANDAseq (Fig. 2) also has been shown to be effective for large-genome assembly, since this program includes Illumina-optimized error correction and longer-read generation using overlapping paired-end information^49^. The assembly strategy with high-depth reads used in the present paper eventually elucidated a 7.1-Gb genome sequence with a N50 of 14 Kb and 91.8% completeness as judged by BUSCO analysis (Tables 2 and 3). The resultant genome sequence revealed that TEs and multiple superfamily genes are enriched in the *T. cinerariifolium* genome.

The *sire*- and *oryco*-clade TEs were shown to have accumulated after the divergence of *T. cinerariifolium* from other Asteraceae (Fig. 3, and Supplemental Figs. 1 and 2), with TEs of these clades occupying 11.16 and 2.14% of the genome sequence, respectively (Table 4). This accumulation of the *sire*- and *oryco*-clade TEs contributes to the large size of the *T. cinerariifolium* genome. Furthermore, TEs have been suggested to be involved in the multiplication of plant disease resistance-related genes in *Capsicum*^50^. In this context, the accumulation of the *sire*- and *oryco*-clade TEs in *T. cinerariifolium* also may contribute to the *T. cinerariifolium*-specific enrichment of signaling-, biodefense-, and biosynthesis-related enzymes, as detected by InterProScan analyses.

The *T. cinerariifolium*-enriched signaling-related superfamily genes included those encoding histidine kinases (Table 6), a group shown (using a BLASTP search and sequence alignment; Supplemental Fig. 3B) to include ethylene-receptor type histidine kinases similar to *A. thaliana* ETR1. These results suggested that *T. cinerariifolium*-enriched histidine kinases include proteins involved in responses to air-borne compounds. Notably, *T. cinerariifolium* upregulates pyrethrin biosynthesis by emitting a specific combination of volatile organic compounds (VOCs) when the plant body has been wounded^51,52^, suggesting that the upregulation of components of the pyrethrin biosynthetic pathway by wound-induced VOCs may occur via histidine kinase-mediated signal induction. Moreover, genes encoding lipoxygenases, which are known to contribute to the biosynthesis of VOCs^53^, also were shown to be enriched in a genus-specific fashion in *T. cinerariifolium* (Table 6). These findings provide crucial clues to the investigation of the VOC-dependent regulation of pyrethrin biosynthesis.

The *T. cinerariifolium*-enriched biodefense-related superfamily genes included those encoding ribosome inactivating proteins (RIPs) (Table 6), which are toxic to herbivores^37^. Furthermore, a BLASTP search and sequence alignment also suggested that *T. cinerariifolium*-enriched RIPs include insecticidal proteins similar to SNA-I (Supplemental Fig. 3A). These results indicated that *T. cinerariifolium* has the potential to produce such toxic proteins as biodefense factors, in addition to the production of insecticidal phytochemical toxins such as the pyrethrins. In contrast, the *T. cinerariifolium* genome contains only a single gene encoding a putative endochitinase (Table 7), a class of proteins known to be involved in defense against fungal pathogens^43^. This observation accords with the low resistance of *T. cinerariifolium* against serious foliar diseases caused by fungi such as *Stagonosporopsis tanaceti* and *Didymella tanaceti*^54^, leading to the need for care in preventing fungal pathogen infection during the cultivation of *T. cinerariifolium*. Altogether, the present results support the view that *T. cinerariifolium* might have been adapted to the dry environment of its area of origin, the Balkans, where fungi are less numerous than in the areas of the plant’s current cultivation. Consequently, this plant is likely to have been endowed with multiple RIPs as potent defenses against herbivores in addition to the acquisition of pyrethrins during the evolutionary processes.

*T. cinerariifolium* also was found to be enriched for genes encoding members of the lipoxygenase and P450 superfamilies (Table 6), which are involved in the pyrethrin biosynthetic pathway. The existence of unique genes related to pyrethrin biosynthesis is one of the most prominent features of the *T. cinerariifolium* genome. Molecular phylogenetic analyses of the enzymes for pyrethrin biosynthesis demonstrated that paralogs of TcGLIP, which synthesizes pyrethrin I (Fig. 1B), clustered in a *T. cinerariifolium*-specific clade (Fig. 5D and Supplemental Fig. 7, clade II), while paralogs of TcJMH and TcCDS, which synthesize jasmolone and chrysanthemyl diphosphate, respectively (Fig. 1B), clustered in Asteraceae-specific clades (Fig. 5B,C and Supplemental Figs. 5 and 6, clade II). In contrast, TcLOX1-related enzymes, which are widely prevalent plant enzymes responsible for the synthesis of 13-hydroperoxylinolenic acid (Fig. 1B), clustered in a clade including non-Asteraceae proteins (Fig. 5A and Supplemental Fig. 4, clade III). In particular, genes encoding TcCDS-related enzymes, which are categorized as terpenoid synthases, were as multiplied and varied in *T. cinerariifolium* as in *A. annua*, where this class of proteins is known to biosynthesize various genus-specific terpenes^13^ (Fig. 5C and Supplemental Fig. 6). Consequently, these multiplied genes encoding terpenoid-biosynthetic enzymes of *T. cinerariifolium* are consistent with *T. cinerariifolium*-specific terpenoid synthesis. In contrast, TcJMH, the key enzyme for synthesis of the alcohol moieties of the pyrethrins (Fig. 1B), was shown to be highly conserved in other plants (Fig. 5B and Supplemental Fig. 5), whereas the alcohol moieties of pyrethrins, including jasmolone, pyrethrolone, and cinerolone, have been identified only in members of the *Tanacetum* genus^1–3^. These results raise the question whether these alcohol moieties have not yet been identified in non-*Tanacetum* plants, or whether TcJMH-like proteins can act on other substrates.

InterProScan analysis revealed that the *T. cinerariifolium* genome is enriched for genes encoding members of the ferritin-like superfamily, proteins that are responsible for iron transport. Furthermore, various iron enzymes, including members of the lipoxygenase and P450 superfamily, a class that includes enzymes of the pyrethrin-biosynthesis pathway (including TcLOX1, TcJMH, and a putative P450 for conversion of jasmolone to pyrethrolone^55^) also were shown to be multiplied (Table 6). Indeed, AtFer-1 is reported to regulate the expression of P450 in *A. thaliana*^42^, and *T. cinerariifolium* is enriched for genes encoding ferritins, included a locus encoding a ferritin similar to AtFer-1 (Supplemental Fig. 3C, Tci_154278). Taken together, these results support the view that the ferritin-like superfamily and pyrethrin-biosynthetic lipoxygenase and P450s might have co-evolved in this lineage, given these proteins’ complementary roles in the uptake and biochemical use of iron. Thus, further work will need to examine the functional correlation of these multiplied ferritin-like superfamily proteins with multiplied genus-specific pyrethrin-biosynthesis enzymes.

Molecular phylogenetic analysis of TcGLIP, which plays a critical role in the esterification reaction that serves as the final step in the biosynthesis of pyrethrin I (Fig. 1B), revealed that genes encoding TcGLIP-related proteins have diverged in the genome of *T. cinerariifolium* (Fig. 5D and Supplemental Fig. 7). These results suggest that TcGLIP (and potentially other related proteins) acquired a role in esterification of pyrethrins in the specific lineage of this genus. Previously, the catalyzing activity of recombinant TcGLIP for the esterification of pyrethric acid with pyrethrolone was found to be lower than that of pyrethric acid with jasmolone, pyrethric acid with cinerolone, chrysanthemic acid with pyrethrolone, chrysanthemic acid with jasmolone, and chrysanthemic acid with cinerolone^1^. Combined with those previous findings, the results of the present study suggested that the newly detected TcGLIP-family proteins likely catalyze uncharacterized esterification reactions (Fig. 1B). Collectively, the present genomic and molecular phylogenetic analyses are expected to contribute to the verification of pathways leading to the biosynthesis of unidentified pyrethrins (e.g., the conversion of jasmolone to pyrethrolone) and the underlying regulatory mechanisms. Furthermore, the molecular data obtained in the present study are expected to facilitate the creation of transgenic and genome-edited *T. cinerariifolium*, paving the way to metabolic engineering for efficient phytochemical production, including that of additional pyrethrins.

The information on the co-localization (within the genome) of loci encoding related metabolic activities also is important, given that genes encoding components of a particular metabolic pathway, especially for specialized plant metabolites, frequently form such Metabolic Gene Clusters (MGCs)^45,46^. Although no MGCs for the enzymes known to be involved in pyrethrin biosynthesis were detected, a gene encoding a squamosa promoter-binding (SPB) transcription factor-like protein was observed to be co-localized with the gene encoding TcLOX1 (Fig. 6A). The *A. annua* SPB transcription factor regulates genes encoding proteins involved in the biosynthesis of artemisinin B, an A*. annua*-specific terpenoid^47^. Since TcLOX1 is not involved in synthesis of the terpenoid subunit of pyrethrin but rather in the synthesis of the alcohol moieties of pyrethrins (Fig. 1), it will be interesting to see if this co-localized SPB transcription factor-encoding gene is involved in pyrethrin biosynthesis. Similarly, we infer that newly detected TcGLIP-family proteins likely catalyze uncharacterized esterification reactions (Fig. 1B). Genes encoding two GDSL lipases also were found to be co-localized with the locus encoding TcGLIP (Fig. 6D). These newly detected lipases are candidates for uncharacterized esterification reactions involved in the synthesis of cinerin I and II, jasmolin I and II, and/or pyrethrin II (Fig. 1B).

In conclusion, we have elucidated the draft genome of *T. cinerariifolum*, the natural source of the original mosquito coils. InterProScan and molecular phylogenetic analyses revealed the *T. cinerariifolum*-specific multiplication of genes encoding toxic proteins, metalloproteins, ferritins, histidine kinases, and pyrethrin biosynthetic enzymes, which are consistent with the unique physiological, ecological, and phytochemical features of these compounds. The draft genome opens new avenues for exploration of unidentified enzymes in pyrethrin biosynthesis and elucidation of the evolutionary processes of *T. cinerariifolum*, and provides fundamental information for the development of metabolic engineering of further beneficial phytochemicals using transgenic or genome-edited *T. cinerariifolum*.

## Materials and Methods

### Plant materials and genome sequencing

Wild-type *T. cinerariifolium* plants were grown and harvested under wild conditions in land surrounding the Onomichi City Museum of Art in Nishitsuchidocho, Onomichi, Hiroshima Prefecture, Japan. Genomic DNA was extracted from leaves and stems using a DNeasy Plant Mini Kit (Qiagen) according to the manufacturer’s instructions. Short-insert PE and MP libraries with three different insert sizes (3, 5 and 8 kb) of the extracted DNA were constructed using a TruSeq DNA PCR-Free kit (Illumina) and a Nextera Mate-Pair Sample Prep Kit (Illumina), respectively. The PE and MP libraries then were subjected to 150 × 2 cycles and 100 × 2 cycles of paired-end sequencing, using HiSeq X and HiSeq 4000 Illumina instruments, respectively.

### De novo assembly of genome sequences

The obtained read sequences were cleaned for assembly as described in our previous study^56^. Adapters derived from the Truseq or Nextera mate-pair Sample Prep Kit, low-quality reads, and short reads (<36 bp in length) were trimmed by using Trimmomatic version 0.36^57^.

Contig sequences were generated from the PE reads by a four-step process (“pre-assembly”, “SOAPdenovo assembly”, “clean-up”, and “merging with other assembly result”). First, the cleaned PE reads, including overlaps, were detected and pre-assembled by PANDAseq^49^. Second, these pre-assembled reads and remaining reads were subjected to SOAPdenovo v2.04-r240^18^ with multi k-mers from 80 to 127 to generate contig sequences. Third, the SOAPdenovo-generated contigs were cleaned by trimming the contigs showing more than 95% sequence identity with other contig sequences. Fourth, PE reads also were subjected to processing using the Platanus assembler 1.2.4^19^ with default parameters, and the generated contigs were merged with SOAPdenovo-constructed contigs using SSPACE-longread v1-1^20^, including setting minimum overlap length to 36 bp, minimum number of links to 1, and maximum link ratio to 0.5.

Scaffold sequences were generated by a three-step process (“read selection”, “scaffolding”, and “gapfilling”). First, PE and MP reads were mapped to contig sequences using bowtie version 2.3.4.3^58^ with the–local option; read pairs mapped discordantly were selected for the following steps. Second, the selected reads and contigs were subjected to scaffolding using SSPACE-STANDARD version 3.0^21^ (BaseClear), including setting the minimum number of links to 3. Third, the positions for which the bases were unknown (e.g., ‘N’) in the scaffolds were filled with ‘A/T/G/C’ by GapFiller v1-10^22^ (BaseClear) to complete the draft genome, setting. including setting the minimum number of overlaps to 30 and the number of reads to trim off to 50. Furthermore, the completeness of the draft genome was evaluated using BUSCO-v3^26^ with the embryophyta_odb9 protein set.

### Gene prediction and annotation

The *T. cinerariifolium* RNA-Seq data available on NCBI Sequence Read Archive (SRR2062279 and SRR2064180^24^, as well as SRR5985187-5985194^25^) were mapped to the assembled genome to generate the ‘intron hints’ for gene prediction. Subsequently, the assembled genome and ‘intron hints’ were subjected to Augustus 3.3.1^23^ trained with available *T. cinerariifolium* genes in GenBank. TEs in predicted genes were identified by hmmpfam in HMMER 2.3.1^31^ against GyDB 2.0^32^ with an E-value cutoff of 1.0. To identify high-confidence genes, the non-TE genes with known protein signatures were detected using InterProScan 5.6–48.0^33^ and annotated using Blast2GO^59^.

### Comparative analysis of TE content *versus* that in other plants

The TE content also was estimated for six other genome-elucidated plants, including *A. thaliana* (TAIR10^60^), *N. tabacum* (Ntab-TN90^61^)*, O. sativa* (assembly Build 4.0^62^)*, H. annuus* (HA412HO_v1.1^14^), *A. annua* (ASM311234v1^13^), and *C. seticuspe* (CSE_r1.0^12^). As described for TE detection in *T. cinerariifolium*, the coding regions of these genomes were estimated using Augustus 3.3.1^23^ with the *Arabidopsis* model set and the default parameters, and the TEs in these predicted transcriptomes were extracted using hmmpfam^31^ against GyDB^32^. According to GyDB^32^ classifications, the percentage of genomic regions occupied by each clade of TE was calculated as an accumulation score.

Molecular phylogenetic trees for *sire*- and *oryco*-clade TEs also were estimated using the ORTHOSCOPE method^63^ as described in our previous study^64^. The amino acid sequences of hmmpfam-extracted reverse-transcriptase domains encoded by the TEs in *T. cinerariifolium*, *H. annuus*, *A. annua*, and *C. seticuspe* were aligned with CLUSTAL W-mpi 0.13^65^, and maximum-likelihood phylogenetic trees based on a JTT matrix-based model^66^ with 100 bootstraps were created using Fast Tree 2.1.10 (JTT model, CAT approximation)^67^.

### Comparative analysis of protein superfamily content *versus* that in other plants

We also detected protein signatures using InterProScan^33^ analysis in six genome-elucidated plants, using methods as described above for the InterProScan analysis of *T. cinerariifolium*. Having determined the number of genes possessing each superfamily signature, the multiplication odds score for each combination of InterProScan-detected superfamily signature (Sig) and plant genus (Genus) were calculated as follows:

$${\rm{Multiplication}}\,{\rm{odds}}\,{\rm{score}}\,(\mathrm{Genus},\mathrm{Sig})={\log }_{2}\frac{N(Genus,Sig)+PS}{\overline{N(Sig)}+PS}$$

where N (Genus, Sig) represents the number of the plant-genus genes with an InterProScan-detected superfamily signature and PS represents the pseudo-count constant, which was set to 5.00.

In further analysis for functional proteins, we subjected the detected proteins of the superfamily with the highest multiplication odds score in each category to a BLASTP^68^ search (version 2.7.1). *S. nigra* agglutinin I (SNA-I, accession No. O22415.1), *A. thaliana* ethylene-response 1 (ETR1, accession No. AAA70047.1) and *A. thaliana* ferritin-1 (AtFer-1, accession No. CAA63932.1) were used as queries for ribosome-inactivating proteins, signal transduction histidine kinase proteins and ferritin-like proteins, respectively. The detected protein pairs (SNA-I and Tci_399175, ETR1 and Tci_144982, and AtFer-1 and Tci_154278) were subjected to sequence alignment using CLUSTAL W-MPI 0.13^65^.

Molecular phylogenetic trees were further estimated for *T. cinerariifolium* pyrethrin biosynthetic enzymes as described in our previous study^63,64^. Briefly, TcLOX1-, TcJMH-, TcCDS-, and TcGLIP-related proteins were identified using BLASTP 2.7.1^68^ to search the protein sequences of *A. thaliana* (TAIR10^60^), *N. tabacum* (Ntab-TN90^61^)*, O. sativa* (assembly Build 4.0^62^)*, H. annuus* (HA412HO_v1.1^14^), *A. annua* (ASM311234v1^13^), *C. seticuspe* (CSE_r1.0^12^), *Populus trichocarpa* (Pop_tri_v3^69^), *Medicago truncatula* (MedtrA17_4.0^70^), *Glycine max* (Glycine_max_v2.1^71^), *Zea mays* (B73_RefGen_v4^72^), *Gossypium raimondii* (Graimondii2_0^73^), *Solanum lycopersicum* (SL3.0)^74^, and *Vitis vinifera* (12X)^75^. The BLAST hit sequences were screened using an E-value cut-off of 10^−3^, and the top ten hits were used for the subsequent phylogenetic analyses. These sequences were aligned using CLUSTAL W-MPI 0.13^65^, and maximum-likelihood phylogenetic trees based on a JTT matrix-based model^66^ with 500 bootstraps were created using MEGA software^76^.

Metabolic Gene Clusters (MGCs) were investigated with PhytoClust^46^; this analysis required definition of variables corresponding to the span of chromosomal region in which genes are clustered (cluster range) and the length of region to be searched for additional marker enzymes (flanking region). We set both variables to 20 Kbp, in accordance with the values used in the previous report^46^. The genes included in the same scaffolds as those encoding pyrethrin-related enzymes (TcLOX1, TcJMH, TcCDS and TcGLIP) were visualized with the GenomeJack software program (Mitsubishi Space Software, Tokyo, Japan).

## Supplementary information

Supplementary figures 1-8

## Data Availability

The draft genome sequences and annotations, along with the raw reads for PE and MP, have been deposited in the DNA Data Bank of Japan (DDBJ) under the BioProject accession Code PRJDB8358.
