# Supplementary material for: Draft genome of Tanacetum cinerariifolium, the natural source of mosquito coil
Source: Sci Rep. 2019 Dec 3;9:18249. doi: 10.1038/s41598-019-54815-6 (PMC6890757; doi:10.1038/s41598-019-54815-6)
Supplement: Supplementary file 1 — Supplementary figures 1-8 [file 41598_2019_54815_MOESM1_ESM.pdf]

Supplementary material

Draft genome of *Tanacetum cinerariifolium*, the natural source of mosquito coil

Takanori Yamashiro[1¶], Akira Shiraishi[2¶], Honoo Satake[2] * Koji Nakayama[1] *

[1] Dainihon Jochugiku Co., Ltd., 1-1-11 Daikoku-cho, Toyonaka, Osaka 561-0827, Japan
[2] Bioorganic Research Institute, Suntory Foundation for Life Sciences, Kyoto 619-0284, JAPAN

¶These two authors contributed equally to this work.

***CORRESPONDING AUTHOR:**

Koji Nakayama
Address: Dainihon Jochugiku Co., Ltd., 1-1-11 Daikoku-cho, Toyonaka, Osaka 561-0827, JAPAN
E-mail: k.nakayama@kincho.co.jp
TEL: +81-6-6334-0001
FAX: +81-6-6334-0004

Honoo Satake
Address: Bioorganic Research Institute, Suntory Foundation for Life Sciences, 8-1-1 Seikadai, Seika-cho, Souraku, Kyoto 619-0284, JAPAN
E-mail: satake@sunbor.or.jp
TEL: +81-50-3182-0704
FAX: +81-774-98-6262

Supplemental figure 1.

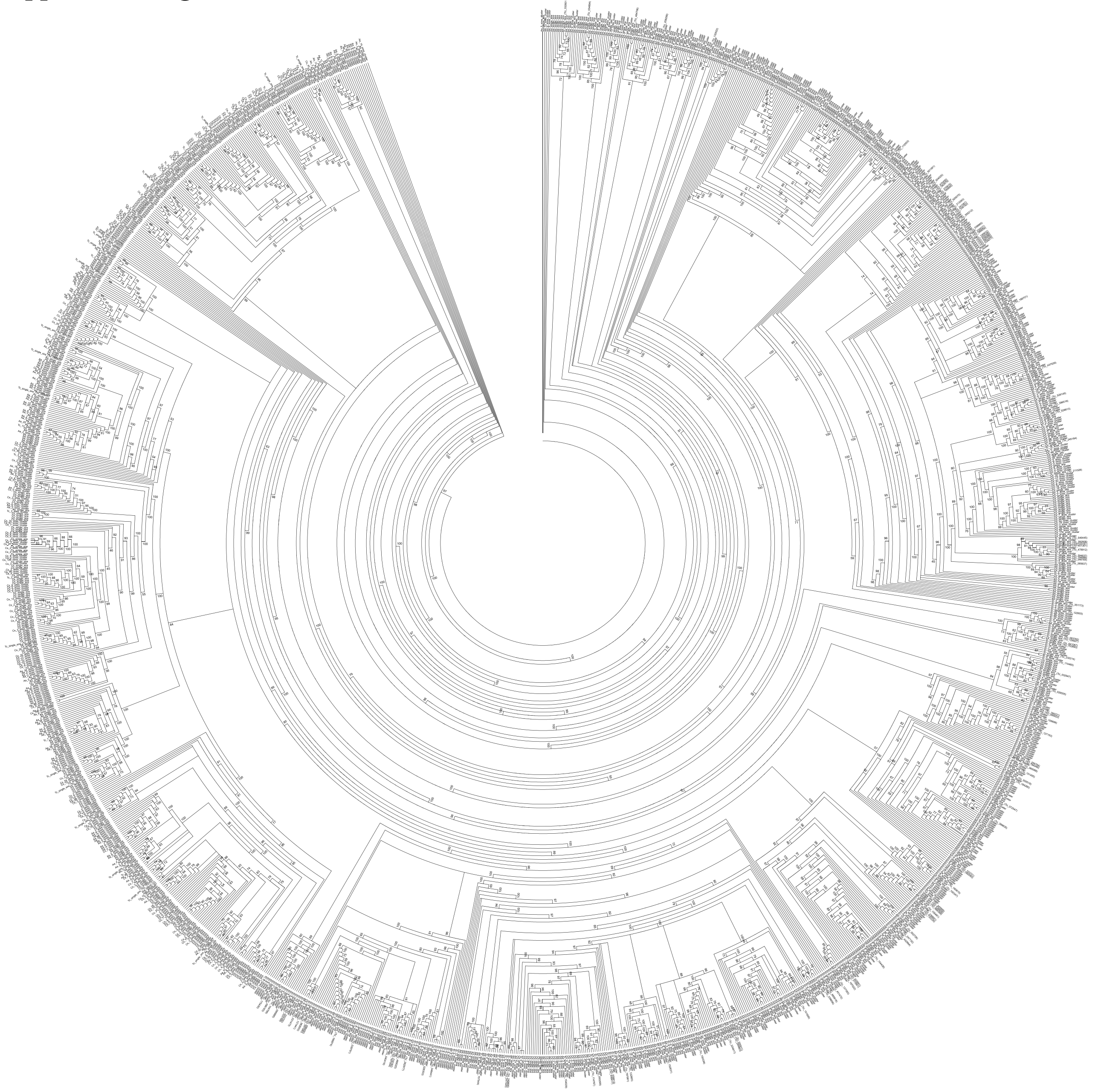

Supplemental Figure 1. Molecular phylogenetic analysis of *sire*-clade transposable elements (TEs). ML tree for sire TEs were generated based on RT domain-encoding sequences. The bootstrap-supported clusters with same-genus-derived genes are shown in single nodes.

Supplemental Figure 2.

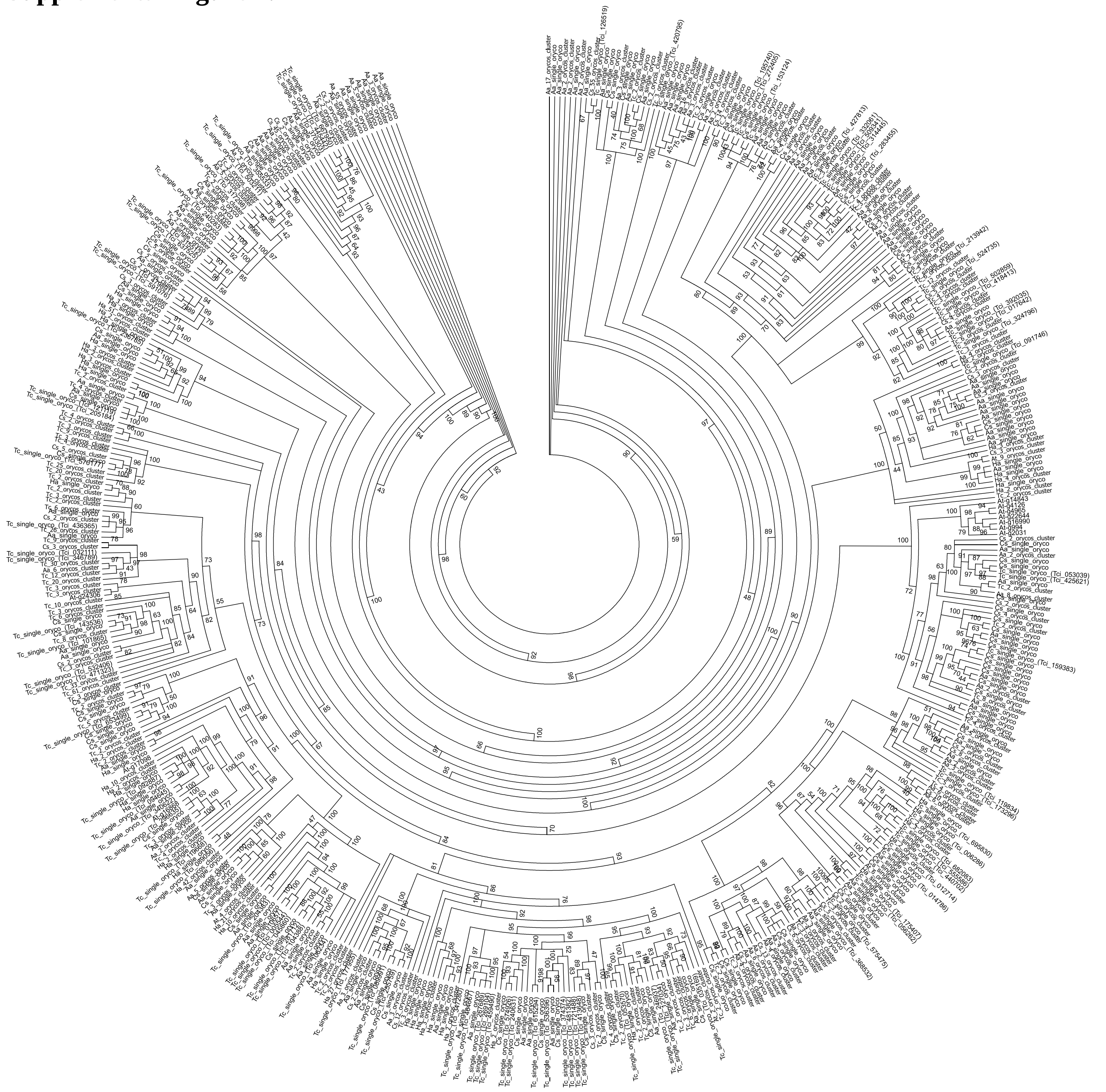

Supplemental Figure 2. Molecular phylogenetic analysis of *oryco*-clade transposable elements (TEs). ML tree for *oryco* TEs were generated based on RT-domain-encoding sequences. The bootstrap-supported clusters with same-genus-derived genes are shown in single nodes.

Supplemental Figure 3.

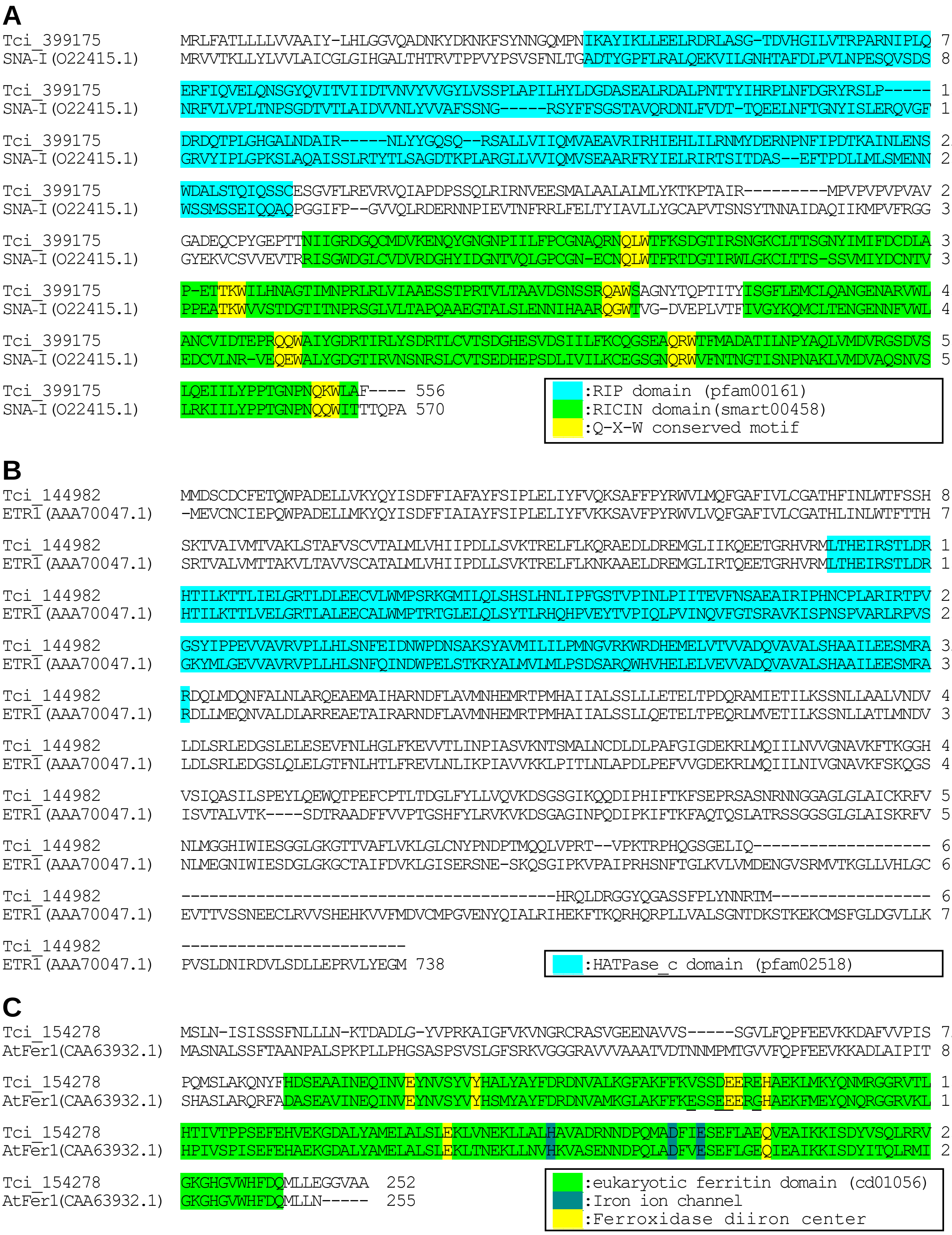

Supplemental Figure 3. Amino acid sequence alignment of ribosome-inactivating protein Tci_399175 and *Sambucus nigra* agglutinin I (SNA-I, accession No. O22415.1) (A), signal transduction histidine kinase Tci_144982 and *Arabidopsis thaliana* ethylene-response gene (ETR1, accession No. AAA70047.1) (B), and ferritin-like protein Tci_154278 and *A. thaliana* ferritin-1 (AtFer-1, accession No. CAA63932.1) (C). The conserved protein domains are highlighted or underlined.

Supplemental Figure 4.

A

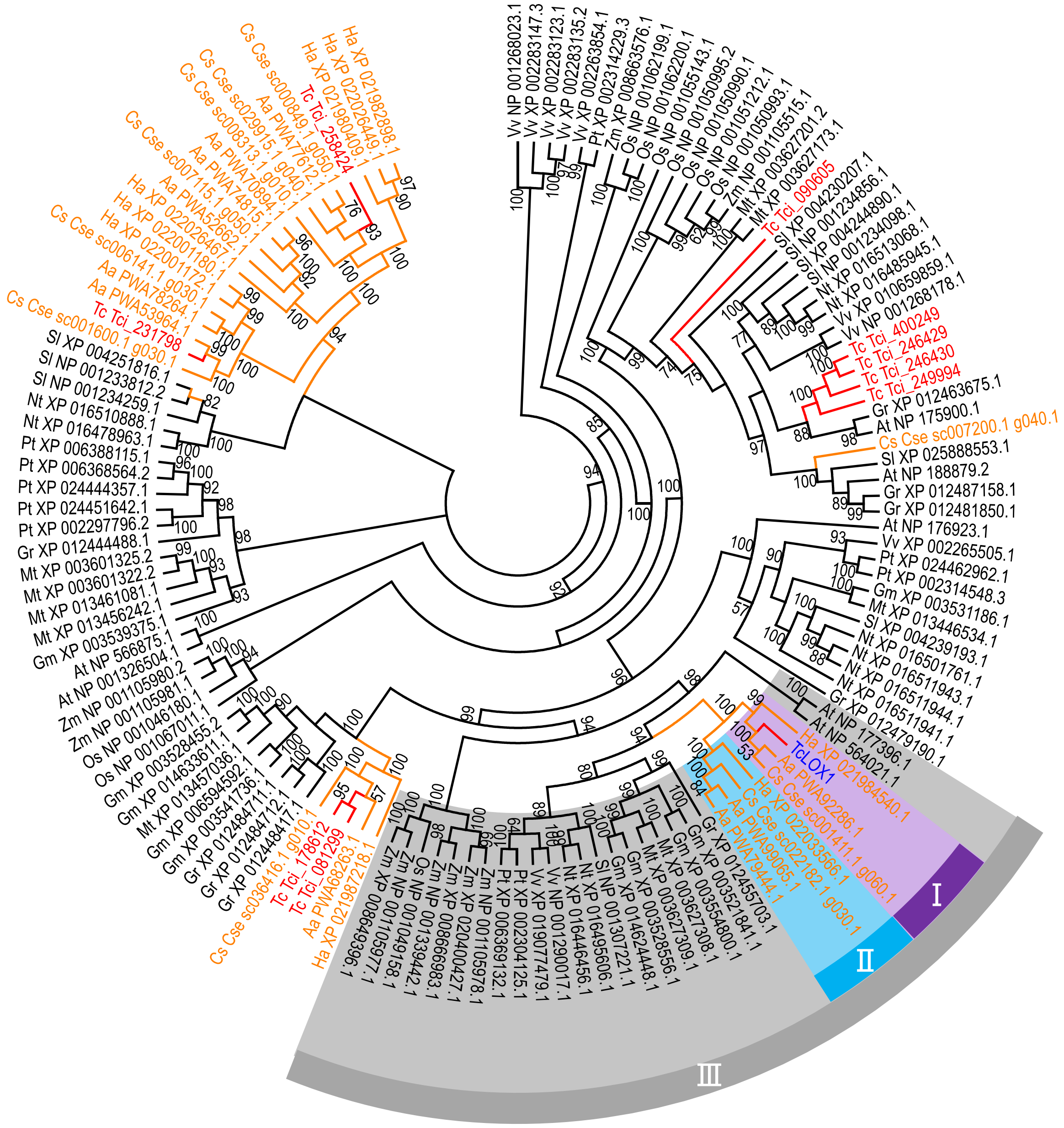

B

```
Aa_PWA79444.1           -MALAKELMGTSILQEKQ--PSFHNSK--SLKPMHH--NHVSISQNGSFQTRF----AKVVVKSAISEDIAKFVKSEKDKEKAVSFKVRAVLTVRNKVQE  89
Aa_PWA92286.1           -MALTKQIMGTS-LMDKK--TSVFGSN--------LCLNHVSLNKQHRLRLRK-TRKNGSMVVAAISEDLVRLVRVEK----PVTFKVRAVLTVRNKSKE  83
Aa_PWA99065.1           -MALAKELMGTSILQEKQ--PSFHNSK--SLKPMHQ--NHVSISQNGSFQTRF----GKVVVKSAISEDIAKFVKSEKDKEKAVSFKVRAVLTVRNKVQE  89
Cs_Cse_sc001411.1_g060.1-MALTKQIMGTS-LMDKK--TSVFGSN--------LCLNHVSINRQHRLRLRK-TRKNGSMVVAAISEDLVKFVRVEK----PVTFKVRAVLTVRNKSKE  83
Cs_Cse_sc022182.1_g030.1-MALAKELMGTSILQEKQQQPSFLN----SLKPMHQ--NHISISQNGSFRIR-----TKVVVKSAISEDIAKFVKSEKDKEKAVTFKVRAVLTVRNKVQE  88
Ha_XP_021984540.1       MASLAKDIISASSMVDNK--SSFFGSNIINQNLGRLYVNPVLIPLDRKRRLRKGVRTTTPVVVAAISEDLVKLVRVEK----PVSFKVRAVLTVRNKVKE  94
Ha_XP_022033566.1       -MALSKQLMGCSLTEKSQ----FLSSN--ILKSINYSSNQVSVSPKRCVRTR------VPVVKAAISEDLKDLAKFVN-AEKAVTFKVRAVLTVRNKYQE  86
Tc_TcLOX1               -MALAKQIMGAS-LMDQK--TSVFGSN--------LCLNHVLVN-KHRLRLRK-TRKNGSMVVAAISEDLVKLXRVEKEK--PVTFKVRAVLTVRNKNKE  84

Aa_PWA79444.1           D-FKETLVKKIDAFADQIGRNVVLELFSVEIDSKTRAPKKSKEGVLKDWSQKSNLKSEKVNYTSDILVESDFGVPGAITITNKHQKEFYLESITIEGFAC  188
Aa_PWA92286.1           D-FKDTIFRKIDALTDQIGWNVVIQLFSNDIDPKTRAPRKSNEAVLKDWSKKSNVKTERVNYTADIMVDSDFGIPGAITISNKHQKEFFLETITIEGFAC  182
Aa_PWA99065.1           D-FKETLVKKIDAFADQIGRNVVLELFSVEIDSKTRAPKKSKEGVLKDWSQKSNLKSEKVNYTSDILVESDFGVPGAITITNKHQKEFYLESITIEGFVC  188
Cs_Cse_sc001411.1_g060.1D-FKDTIFRKIDALTDQIGWNVVIQLFSNDIDPRTRAPKKSNEAVLKDWSKKSNVKTERVNYIADIMVDSDFGIPGAITISNKHQKEFFLETITIEGFAC  182
Cs_Cse_sc022182.1_g030.1D-FKETLVKKIDAFADQIGRNVVLELFSVEIDSKTRAPKKSKEGVLKDWSQKSNLKSEKVNYTSDILVESDFGVPGAITITNKHQKEFYLESITIEGFAC  187
Ha_XP_021984540.1       D-LTETIVRKLDAFADQIGRNVVLELHSNDIDPKTRAPKKSKEAALKDWSKKSNFKTERVNYTADILVDSEFGIPGAIAITNKHQKEFFLESIIIEGFAC  193
Ha_XP_022033566.1       D-FKETLVKKFDALADQIGRNVVLLVHSVDIDPKTRAPKKSKEAVLKDWSEKSNLKSEKVNYTCDILVGSDFGIPGAISISNKHQKEFYLESITIEGFAC  185
Tc_TcLOX1               DFFKDTIFRKIDAITDQIGWNVVIQLFSNDIDPRTRAAKKSNEAVLKDWSKKSNVKTERVNYTADIMVDSDFGIPGAITISNKHQKEFFLETITIEGFAC  184

Aa_PWA79444.1           GPVYFPCNSWVQSVNDHPEPRIFFSNQPYLPDQTPAGIKLLREKELRDLRGDGTGVRKLSDRIYDYDVYNDLGNPDRGNDFIRPLLGGQKIPYPRRCRTG  288
Aa_PWA92286.1           GPVHFPCNSWVQSIKDLPNPRIFFTNQPYLPDETPAGLKSLRYQELKDLRGDGTGVRKLSDRIYDYDVYNDLGNPDRGNDFVRPTLGGDKLPYPRRCRTG  282
Aa_PWA99065.1           GPVYFPCNSWVQSVNDHPEPRIFFSNQPYLPDQTPAGIKLLREKELRDLRGDGTGVRKLSDRIYDYDVYNDLGNPDRGNDFIRPLLGGQKIPYPRRCRTG  288
Cs_Cse_sc001411.1_g060.1GPVHFPCNSWVQSTKDLPNPRIFFTNQPYLPDETPEGLKSLRYQELKDLRGDGTGVRKLSDRIYDYDVYNDLGNPDRGNDFIRPTLGGEKLPYPRRCRTG  282
Cs_Cse_sc022182.1_g030.1GPVYFPCNSWVQSVNDHPEPRIFFSNQPYLPDQTPAGIKLLREKELRDLRGDGTGVRKLSDRIYDYDVYNDLGNPDRGNDFIRPLLGGQKIPYPRRCRTG  287
Ha_XP_021984540.1       GPVHFPCNSWVQSYKDYSKPRVFFSNQPYLPHETPAGLRYLRDQELKDIRGDGKGVRKLSDRIYDYDVYNDLGNPDRGNEFIRPSLGGDKMPYPRRCRTG  293
Ha_XP_022033566.1       GPVYFPCNSWVQSTNDHPNPRIFFSNQPYLPDQTPAGLKLLREKELTYLRGDGKGVRKLSDRIYDYDVYNDLGNPDRGSEFSRPILGGQKIPYPRRCRTG  285
Tc_TcLOX1               GPVHFPCNSWVQSTKDLPNPRIFFTNQPYLPDETPVGLKSLRYQELKDLRGDGTGVRKLSDRIYDYDVYNDLGNPDRGNDFVRPTLGGEKIPYPRRCRTG  284

Aa_PWA79444.1           RAPSDTDISAESRVEKPFPLYVPRDEQFDESKQNTFSTSRLKAVLHNLLPSMVASISKKHDFKGFSQIESLYSEGVLLKLGLQDDLIKKLRLPNLVTRLH  388
Aa_PWA92286.1           RVPSDTDITAESRVEKPFPMYVPRDEQFEESKANAFSTGRLRAVLHNLLPSMVTSISKKNDFKGFSQIDSLYSEGLFLKLGLQDDLLKKLPLPNLVTRLH  382
Aa_PWA99065.1           RTPSDTDISAESRVEKPFPLYVPRDEQFDESKQNTFSTSRLKAVLHNLLPSMVASISKKHDFKGFSQIESLYSEGVLLKLGLQDDLIKKLRLPNLVTRLH  388
Cs_Cse_sc001411.1_g060.1RVPSDTDITAESRIEKPFPMYVPRDEQFEESKANAFSTGRLRAVLHNLLPSMVTSISKKHDFKGFSQIDSLYSEGLFLKLGLQDDLLKKLPLPNLVTRLH  382
Cs_Cse_sc022182.1_g030.1RAPSDTDISAESRVEKPFPLYVPRDEQFDESKQNTFSTSRLKAVLHNLLPSMVASISKKHDFKGFSQIESLYSEGVLLKLGLQDDLIKKLRLPNLVTRLH  387
Ha_XP_021984540.1       RAPSDTDILAESRVEKPLPMYVPRDEQFEESKANAFSTGRLKAVLHNLLPSIVASISKKHDINGFSQLDSLYSKGVVLKLGLQDDLVKKLPLPKLVTRLH  393
Ha_XP_022033566.1       RLPSDTDILCESRVEKPFPLYVPRDERFEESKRITFSTSRLKAVLHNLLPSMVANISKKHDFKGFSQIESLYSEGVPLKFGLQDDLLKKLRLPNIVS---  382
Tc_TcLOX1               RVPSDTDITAESRVEKPFPLYVPRDEQFEESKANAFSTGRLRAVLHNLLPSMVTSISKKNDFKGFSQIDSLYSEGVFLKLGLQDDLLKKLPLPNLVTRLH  384

Aa_PWA79444.1           ESSQGGGLLKYDTPKILSKDRFSWLRDDEFARQALAGVNPVSIEKLKVFPPVSHLDPEIYGPQESALKEEHILGYLNGMTVQQAIEENKLFIIDYHDIYL  488
Aa_PWA92286.1           ESSQGGGLLKYDLPKILSKDKFAWLRDDEFARQTIAGVNPVSIEKLKVFPPMSQLDPDKYGPQESALREEHIVGYLDGMTVQQAIEENKLFIIDYHDIYL  482
Aa_PWA99065.1           ESSQGGGLLKYDTPKILSKDRFSWLRDDEFARQALAGVNPVSIEKLKVFPPVSQLDPEIYGPQESALKEEHILGYLNGMTVQQAIEENKLFIIDYHDIYL  488
Cs_Cse_sc001411.1_g060.1ESSQGGGLLKYDLPKILSKDKFAWLRDDEFARQTIAGVNPVSIEKLKVFPPVSQLDPDKYGPQESALREEHIAGYLDGMTVQQAIEENKLFIIDYHDIYL  482
Cs_Cse_sc022182.1_g030.1ESSQGGGLLKYDTPKILSKDRFSWLRDDEFARQALAGVNPVSIEKLKVFPPASQLDPEIYGPQESALKEEHILGYLNGMTVQQAIEENKLFIIDYHDIYL  487
Ha_XP_021984540.1       ESSQGGGLLKYDTPKILSKDKFAWFRDDEFARQTIAGVNPVSIERLQVFPPVSRLDPDKYGSQESALREEHIVGHLNGMSVQQALEENKLFIIDYHDIYL  493
Ha_XP_022033566.1       --SQGGGLLKFDIPKILSKDKFAWLRDDEFARQALAGVNPVNIEKLNVFPPVSQLDPEIYGLQESALKEEHISGYLNGLTVQQAMEENKLFIIDHHDVYL  480
Tc_TcLOX1               ESSQGGGLLKYDTPKILSKDKFAWLRDDEFARQTIAGVNPVSIEKLKVFPPVSQLDPEKHGPQESALREEHIVGFLDGRTVKQAIEEDKLFIIDYHDIYL  484

Aa_PWA79444.1           PFLDRINALDERKAYATRTIFFLTPSGTLKPIAIELSLPRALPGSQPKRVLTPPIDATSNWTWQLAKAHVCSNDAGVHQLSHHWLRTHAAMEPFILSAHR  588
Aa_PWA92286.1           PFLDRINALDGRKAYATRTIFYLNPSGTLKPVAIELSLPQALPGSESNRVLTPPSDATSNWMWQLAKAHVCSNDAGAHQLVHHFLRTHAATEPFILAAHR  582
Aa_PWA99065.1           PFLDRINALDERKAYATRTIFFLTPSGTLKPIAIELSLPRALPGSQPKRVLTPPIDATSNWTWQLAKAHVCSNDAGVHQLSHHWLRTHAAMEPFILSAHR  588
Cs_Cse_sc001411.1_g060.1PFLDRINALDGRKAYATRTVFYLNPSGTLKPVAIELSLPQALPGSESKRVLTPPSDATNSWLWQLAKAHVCANDAGAHQLVHHFLRTHAATEPFILAAHR  582
Cs_Cse_sc022182.1_g030.1PFLDRINALDERKAYATRTIFFLTPSGTLKPIAIELSLPRALPGSQPKRVLTPPVDATSNWTWQLAKAHVCSNDAGVHQLSHHWLRTHAAMEPFILSAHR  587
Ha_XP_021984540.1       PFLDRINALDGRKAYATRTIFFLNPSGTLMPVAIELSLPQALPGTESKRVVTPPADATSTWLWQLAKAHVCSNDAGVHQLVHHWLRTHASMEPFILAAHR  593
Ha_XP_022033566.1       PFLDRINALDGHKTYATRTIFYLNESGTLKPVAIELSLPR-----QSKRVVTPPIDATSNWTWLLAKAHVCSNDAGVHQLVNHWLRTHATMEPFILSAHR  575
Tc_TcLOX1               PFLDRINALDGRKAYATRTIFYLNPSGTLKPVAIELSLPQALPGSESKRVLTPPSDATSNWMWQLAKAHXCSNDAGAHQLVHHFLRTHAAIEPFILAAHR  584

Aa_PWA79444.1           QLSAMHPIYKLLDPHMRYTLEINAIARQNLLNADGVIEQGFTPGRYCMEISAAAYKHWRFDLEGLPADLIRRGMAVPDPSQRHGLKLLIEDYPYASDGLL  688
Aa_PWA92286.1           QLSAMHPIYKLLDPHMRYTLEINQLARQNLINADGVIEQCFTPGRYCMEISAAAYKNWRFDLEGLPADLIRRGMAIPDPSKPHGLKLVIEDYPYASDGLM  682
Aa_PWA99065.1           QLSAMHPIYKLLDPHMRYTLEINAIARQNLLNADGVIEQGFTPGRYCMEISAAAYKHWRFDLEGLPADLIRRGMAVPDPSQRHGLKLLIEDYPYASDGLL  688
Cs_Cse_sc001411.1_g060.1QLSAMHPIYKLLDPHMRYTLEINQLARQNLINADGVIEACFTPGRYCMEISANAYKNWRFDLEGLPADLIRRGMAVPDPSKPHGLKLVIEDYPYASDGLM  682
Cs_Cse_sc022182.1_g030.1QLSAMHPIYKLLDPHMRYTLEINAIARQNLLNADGVIEQGFTPGRYCMEISAAAYKHWRFDLEGLPADLIRRGMAVPDPSQRHGLKLLIEDYPYASDGLL  687
Ha_XP_021984540.1       QLSAMHPIYKLLDPHMRYTLEINALARQNLINADGVIEQCFTPGRYCMEMSAAAYKNWRFDLEGLPADLIRRGVAVRDPSKPHGLKLLIEDYPYASDGLM  693
Ha_XP_022033566.1       QLSAMHPIFKLLDPHMRYTLETNAIARQNLINADGVIEQGFTPGRYAMEISAAAYKNWRFDLEGLPADLIRRGMAIPDPSQRHGLKLLIEDYPYASDGLL  675
Tc_TcLOX1               QLSAMHPIYKLLDPHMRYTLEINQLARQNLINADGVIEACFTPGRYGMEISASAYKNWRFDLEGLPADLIRRGMAVPDPSKPHGLKLVMEDYPYASDGLM  684

Aa_PWA79444.1           IWEAIQDWVRTYVNRYYPDPSLVCNDRELQAWYAEVINVGHADLRYENWWPTLANAEDLTAILTTIIWLASAQHAALNFGQYPYGGYIPNRPPRMRRLIP  788
Aa_PWA92286.1           IWEAIQNWVKTYVNHYYPDSAQVCNDRELQAWYAESINVGHADLRHKDWWPTLASADDLTSVLTTLIWLASAQHAALNFGQYPYGGYIPNRPPLMRRLLP  782
Aa_PWA99065.1           IWEAIQDWVRTYVNRYYPDPSLVCNDRELQAWYAEVINVGHADLRYENWWPTLANAEDLTAILTTIIWLASAQHAALNFGQYPYGGYIPNRPPRMRRLIP  788
Cs_Cse_sc001411.1_g060.1IWEAIQNWVKTYVNHYYPDSAHVCNDRELQAWYAESINVGHADLRHKDWWPTLASADDLSSVLTTIIWLASAQHAALNFGQYPYGGYIPNRPPLMRRLLP  782
Cs_Cse_sc022182.1_g030.1IWEAIQDWVRTYVNRYYPDPSLVCNDRELQAWYAEVINVGHADLRHENWWPTLANAEDLTAILTIIIWLASAQHAALNFGQYPYGGYIPNRPPRMRRLIP  787
Ha_XP_021984540.1       IWEAIQTWVRTYVNHYYPDSAHVCNDRELQAWYAESINVGHADLRHEDWWPTLADADDLTGILTTIIWLASAQHAALNFGQYPYGGYIPNRPPLMRRLLP  793
Ha_XP_022033566.1       IWEAIQNWVRTYVTQYYPDASFVCNDSELQAWYAESINVGHAELRNETWWPTLANVEDLTAILTTVIWLASAQHAALNFGQYPYSGYIPNRPPLMRRLIP  775
Tc_TcLOX1               IWEAIQNWVKTYVNHYYPDSAQVCNDRELQAWYAESINVGHADLRHKDWWPTLAGADDLTSVLTTIIWLASAQHAALNFGQYPYGGYIPNRPPLMRRLLP  784

Aa_PWA79444.1           DENDPEYASFLEDPQNYFLLALPSMLQSTKYMAVVDTLSTHSPDEEYIGERQQRDTWSGDAEMVEAFYGFASEIQRIEKEIEKRNRDMSLKNRCGAGVLP  888
Aa_PWA92286.1           DQNDPEYLSFHDDPQKYFLSALPSLLQSTKYMAVVDTLSTHSPDEEYIGERQQTDTWSGDAEIVEAFYSFSAEIQRIEKEIEKRNSDTSLKNRCGAGVLP  882
Aa_PWA99065.1           DENDPEYTSFLEDPQNYFLLALPSMLQSTKYMAVVDTLSTHSPDEEYIGERQQRDTWSGDAEMVEAFYGFASEIQRIEKEIEKRNRDMSLKNRCGAGVLP  888
Cs_Cse_sc001411.1_g060.1DQNDPEYLSFHDDPQKYFLSALPSLLQSTKYMAVVDTLSTHSPDEEYIGERQQTDTWSGDAEIVEAFYAFSAEIQRIEKEIEKRNSDTSLKNRCGAGVLP  882
Cs_Cse_sc022182.1_g030.1DENDPEYTSFLEDPQNYFLLALPSMLQSTKYMAVVDTLSTHSPDEEYIGERQQRDTWSGDAEMVEAFYGFASEIQRIEKEIEKRNRDMSLKNRCGAGVLP  887
Ha_XP_021984540.1       DENDPEYMSFHDDPQKYFLSALPSLLQSTKYMAVVDTLSTHSPDEEYIGDRQQTDTWTGDSEIVEAFYKFSAEIQRIEKEIEKRNRDPSLKNRCGAGVLP  893
Ha_XP_022033566.1       DEMDPEYASFLEDPQNYFLSAIPSMLQSTKFMAVVDTLSTHSPDEEYIGQRQQTDTWTGDAEMVEAFYMFSTEIKRIEQEIERRNRDTSLKNRCGAGVLP  875
Tc_TcLOX1               DVNDPEYLSFHDDPQKYFLSALPSLLQSTKYMAVVDTLSTHSPDEEYIGERQQTDTWSGDAEIVEAFYAFSAEIQRIEKEIEKRNSDTSLKNRCGAGVLP  884

Aa_PWA79444.1           YELLAPSSEPGVTCRGIPNSVSI----------------------------------------------------------------------
Aa_PWA92286.1           YELLAPSSEPGATCRGVPNSISI----------------------------------------------------------------------
Aa_PWA99065.1           YELLAPSSEPGVTCRGIPNSVSI----------------------------------------------------------------------
Cs_Cse_sc001411.1_g060.1YELLAPSSEPGATCRGVPNTINIRRSKDYKPSIPLPPMSYNLQASHTKPLYKELCGNLTIGEVATCLRQQGDEVFLMSHGDKVVKVPNALRLS      975
Cs_Cse_sc022182.1_g030.1YELLAPSSEPGVTCRGIPNSVSI----------------------------------------------------------------------
Ha_XP_021984540.1       YELLAPSSESGATCRGVPNSVSI----------------------------------------------------------------------
Ha_XP_022033566.1       YELLAPSSQPGVTCRGVPNSVSI----------------------------------------------------------------------
Tc_TcLOX1               YELLAPSSGPGATCRGVPNSISI----------------------------------------------------------------------
```

Supplemental Figure 4. Molecular phylogenetic analysis of *T. cinerariifolium* 13-lipoxygenase (TcLOX1). TcLOX1-related proteins were searched by BLASTP against 14 different plant protein sets and incorporated into maximum likelihood tree analysis (A) using ORTHOSCOPE method[56]. Proteins incorporated into this tree include 9 from *T. cinerariifolium*, 10 from *C seticuspe*, 10 from *A. annua*, 9 from *H. annuus*, 10 from *Z. mays*, 10 from *V. vinifera*, 10 from *M. truncatula*, 10 from *S. lycopersicum*, 10 from *P. trichocarpa*, 10 from *O. sativa*, 9 from *G. raimondii*, 10 from *N. tabacum*, 10 from *G. max*, and 7 from *A. thaliana*. TcLOX1 nodes are indicated in blue; *T. cineariifolium* nodes and edges are indicated in red; other Asteraceae nodes and edges are indicated in orange. The amino acid sequence alignments for maximum likelihood tree analysis are shown in panel B.

Supplemental Figure 5.

A

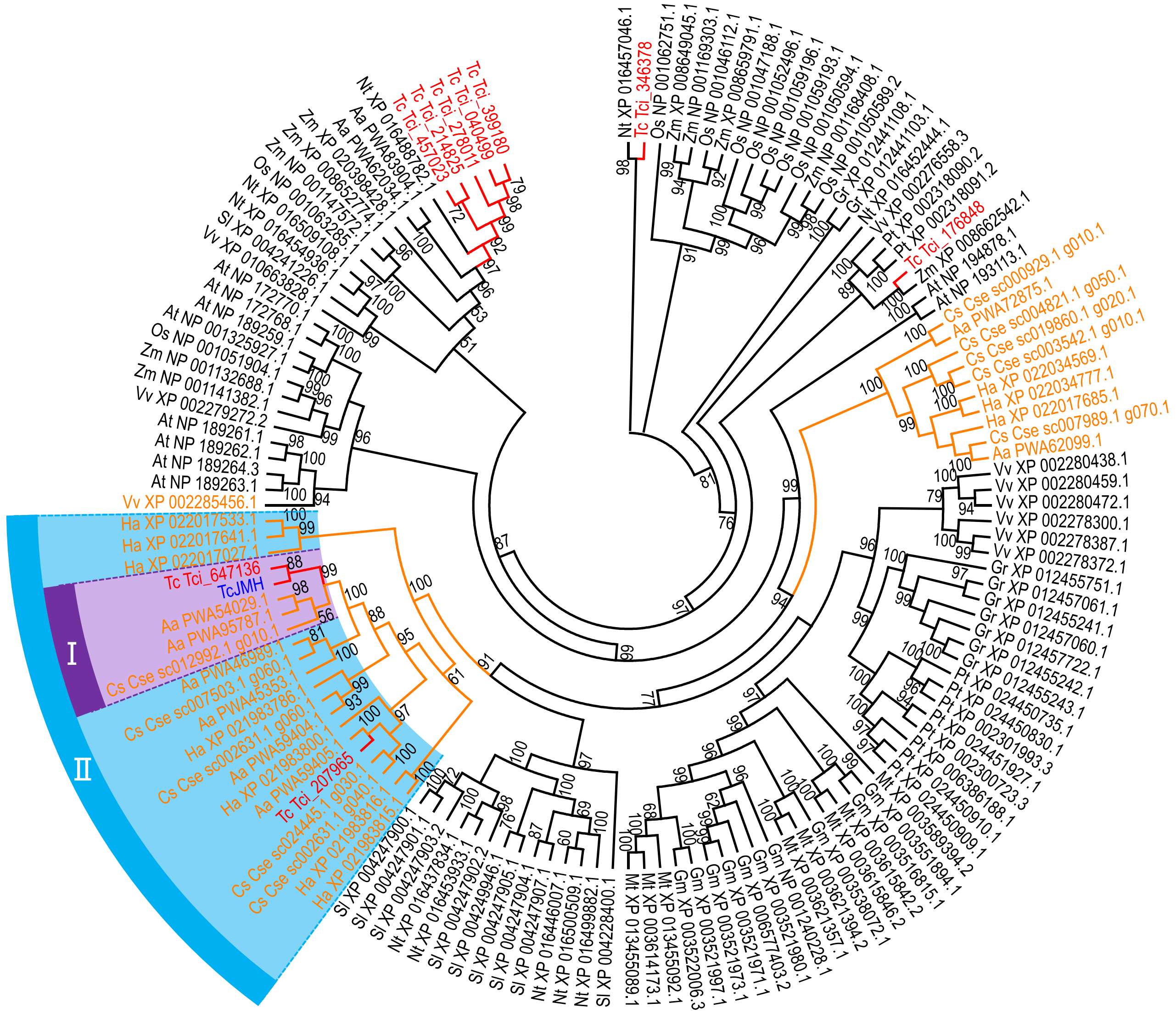

B

```
Aa_PWA45353.1           --------------------------------------MISIMALLFLYIFLLFPILSLLY-IVPKIIK-NKSRIN------PPGPLGLPFIGNLHQIDS  54
Aa_PWA46989.1           ----------------------------------------------------------------------------------------------------
Aa_PWA54029.1           ------------------------------------------MALFFMYIFLLFPILYLIY-LLPKINKNRSSRLA------PPGPLGLPFIGNLHQIDS  51
Aa_PWA59404.1           ------------------------------------------MDSLLQFLLASLPILYLLYQLIPKIIKNKSKSNVHGQFRSPPGPHGMPFIGNLHQIDK  58
Aa_PWA59405.1           ------------------------------------------MALLFLFLLMSLPLLSLLY-LFPKFIKNKSQFDP-------PGPRGLPFIGNLHQIDQ  50
Aa_PWA95787.1           ------------------------------------------MALFFMYIFLLFPILYLIY-LLPKINKNRSSRLA------PPGPLGLPFIGNLHQIDS  51
Cs_Cse_sc002631.1_g040.1----------------------------------------------------------------------------------------------------
Cs_Cse_sc002631.1_g060.1------------------------------------------MDS-LQFLLASLPILFLLY-LLPKIIKSKSKSSVQGQFRSPPGPHGLPFIGNLHQIDK  56
Cs_Cse_sc007503.1_g060.1------------------------------------------MALFSLCIFLLFPILSLFY-IVPKIIK-NKSRIN------PPGPLGLPFIGNLHQIDS  50
Cs_Cse_sc012992.1_g010.1------------------------------------------MALFFMYIFLLFPILYLIY-LLPKISK-RSSRLA------PPGPLGLPFIGNLHQIDS  50
Cs_Cse_sc024445.1_g030.1------------------------------------------MALLFLFLLASLPLLSVLY-LFPKIIKNKSQFDP-------PGPRGLPIIGNLHQIDQ  50
Ha_XP_021983786.1       ------------------------------------------MDSLIQFLLVSLLFLSFIY-LLPKIVKTKSKTSR------PPGPRGLPFIGNMHQIDN  51
Ha_XP_021983800.1       --------------------------------------------MSLLLLLFLPLLSLIY-LLPKIIILNKSRFNP-------PGPLGLPLIGNLHQIEQ  48
Ha_XP_021983815.1       ------------------------------------------MELVFMFILLSLLLLSLIY-LLPKIIKNKPKFSP-------PGPPGLPFIGNLHQINQ  50
Ha_XP_021983816.1       ------------------------------------------MELLFMFLLFS---LSLMY-LLPKIIKHKSRFNP-------PGPIGLPFIGNLHQINP  47
Ha_XP_022017027.1       ------------------------------------------MISNMLLPFLFLLLLSLLY-LLH---KRKSKFNP-------PGPPGLPFIGNLHQLDQ  47
Ha_XP_022017533.1       --------------------------------------MLRTMISNMLLPFLFLFLSTLLY-ILP---KKKSKFNP-------PGPPGLPFIGNLHQLDQ  51
Ha_XP_022017641.1       --------------------------------------MLRTMISNMLLPFLFLFLWTLLY-ILP---KKKSKFNP-------PGPPGLPFIGNLHQLDQ  51
Tc_TcJMH                -------------------------------------MMIPTMASVFVYILLPFPILYLIN-LVWKINKNRLNRLN------PPGPLGLPFIGNLHQIDS  56
Tc_Tci_207965           MSNEQKYPGKFLRIQRRKPQMPARLLVFFAFQKEVEFRNVSTMALLFLFLLMSLPLLSLLY-LLPKIIKNKSQFDP-------PGPRGLPFIGNLHQIDQ  92
Tc_Tci_647136           ----------------------------------------------------------------------------------------------------

Aa_PWA45353.1           SSIHTSLWNLSRSYGPIVYLSFGFVSCIVVSSASLAKEVLKTQDLIFCSRPSMVAQRK------------------------------------------  112
Aa_PWA46989.1           ----------------------------------------------------------------------------------------------------
Aa_PWA54029.1           SSLHTSLWNLSKSYGPILFLRFGFIPSIVVSSASLAKEVLKTQDVIFCSRPSSVSQRK------------------------------------------  109
Aa_PWA59404.1           SNFHISLWSLSKSYGPVVSLNLGFIPAIVISSASVAKEILKTQDLTFCSRPSFHGLQR------------------------------------------  116
Aa_PWA59405.1           SHLHTYLWNLSKSYGPVLSLQFGFIPAITVSSASLAKEVLKTQDIIFCSRPTLFGQQK------------------------------------------  108
Aa_PWA95787.1           SSLHTSLWNLSKSYGPILFLRFGFIPSIVVSSASLAKEVLKTQDVIFCSRPSSVSQRK------------------------------------------  109
Cs_Cse_sc002631.1_g040.1----------------------------------------------------------------------------------------------------
Cs_Cse_sc002631.1_g060.1SNLHISMWSLSKSYGPVVSLNLGFIPAIVVSSASVAKEILKTQDITFCSRPSFHGLQRVSYNGIDVALSPYNKNWKEMRRIFTVYLFSPKRLQYSRFI||  156
Cs_Cse_sc007503.1_g060.1SSIHTSLWNLSRSYGPILYLNFGFVPCIVVSSASLAKEVLKTQDLIFCIR--------------------------------------------------
Cs_Cse_sc012992.1_g010.1SSLHTSLWNLSKSYGPILFLRFGFIPSIIVSSASLAKEVLKTQDVIFCSRPSSVSQRK------------------------------------------  108
Cs_Cse_sc024445.1_g030.1LNLHTSLWNISKTYGPIVSLQFGFIPTIVVSSASLAKQVLKTQDSVFCNRPSLHGQQK------------------------------------------  108
Ha_XP_021983786.1       SSLHTSLWKLSKSYGPIVSLNLGFIPAIVVSSASLAKEILKTQDHTFCSRPLLHGIKK------------------------------------------  109
Ha_XP_021983800.1       SSLHTSLWNLSKSYGPIVSLRFGFIPVIVVSSASLAKEVMKTQDLIFCSRPSLVGQQK------------------------------------------  106
Ha_XP_021983815.1       STLHTSLWQLSKSYGPIISLRFGFVPAIVVSSASLAKEVMKTQDIIFCSRPRLVGNQK------------------------------------------  108
Ha_XP_021983816.1       SSLHTSLWQLSKSYGPIISLRFGFVPTIVVSSPSLAKEVMKTQDIIFCSRPRLVGNQK------------------------------------------  105
Ha_XP_022017027.1       SSLHTSLWNLTKPYGPIISLRFGFNSAVVISSASLAKEVLKTQDLIFCGRPLFTGQRK------------------------------------------  105
Ha_XP_022017533.1       SSLHTSLWNLTKSYGPIVSLRFGFNSAVVISSASLAKEVLKTQDLSFCDRPSFIGQQK------------------------------------------  109
Ha_XP_022017641.1       SSLHTSLWNLTKPYGPIVSLRFGFNSAVVISSASLAKDVLKTQDLSFCDRPSFIGQQK------------------------------------------  109
Tc_TcJMH                SSLHTCLLNLSKSYGPILFLRFGSIPSIVVSSASLAKEVYKTQDVIFSSRPCFVSHRK------------------------------------------  114
Tc_Tci_207965           SHLHTYLWNLSKSYGPVVSLQFGFIPAIAVSSASLAKEVLKTQDIIFCNRPTLVGQQK------------------------------------------  150
Tc_Tci_647136           -----------------------MLPEKLSSP--------------------------------------------------------------------

Aa_PWA45353.1           ----------------------------------------------------------------------------------------------------
Aa_PWA46989.1           ----------------------------------------------------------------------------------------------------
Aa_PWA54029.1           ----------------------------------------------------------------------------------------------------
Aa_PWA59404.1           -------------------------------------------------------------------SLPILYLLYLIPKIIKNKSKSNVHGQFRSPPGP  149
Aa_PWA59405.1           ----------------------------------------------------------------------------------------------------
Aa_PWA95787.1           ----------------------------------------------------------------------------------------------------
Cs_Cse_sc002631.1_g040.1----------------------------------------------------------------------------------------------------
Cs_Cse_sc002631.1_g060.1MGVNSVELFLANLIYSFDWGLPDGTKIEDIDSGQLSCWFFKKSCFALQKVEFRNVSTMALPFLFLLMSLPLLSLLYLLPKIIKSKPQS-------VPPGP  549
Cs_Cse_sc007503.1_g060.1----------------------------------------------------------------------------------------------------
Cs_Cse_sc012992.1_g010.1----------------------------------------------------------------------------------------------------
Cs_Cse_sc024445.1_g030.1----------------------------------------------------------------------------------------------------
Ha_XP_021983786.1       ----------------------------------------------------------------------------------------------------
Ha_XP_021983800.1       ----------------------------------------------------------------------------------------------------
Ha_XP_021983815.1       ----------------------------------------------------------------------------------------------------
Ha_XP_021983816.1       ----------------------------------------------------------------------------------------------------
Ha_XP_022017027.1       ----------------------------------------------------------------------------------------------------
Ha_XP_022017533.1       ----------------------------------------------------------------------------------------------------
Ha_XP_022017641.1       ----------------------------------------------------------------------------------------------------
Tc_TcJMH                ----------------------------------------------------------------------------------------------------
Tc_Tci_207965           ----------------------------------------------------------------------------------------------------
Tc_Tci_647136           ----------------------------------------------------------------------------------------------------

Aa_PWA45353.1           -----------LLYNCIDVVFSSYNEYW----------------------RDMRKIFTIHLSSPKRVL--------------------------------  147
Aa_PWA46989.1           ----------------------------------------------------------------------------------------------------
Aa_PWA54029.1           -----------FSYNGLDMVFSPYNDYW----------------------RDMRKIVAIHLLSSKRVQ--------------------------------  144
Aa_PWA59404.1           HGMPFIGNLHQIDKSNFHISLWSLSKSYGPVVSLNLGFIPAIVVSSASVAKEILKTQDLTFCSRPSFHGLQ-----------------------------  220
Aa_PWA59405.1           -----------ISYNGLEVIFSPYNEYY----------------------REMRKIFMVHLLGPKRVQ--------------------------------  143
Aa_PWA95787.1           -----------FSYNGLDMVFSPYNDYW----------------------RDMRKIVAIHLLSSKRVQ--------------------------------  144
Cs_Cse_sc002631.1_g040.1---------------------------------------------------------MAHLLGPKRVQ--------------------------------  11
Cs_Cse_sc002631.1_g060.1HGLPFIGNLHQIDQLHLHTYLWNLSKLYGPVVSLQFGFIPAIVVSSASLAKEVLKTQDIIFCSRPTLFGQQKISYNGVELIFSPYNEYYREMRKIFMVHL  649
Cs_Cse_sc007503.1_g060.1----------------------------------------------------------------------------------------------------
Cs_Cse_sc012992.1_g010.1-----------LTYNGLDVAFSPYNDYW----------------------RDMRKIVNIHLLSSKRVQ--------------------------------  143
Cs_Cse_sc024445.1_g030.1-----------LSYNGLDVAFSPYNEYW----------------------REMRNFFMTHLLGRKRVQ--------------------------------  143
Ha_XP_021983786.1       -----------IGYNGHDVALSPYNRNW----------------------KEMRKIFSVYLFSPKRIQ--------------------------------  144
Ha_XP_021983800.1       -----------VSYHGLEVIFSPYNDYW----------------------KEMRKIFMLHLLGPKRVQ--------------------------------  141
Ha_XP_021983815.1       -----------FSYGGLDVAFSSYDETW----------------------RDMRKIFVTHLLSPKRIQ--------------------------------  143
Ha_XP_021983816.1       -----------FSYGGLDVAFSPYDETW----------------------RDMRKIFVTHLLSPKRIQ--------------------------------  140
Ha_XP_022017027.1       -----------LSYEGLDITFAPYNEHW----------------------REMRKIFSLHLFSPKRVQ--------------------------------  140
Ha_XP_022017533.1       -----------LTYDGLDMAFSPYNEHW----------------------RKMRKIFTLHLFSPKRVH--------------------------------  144
Ha_XP_022017641.1       -----------LTYDGLDMAFSPYNEHW----------------------RKMRKIFSLHLFSPKRVH--------------------------------  144
Tc_TcJMH                -----------FSYNGLDVVFSPSNQYW----------------------RDMRKIVTIHLLSSKRVQ--------------------------------  149
Tc_Tci_207965           -----------VSYNGLEVIFSPYNEYN----------------------REMRKIFMVHLLGPKRVQ--------------------------------  185
Tc_Tci_647136           ---------------------------------------------------------VLLFLVVKAIN--------------------------------  20
```

```
Aa_PWA45353.1           -------ASRYIREDEISHAMKKINGLALSSENVNLSAIMKNVTSTIVMRVGFGKRYE--DGHERREILRLIDELQSMITNFFVEDLWPGLPFAGLIDKL  238
Aa_PWA46989.1           ---------------------------------------MKNVTSTIVMRVGFGKRYE--DGHKRREILRLIDELQSMITNFFVEDLWPGLPFAGLIDKL  59
Aa_PWA54029.1           -------SSRYIREEEVLHAMKKIHNLSLSSKHINLTEIMINVTSTIVMRVGFGKRYE--DGHERREIVRLIGELQAMITDFFVADLWPGLPFASLIDRL  235
Aa_PWA59404.1           ------RSSRFIREDEVSLAMEKIHGLALSSKHVNLSEIAHIVMSNMVTRIGFGKRYE--DGYESKEILRLLHELQATLTNYFISDLWPDFPLVGLIDRL  312
Aa_PWA59405.1           -------SYRYIREEEVSSAMKTIFGLALTSKQVNLSEITKSVASTIVMRVGFGKRYQ--DGHERKEVLRLLLEVQAMMANFFVSDLWPGLPFAGLADRL  234
Aa_PWA95787.1           -------SSRYIREEEVLHAMKKIHNLSLSSKHINLTEIMINVTSTIVMRVGFGKRYE--DGHERREIVRLIGELQAMITDFFVADLWPGLPFASLIDRL  235
Cs_Cse_sc002631.1_g040.1-------SFSYIREDEVSNAMKTIHGLALSSKKVNLSEITKRVTSNIMMRVGFGKRYQ--DGHESKEVLRQLTELQAIMANFFASDLWPGLPFVDWIDKL  102
Cs_Cse_sc002631.1_g060.1LGPKRVQSYRYIREDEVSNAMKTIHGLALSAKQVNLSEITKSVASTIAMRVGFGKRYQ--EGHERKQVLRLLLEVQAMMANFFVSDLWPGLPFASLVDRL  747
Cs_Cse_sc007503.1_g060.1---------KYIREDEISHAMKKINELALLSKNVNLSEIMKNVTSTIIMRVGFGKRYK--DGHEHREILRLIDELQSMITNFFVEDLWPGLPFAGLIDKL  189
Cs_Cse_sc012992.1_g010.1-------SSMYIREDEVLHAMKRIHQLALSSKHVNLSEIMINVTSTIVMRVGFGKRYE--DGHERREIVRLLGELQSMITDFFVADLWPGLPFASLIDRL  234
Cs_Cse_sc024445.1_g030.1-------CFSYIHEDEVSNAMKIIHGLALSSKKVNLSEITKRVTSTIMTRVGFGKSYQ--DGHESKEVLRQLTELQAMIANFFASDLWPGLPFVGWIDKL  234
Ha_XP_021983786.1       -------SSRYIREDEVSRAMKKIHGLALSSNHVNLSVISHIVMSTIVTRVGFGKRYE--EGDKSKEILRLLHELQATITNYFLSDLWPGSKLAGLIDRM  235
Ha_XP_021983800.1       -------SFRHIREDEVTTAMKKIHELALSSKEVNLSEMMKSVASTIMMRVGFGKTYQ--DGHDRTEVLHRLTEVQAMMADFFASDLWPGLPFVGLVDRL  232
Ha_XP_021983815.1       -------STRDIREDEVSHAMSKIHGLALSSKHVNLSEITKSVTSTIMMRIGFGKRYQ--DENERKKVLGLLNELQETIVDNYVSDIWPGLPFVNLVDRF  234
Ha_XP_021983816.1       -------STRYIREDEVSHAMSKIHGLTLSSKHVNLSEITKSVTSTIMMRVGFGKRFQ--DENERKKVLGLLDELQETIVDNYVSDIWPGLPFVNLVDRF  231
Ha_XP_022017027.1       -------SLRYIQEGEISSAIKKIHDLALSAEKVNLSKMMKNLMSNVMMRVCFGKRYHYQNGHERKKVLGLINEVQEHLVDLYLSDIWPGLPFIGLVDRL  233
Ha_XP_022017533.1       -------SFRHIREDEVSTAMKTIHDLALSGKTVNLSEVMKNMTTIIMMKMCFGKKYHCRDGLEIKKVLGLLNEVQAYLVDLYFSDIWPGLPFVGLVDRL  237
Ha_XP_022017641.1       -------SFRHIREDEVSTAMKTIHDLALSGETVNLSEMMKNMTTIIMMKMCFGKKYHCRDGLEIKKVLGLLNEVQAYLVDLYFSDIWPGLPFVGLVDRL  237
Tc_TcJMH                -------SSRYMREEEVSLAMNKIHKLALSSEHINLTELMMNVTTTVVMRVGFGKRYE--DGHERTEIVRLLGELQSMLADFFVADLWPGLPFASLIDRL  240
Tc_Tci_207965           -------SYRYIREDEVSRAMKTIHGLALSSKQVNLSEITKSVASTIVMRVGFGKRYQ--DGHERKEVLRLLLEVQAMMANFFVSDLWPGLPFVDLVDRL  276
Tc_Tci_647136           -------S---LIKDEVALAMKKVHKLALSSKHINLTELMMNVTSTIVMRVGFGKRYE--DGHERREIVRLLGELQSMITDFFVADLWPGLPFASFIDRL  108

Aa_PWA45353.1           TGKIDRLEKCFQDLDSFYQELIDERLN-AQNAKSCE-EDQDILDILIQLKKEQLLNSTEFTNNHIKAMLT-------------------VIIQSISHENY  317
Aa_PWA46989.1           TGKIDRLEKCFQDLDSFYQELIDERLN-AQNAKSCK-EDQDILDILIQLKKEQLLNSTEFTNNHIKAMLT-------------------DVLVAGTDASA  138
Aa_PWA54029.1           TGKTDRLEKCFQDLDSFYQRLIDEHLVNDENTNSHDQEDQDIIDILIQLKKDQVSNPIELTNNHIKAMLT-------------------GF---------  307
Aa_PWA59404.1           LGKFYRLEKCLQGLDSFYQNLIDEHLD-TEYSKPNE-EHEDLIDILLQLRNGQLSDSFELTNDHMKAMLT-------------------DILVAGTDNSA  391
Aa_PWA59405.1           LGKTDRLERCFQYFDLFYQKLIDEHIN-RNDK-TSHEEEEDFIDILLRLKKDQL----------------------------------------------  286
Aa_PWA95787.1           TGKTDRLEKCFQDLDSFYQRLIDEHLVNDENTNSHDQEDQDIIDILIQLKKDQVSNPIELTNNHIKAMLT-------------------DVLVAGTDASA  316
Cs_Cse_sc002631.1_g040.1SGKSDRLEKCFQYFDLFYQDLIDEHTH-PIKFSKCEENEEYFLDILLRLRKDQHLN---LTYDHIKANLMVVFVGPLVSSLTVFFCLFKNVLAAGTDTSA  198
Cs_Cse_sc002631.1_g060.1LGKTNRLDRCFQYFDLFYQKLIDEHIN-RKDQTSHE-QEEDFIDILLRLKKDQL---FNLTNDHIKAMLM-------------------DVLVAGTDTIS  823
Cs_Cse_sc007503.1_g060.1TGKIDRLEKCFQDFDSFYQELIDERLN-AQNAKSCE-EDQDILDILIQLKEEQLLNSTEFTNNHIKAMLT-------------------DVLVAGTDASA  268
Cs_Cse_sc012992.1_g010.1MGKTDRLEKCFQDLDSFYQSLIDERLTNDENTNSHNQEDQDIIDILIQLKKDQVSNPIELTNNHIKAMLT-------------------DVLVAGTDASA  315
Cs_Cse_sc024445.1_g030.1SGKSDRLEKCFQYFDLFYQDLIDEHAH-PIKFSKCEENEEYFLDILLRLKKDQHLN---LTYDHIKANLM-------------------NVLAAGTDTSA  311
Ha_XP_021983786.1       MGKFYRLEKCFKDLDSFYQELIDEHLH-PQNPKSSE-GEHDLIDILLQLKKDHVLDPFELTNDHIKAIIT-------------------DILVAGTDNSA  314
Ha_XP_021983800.1       SGKTKRLDECFRYFDLFYQSLIDEHLK-PENT-KSREGEEDFVDILLRLKQDQLFN---LTHDHIKAMLM-------------------DVLVAGTDTSS  308
Ha_XP_021983815.1       MGKTDRLEKCFQDFDLFYQQLIDEHLN-GRNI-KSHEDEDDVIDILLRLMEDKLFS---LTHNHIKALLM-------------------NVLSAGTDPNA  310
Ha_XP_021983816.1       IGKTDRLEKCFQEFDLFYQQLIDEHLN-GRNI-RSHEDEDDVVDILLRLMKDKLFG---LTHKHIKAMLM-------------------NVLAAGTDASA  307
Ha_XP_022017027.1       MGKTNRLEKCFQDLDFFYQQLIDERLN-PLKDKSYEEDEDDVIDILLQLMKDKLFN---LTHDHIKAMLM-------------------DVLVAGTDTSA  310
Ha_XP_022017533.1       TGKMNRQDKCFRSLDSFYQQLIDERLN-PQEDKSYEEEEDDLIDILLQLEKDKLFS---LTHDHIKVMLM-------------------DVLVAGTDTSA  314
Ha_XP_022017641.1       TGKMNQQDKCFRSLDSFYQQLINERLN-PQEDKSYEEEEDDLIDILLQLEKDKLFS---LTHNHIKVMLM-------------------DVLVAGTDTSA  314
Tc_TcJMH                IGKTARLENCFRDLDLFYQSLIDERLN-AQNTNSHDQEDKNIMDILLHLKKDQVSSPIKLTDNHIKAMLT-------------------DVLAAGSDTSA  320
Tc_Tci_207965           LGKTDRLDRCFQYFDLFYQKLIDEHIN-RKDQ-KTHEEEEDFIDILLRLKKKQLFN---LTNDHIKAMLM-------------------DVLVAGTDTSS  352
Tc_Tci_647136           TGKTNRLEKCFQDLDSFYQSLIDERLS-AQNAKSHNQEDQDIMDILLQLKKDQVSNPIELTNNHIKAMLT-------------------DVLVAGTDATA  188

Aa_PWA45353.1           KFIFF-----------------------------------------------------------------------------------------------
Aa_PWA46989.1           ATVVWAMTALIKNPKVMKKVQEEVRNVVGKKK---CGINEDELPKLIYLKAIVKEIMRLYPPAPLLVPRVTKKDAILQGYKIKEKTLVYVNALAIGRDPE  235
Aa_PWA54029.1           ----------------------------------------------------------------------------------------------------
Aa_PWA59404.1           ATLVWAMTTLVKYPKAMKKAQEEVRKMVQNK----DKVDEDDLPKLTYLKAVVKEVMRLYPAAPLLIPRVTMKDAILLDYKIKQNTLVYVNAMAIGRDPE  487
Aa_PWA59405.1           ----------------------------------------------------------------------------------------------------
Aa_PWA95787.1           ATVVWAMTALIKNPKVMRKAQEEVRNVAGKK----GAIDENDLAQLIYLKAIVKEIMRLYPPAPLLVPRETKKDTILHGYEIKQKTLVHVNAFAIGRDPE  412
Cs_Cse_sc002631.1_g040.1ATVVWAMTTLMKYPKVMENAQ-EVRNVVGK----KGKADEDDLPKLTYMKVAVKETMRLYPTVPILLPRETTKETILHGYKIKPKTLG---------DSE  284
Cs_Cse_sc002631.1_g060.1ATVVWAMAALIKNPEVMKKVQEEVRHVVGKK----DKVDEADLPKLAYMKAVVKEIMRLYPTVPLLVPRETTKETILHGYRIKPKTLVFVNALAIGRDPE  919
Cs_Cse_sc007503.1_g060.1ATVVWAMTALIKNPKVMKKVQEEVGNVVGKKK---CEINEDELPKLIYLKAIVKEILRLYPPAPLLVPRVTKKDTTLQGYKIKEKTLVHVNALAIGRDPE  365
Cs_Cse_sc012992.1_g010.1ATVVWAMTALMKNPKVMRKAQEEVRNVVGKK----GAIDENDLAQLIYLKAIVKEIMRLYPPAPLLVPRETKKDTILHGYEIKQKTLVHVNAFAIGRDPE  411
Cs_Cse_sc024445.1_g030.1ATVVWAMTALMKYPQVMKNAQEEVRNVVGK----KGRVDKDDLPKLTYMKAIVKETMRLYPSVPLLLPRETTKETILHGYKIKPKTLVFVNALAIGRDPE  407
Ha_XP_021983786.1       ATLVWAMTSLIKNPKEMKKVQEEVRNAVGDK----GKVDEDDLSKLTYMKAVVKEVLRLYPAAPLLIPRETTEDTIIHGYKIKKKTIVYVNALAIGRDPG  410
Ha_XP_021983800.1       ATVVWAMTALIKNPKVMKKVQEEVRNVVGK----KSKVDEDDLPKLIYMKAVVKEIWRLYPTVPLLVPRETTKETTLNGYKIKQKTLVFINTLAIGRDPK  404
Ha_XP_021983815.1       ASVVWSMTLLIKNPEVMKKAQEEVRNMIGK----KGKIDEDDLPKLTYLKAVIKETMRLYPPAPLLLPREAMKDAVLHCYKIKQKTIVYVNAYAIGRDPE  406
Ha_XP_021983816.1       ATVVWSLTLLMKNPNVMKKAQEEVRNVIGK----KCMIDEDDLPKLTYLKAVINETMRLYPSAPLLVPRETMKDTILHGYKIKQKTIVYVNAFAIGRDPK  403
Ha_XP_022017027.1       ATVVWAMTSLIKNPKIMKKTQEEMRVVIGK----KGIIEEDDLPKLMYLKAVIKETMRLHPPTPLLVPRETRKDAIIHGYKIKQNTIVYVNAWAIGRDPK  406
Ha_XP_022017533.1       AIVVWAMTLLIRNPVVMQKAQEEVRNVMGK----DGKISEDDLPKLAYLKAIIKETMRLYPPAPLLVPRETRKEATVHGYQIKQKTLVYVNAWAIGRDPK  410
Ha_XP_022017641.1       AIVVWAMTLLIRNPIVMQKAQEEVRNVMGK----NGKVSEDDLPKLAYLKAIIKETMRLYPPAPLLVPRETRKDATVHGYQIKQKTLVYVNAWAIGRDPK  410
Tc_TcJMH                ATVVWAMTALMKNPKVMRKAQEEVRTVGGKK----GAIDENDLAQLIYLKAIIKEIMRLYPAAPLLVPRETTKDTILQGYEIKEKTLVHVNAFAIARDPE  416
Tc_Tci_207965           ATVVWAMAALMKNPGAMKKVQEEVRNVVGKKVGKKDKVDEDDLPKLAYMKAVVKEIMRLYPTVPLLVPRETTKETILHGYRIKPKTLVFINALAIGRDPD  452
Tc_Tci_647136           ATVVWAMTALIKNPDVMRKAQEEVRNVAGKK----GAIDENDLAQLIYLKAIVKEIMRLYPPAPLLVPRETKKDTILHGY--------------------  264

Aa_PWA45353.1           ----------------------------------------------------------------------------------------------------
Aa_PWA46989.1           SWENPEEFLPERFLG-SDIDFRGNDFELIPFGAGRRICPGIPLGVVMVELLLANLVYLFDWELPVGMKKEDIDYEAKPGVTMHKKNELCLLAHVYS----  330
Aa_PWA54029.1           ----------------------------------------------------------------------------------------------------
Aa_PWA59404.1           SWENPEEFSPERFLG-SDIGFKGSDFELIPFGAGRRICPGISMGVNSVELFLANLIYSFDWGLPDGTKIEDIDSGVLPGLTMTNKKDLCLLAHFNSNSPN  586
Aa_PWA59405.1           ----------------------------------------------------------------------------------------------------
Aa_PWA95787.1           SWENPEKFLPERFLG-SDIDFRGNDFELIPFGAGRRICPGISLGVLMAELLLANLIYLFDWKLPVGMKIEDIDYEAKPGLTMHKKNELCLLAHVYM----  507
Cs_Cse_sc002631.1_g040.1SWDSPEEFLPARFLG-SDIDFKGNDFEFIPFGAGRRMCPGILMGTVTVDLLLANLLYLFDWGLPDGMQREDIDFEVMPGITLHKKNELYLLPHVYL----  379
Cs_Cse_sc002631.1_g060.1SWENPEEFLPERFLD-SDIDFKGNDFELIPFGAGRRICPGLSMGVVTVDLLLANLLYLFDWALPDGMQKEDIEFEVVPGITMHKKNELYLLAHEHK----  101
Cs_Cse_sc007503.1_g060.1SWENPDE---------------GNDFEFIPFGAGRRICPGISLGVVTAELLLANLVYLFDWGLPVSMKKEDIDYETRPGVTMHKKNELCLLAHVYS----  446
Cs_Cse_sc012992.1_g010.1SWENPEEFLPERFLG-SDIDFRGNDFELIPFGAGRRICPGISLGVLMAELLLANLIYLFDWKLPVGMQIEDIDYEAKPGLTMHKKNELCLLAHVYM----  506
Cs_Cse_sc024445.1_g030.1SWDSPEEFLPERFLC-SDIDFIGNGFTFIPFGAGRRMCPGILMGTVTVDLLLANLLYLFDWGLPDGMQKEDIDFEVMPGITLHKKNELYLLPHVYL----  502
Ha_XP_021983786.1       SWENPEEFSPERFLN-SDIGFKGNDFELIPFGAGRRICPGISMGVLEVELLLANLIYLFDWGLPDGMKKEDIDLEVMPGLTMTNKEDLCLLAQVYSGKAM  509
Ha_XP_021983800.1       SWDSPEEFLPERFLG-SDIDFKGNDFELTPFGAGRRICPGMTMGVVTVDLLLANLLYLFDWSLPDGIKKEDIDLEVMPGITMHKRNELCLLPHVYL----  499
Ha_XP_021983815.1       FWESPEDFFPERFLD-SDIDFKGNDLELIPFGAGRRICPGMSMGVVMVELLLANLLYLFDWGLPDGIKKEDIDLDVMPGLTMLKRNELCLLAHEVLYF--  503
Ha_XP_021983816.1       YWDRPEDFYPERFLG-SDIDFKGNDFELIPFGAGRRICPGMLMGVLMVELLLANLLYLFDWGLQHGMQKDDIDLDAMPGVAIHKKNELCLLAHEYI----  498
Ha_XP_022017027.1       SWESPEEFLPERFLG-SDIDFKGNDLELIPFGAGRRICPGMSIGVFMVELILANLLYLFDWGLPDGMVKEDIDYDAMSGITMHKRNELCLLARVL-----  500
Ha_XP_022017533.1       SWNNPGEFFPERFMGKCDIDFKGNDFELIPFGAGRRICPGMSIGVVTVELLLANLLYLFDWGLPDGIRKENIDFDAMPGITMHKRNELCLLAHVNF----  506
Ha_XP_022017641.1       SWNNPGEFFPERFMGKCDIDFKGNDFELVPFGAGRRICPGMSIGVVTVELLLANLLYLFDWGLPDGIRKEDIDFDVMPGITMHKRNELCVLAHVNF----  506
Tc_TcJMH                SWENPEEFIPERFLG-SDIDFRGNDFELIPFGAGRRICPGITLGVIMAELLLANLIYLFDWKLPVGMKIEDIDYEAKPGFTMHKKNELCLLAGVYL----  511
Tc_Tci_207965           SWENPEEFLPERFLD-SDIDFKGNDFGLIPFGAGRRICPGLSMGVVTVDLLLANLLYLFDWALPDGMNKEDIEFEVVPGITMHKKNELYLLAHEHVNHLL  551
Tc_Tci_647136           ----------------------------------------------------------------------------------------------------
```

Supplemental Figure 5. Molecular phylogenetic analysis of *T. cinerariifolium* jasmone hydroxylase (TcJMH). TcJMH-related proteins were searched by BLASTP against 14 different plant protein sets and incorporated into maximum likelihood tree analysis using ORTHOSCOPE method[56] (A). Proteins incorporated into this tree include 9 from *T. cinerariifolium*, 10 from *C. seticuspe*, 10 from *A. annua*, 10 from *H. annuus*, 10 from *Z. mays*, 10 from *V. vinifera*, 8 from *M. truncatula*, 10 from *S. lycopersicum*, 10 from *P. trichocarpa*, 10 from *O. sativa*, 9 from *G. raimondii*, 10 from *N. tabacum*, 10 from *G. max*, and 10 from *A. thaliana*. TcJMH nodes are indicated in blue; *T. cineariifolium* nodes and edges are indicated in red; other Asteraceae nodes and edges are indicated in orange. The amino acid sequence alignments for maximum likelihood tree analysis are shown in panel B.

Supplemental Figure 6.

A

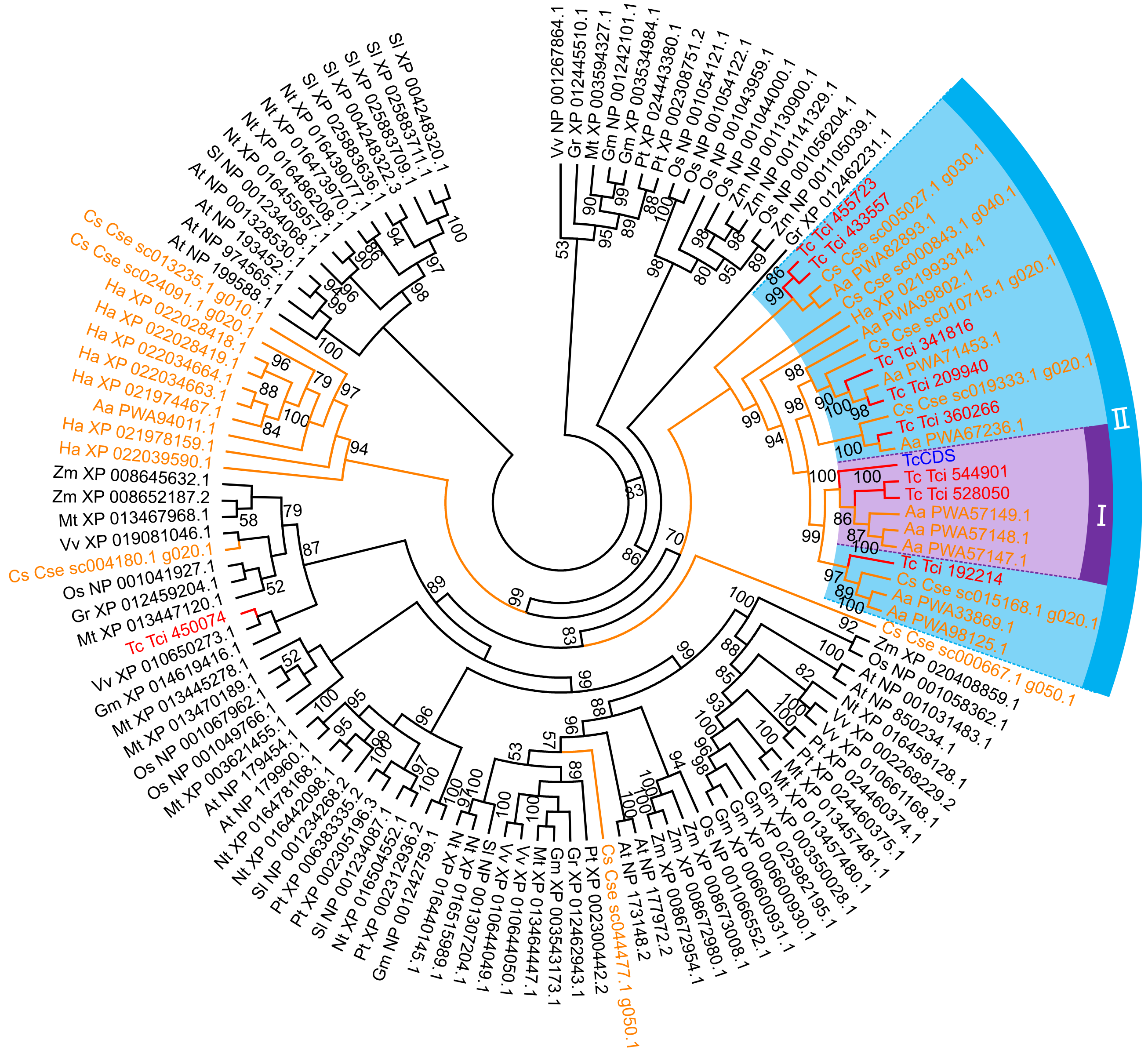

B

```
Aa_PWA33869.1           ------------------------------------------------------------------------------------------------MSSE 4
Aa_PWA39802.1           --------------------------------------------MAFSNRVVIGGYLTSKWAPWHASSRPSVGQPKNVLHCHQ---RSSDPLLSTTMSND 53
Aa_PWA57147.1           ----------------------------------------------------------------------------------------------------
Aa_PWA57148.1           ----------------------------------------------------------------------------------------------------
Aa_PWA57149.1           --------------------------------------------MACSSSLSSKWASWNASSFPHPSVQPFVTR-KNVVRYHKQTSES---TITTTLSSN 52
Aa_PWA67236.1           --------------------------------------------MAFSNRVVIGGNLTSKWAPWHASSQPSVGKLKNVLHCHK---RSSDSLLSTTMSND 53
Aa_PWA71453.1           ----------------------------------------------------------------------------------------------------
Aa_PWA98125.1           --------------------------------MNLPLLMITYKCFSLVGSLSSKWASWNGSSHLQTSTRPLVAAGKYVVRYQTPATDSIYSTLTTTMSSE 68
Cs_Cse_sc010715.1_g020.1----------------------------------------------------------------------------------------------------
Cs_Cse_sc015168.1_g020.1----------------------------------------MAFCNSLVGGLSSKWASWNGSSHLQTPTRPLVDAGKYVVRYQTPATDSIYSTLTTTMSSE 60
Cs_Cse_sc019333.1_g020.1--------------------------------------------MAFSNRVVIGGNLTSKWASWHASSRPSVGQPKNVLHCHK---RPSYSLLSTTMSND 53
Ha_XP_021974467.1       ----------------------------------------------------------------------------------------------------
Ha_XP_021993314.1       ----------------------------------------------------------------------------------------------------
Ha_XP_022028418.1       ----------------------------------------------------------------------------------------------------
Ha_XP_022028419.1       ----------------------------------------------------------------------------------------------------
Ha_XP_022034663.1       ----------------------------------------------------------------------------------------------------
Ha_XP_022034664.1       ----------------------------------------------------------------------------------------------------
Tc_TcCDS                ------------------------------------------MSWCLLCGLSSKWASWDASSRPHPSVQPFVTR-KNVVRYHKPTSESSYSTLTTTLSSN 57
Tc_Tci_192214           ----------------------------------------------------------------------------------------------------
Tc_Tci_209940           ----------------------------------------------------------------------------------------------------
Tc_Tci_341816           RMQNVPYASVVGSIMYVVRCTRRDVAFVQNITSRFQQNPNELCWTVVKTILKVEIGGGNLTSKWASCHASSRPSIGTLKNVLHCHKRTSDSLLSTTMSND 750
Tc_Tci_360266           TSEHCKRFKKVRIDVGARPSVTIDDLFWADHGRFQYQCLSLVVDLRWLWXVVIGANLTSKWASSHASSRPSLGQPKTVLNCHK---WTSDSLLSTTMSND 118
Tc_Tci_528050           ----------------------------------------------------------------------------------------------------
Tc_Tci_544901           ----------------------------------------------------------------------------------------------------

Aa_PWA33869.1           LNSEFMHVYDTLKSELVHDPAF--ASDDNSCQWVERMIDYNVPG-------GKMVRAFSVVDSCQLLKGEKMTE-DEVFLACALGWCTEWLQASLVVFDD 94
Aa_PWA39802.1           LKSKFMQVYDQLKSELIHDPAF--AFDDVSRQWVDKMIDYIVPG-------GKMTRGLSVVDSYQLLKREELTD-DEAFL----------LQAYYLLYDD 133
Aa_PWA57147.1           ---------------------------------MFAVTKKMMYA-------GKMVRGYSVVDSYQLLKGQELTE-EEAFLVCALGWCTEWFQAFILAHDD 59
Aa_PWA57148.1           ----------------------------------------------------------------------------------------------------
Aa_PWA57149.1           LNSQFMQVYETLKSELIHDPSF--EFDDDSRQWVERMIDYTVPG-------GKMVRGYSVVDSYQLLKGQELTE-EEAFLVCALGWCTEWFQAFILAHDD 142
Aa_PWA67236.1           LKSKFMQVYDQLKSELIHDPAF--EFDDVSRQWVDKMIDYNVPG-------GKMFRGLSVVESYQLLKGEELAD-GEVFLACALGWCIEWLQAYFLVLDD 143
Aa_PWA71453.1           -----MQVYETLKSELIHDPSF--EFDDDSRQWVERMIDYTVPG-------GKMVRGYFVVDSYQLLKGQELTE-EEAFPVCALGWCTEWFQAFILAHDD 85
Aa_PWA98125.1           LNSEFMHVYDTLKSELVHDPAF--VSDDNSCEWVERMIDYNVPG-------GEMVRAFSVVDSCQLLKGEKMTE-DEVFLACALGWCTEWAEF------- 151
Cs_Cse_sc010715.1_g020.1-----------------------------------------------------MTRGLSVVDNYQLLKGEELTD-DEAFFVCTLGWCIEWLQVYYLLYDD 46
Cs_Cse_sc015168.1_g020.1LNSEIMHVYDTLKSELVHDSAF--VSDDNSSQWVERMIDYNVPG-------GKMVRAFSVVDSCQLLKGEQMTE-DEVFLACALGWCTEWLQASLVVFDD 150
Cs_Cse_sc019333.1_g020.1LKSKFMQVYDQLKSELIHDPAF--EFDDVSRQWVDNMIDYNVPG-------GKMFRGLSVVESYQLLKGEELAD-GEVFLVCALGWCIEWLQAYGLVLDD 143
Ha_XP_021974467.1       ----------MRMEDLISDRLKLLNILLVIISNVNVITRYNFDP---------------------VMRSRLWWYNFDPVMRSRLGWCIEWLQANFLVLDD 69
Ha_XP_021993314.1       ----------------------------------------------------------------------------------------------------
Ha_XP_022028418.1       -----------------------------------MFCRYNFDP---------------------VMRSRLWWYNFDPVMRSRLGWCIEWLQANFLVLDD 44
Ha_XP_022028419.1       -------------------------------------------------------------------------------MRSRLGWCIEWLQANFLVLDD 21
Ha_XP_022034663.1       ----------MRMEDLISDRLKLLNILLVIISNVKVITRYNFDP---------------------VMRSRLWWYNFDPVMRSRLGWCIEWLQANFLVLDD 69
Ha_XP_022034664.1       ----------MRMEDLISD--------------------RYNFDP---------------------VMRSRLWWYNFDPVMRSRLGWCIEWLQANFLVLDD 50
Tc_TcCDS                LDSQFMQVYETLKSELIHDSSF--EFDDDSRQWVERMIDYTVPR-------GKMVRGYSVVDSYQLLKGEKLTE-DGAFLVCALGWCTEWLQAFLLIHDD 147
Tc_Tci_192214           ------------------------------------MIDYNVPG-------GKMVRAFSVVDSCQLMKGEKMTE-DEVFLACALGWCTEWVS-------- 48
Tc_Tci_209940           -----MHVYDTLKFELVHDQAF--VSDDNSRQWVERMIDYNVPG-------GKMVRAFSVVDSCQLMKGEKMTE-DEVFLACALGWCTEWLQASFVVLDD 85
Tc_Tci_341816           LKSKFMQVYDHLKSELIHDPAF--EFDDVSRQWVDKMIDYIVPGEKSLMYAGKMTRGLSVVDSYQLLKGEELTY-DEAFLVCTLGWCIEWLQAYFLLYDD 847
Tc_Tci_360266           LKSKFMQVYDHLKSELIHDPAF--EFDDVSRQWVDKMIDYNVPG-------GKMLRGLSVVESYQLLKGEELAD-GEVFLACALGWCIEWLQAYFLVLDD 208
Tc_Tci_528050           -----MGFLGFLFSSAPISSTF--CDSEECG----TMSDYTIP--------GDMA-------SFSIILMQEIN--------CLLLMCTERLQAFILALED 66
Tc_Tci_544901           ----------------------------------------------------------------------------------------------------

Aa_PWA33869.1           IMDDSHTRRGRSCWFRLPEVCMTAVNDGHMLRNHVHRILKKHFHEKAYYVHLVDLFNEAEFQTVCGQMIDAIAT-LEGKKDLSKYTMSVTPCNLFLPHST 193
Aa_PWA39802.1           IMDGSHYRRGQPCWFRLPEVGMIAVNDGVLLRSHVTRILKKHFQAKAYYVHLLDLFNEAEFQTVSGQMIDVITT-LVGEKGLSNYSLSLNRRIIQYKCSY 232
Aa_PWA57147.1           IMDGSHTRRGQPCWFRLPEVGVVAINDGVLLRNHVHRILKKHFQGKAYYVHLVDLFNETEFQTISGQMIDTIAR-LAGQKDLSKYSMSLNRKIVQYKGSY 158
Aa_PWA57148.1           -MDGSHTRRGQPCWFRLPEVGVVAINDGVLLRNHVHRILKKHFQGKAYYVHLVDLFNETEFQTISGQMIDTIAR-LAGQKDLSKYSMSLNRKIVQYKGSY 98
Aa_PWA57149.1           IMDGSHTRRGQPCWFRLPEVGVVAINDGVLLRNHVHRILKKHFQGKAYYVHLVDLFNETEFQTISGQMIDTIAR-LAGQKDLSKYSMSLNRKIVQYKGSY 241
Aa_PWA67236.1           IIDDSHIRRGHPCWFRLPEVGMVAVNDAILLRSHIPRILKKHFSGKAYYVNLLDLFNET-FKRYRGEMIDAITT-LVGEKDLSKYSLSLNRQITQYKCSY 241
Aa_PWA71453.1           IMDGSHTRRGQPCWFRLPEVGVVAINDGVLLRNHVHRILKKHFQGKAYYVHLVDLFNEIDTGSAR------------------------QLEQNFKVV-- 159
Aa_PWA98125.1           -------------------------------------------------------------QTVCGQMIDAIAT-LEGKKDLSKYTMSLTPCNLFLPHST 189
Cs_Cse_sc010715.1_g020.1IMDGSHYRRGQPCWFRLPEVGMIVV-------------LKKHFQAKAYYMHLLDLFNE------------------------------------------ 91
Cs_Cse_sc015168.1_g020.1IMDGSHTRRGRSCWFRLPEVGMTAVNDGHMLRNHVHRILKKHFHEEAYYVHLVDLFNEAEFQTVCGQMIDVIAT-LDGKKDLSKYTMSLNRRIFEYKSSY 249
Cs_Cse_sc019333.1_g020.1IIDDSHIRRGHPCWFRVPEVGMVAVNDAVLLHSQIPRILKKYFSGKAYYVNLLDLFNEANFQIVSGQMIDVITK-LVGEKDLSKYSLSLNRRIIQYKCSY 242
Ha_XP_021974467.1       IMNESHTHT---------------------------------------------------------------------ENFMTGYQLVFYRENLTLGCSH 100
Ha_XP_021993314.1       ---------------------MIAVNDGALLSNHVRRILKKNFHGKAYYAHLLDLFNEVELQTIYGQMIDATTTTLVGEKDLSKYTLFLKWRILQYKSSY 79
Ha_XP_022028418.1       IMDESHTNR---------------------------------------------------------------------ENFMTGYQLVFYRENLTLGCSH 75
Ha_XP_022028419.1       IMDESHTNR---------------------------------------------------------------------ENFMTGYQLVFYRENLTLGCSH 52
Ha_XP_022034663.1       IMNESHTHT---------------------------------------------------------------------ENFMTGYQLVFYRENLTLGCSH 100
Ha_XP_022034664.1       IMNESHTHT---------------------------------------------------------------------ENFMTGYQLVFYRENLTLGCSH 81
Tc_TcCDS                IMDGSHTRRGQPCWFRLPGVGVVAINDGVLLRNHVHRILKKHFKGKAYYVHLLDLFNETEFQTISGQMIDTIAR-LAGQKDLSKYSMSLNSRIAQYKSSY 246
Tc_Tci_192214           ----------------------------------------------------------------------------------------------------
Tc_Tci_209940           IMDASHTRRGRSCWYRLPEVGMTAVNDGLMLRNHVHRILKEHFHDKAYYMQLVDLFNEAEFQTVCGEMIDIIAT-LDGKKDLSKYTMSLQMHNDIMAAGS 184
Tc_Tci_341816           IMDGSHYRRGQPCRFRLPEVGMIAVNDGVLLCSHVARILKKHFQGKAYYVHLLDLFNEAEFQTISG-----------------------EMIDVISTLVG 924
Tc_Tci_360266           IIDDSHIRRGHPCWFRLPEVGMVAVNDAILLRSHIPRVLKKHFSGKVYYVNLLDLFNEIDFQTVSGEMIDAITT-LVGGKDLSKYSLSVNRRIVQYKCSY 307
Tc_Tci_528050           IMDGSHTRRGQPCWFRLPEVGVAAINDGVLLRNHVYRILKKHLQGKPYYMHLLDLFNETEFQTISRQMINTISR-LARQKDLLKYTTTLNRRILQYKSSY 165
Tc_Tci_544901           -MDGSHTRRGQPCWFRLPEVGVAAINDGVLLRNHVYRILKKHLQGKPYYMHLLDLFNETEFQTISRQMINTISR-LARQKDLLKYTTTLNRRILQYKSSY 98

Aa_PWA33869.1           FNFLNTLPMVHGCRTFCTTRENACNFESLFDVCVAYLKFTMSFCLICSNCRIFEYKSSYYSFYLPIACALLMFGENLDDHVLAKDVLIEIGIYYQVQ--- 290
Aa_PWA39802.1           YSFYLP-----------------------------------------------------------VACALLMIGENLEDHVQVKDILVEMGIYYQVQ--- 270
Aa_PWA57147.1           YSCYLP-----------------------------------------------------------IACALLMFGENLEDHVQVKDILVELGMYYQIQ--- 196
Aa_PWA57148.1           YSCYLP-----------------------------------------------------------IACALLMFGENLEDHVQVKDILVELGMYYQIQ--- 136
Aa_PWA57149.1           YSCYLP-----------------------------------------------------------IACALLMFGENLEDHVQVKDILVELGMYYQIQ--- 279
Aa_PWA67236.1           YSCYLPF-----------------------------------------------------------ACALLMLGENLDDHAQVKDIVVEMGIYAQVQ--- 279
Aa_PWA71453.1           --------------------------------------------------------------------YCLVITVKIVQ--------------------- 170
Aa_PWA98125.1           FNFLNTLPM--------------------------------------------------------IACTLLMFGENLDDHVLAKDVLIEIGIYYQVQ--- 230
Cs_Cse_sc010715.1_g020.1-----------------------------------------------------------------VVCALLMIGENMDDHVQVKDILVEMGIYYQVQ--- 123
Cs_Cse_sc015168.1_g020.1YSFYLPVSTS-------------------------------------------------------IACALLMFGENLDDHVLAKDILVEIGIYYQVQ--- 291
Cs_Cse_sc019333.1_g020.1YSCYP--------------------------------------------------------------------SKNLDDHVQVNDILVEMDIYGQVQ--- 271
Ha_XP_021974467.1       SR-----------------------------------------------------------------------CEYINHF-----FLQTVNVNCQPP--- 121
Ha_XP_021993314.1       YSFYLP-----------------------------------------------------------VACALLLLGENPDDYVQMKDILVEMGIYYQIQ--- 117
Ha_XP_022028418.1       SR-----------------------------------------------------------------------FGSLSAIREMEGAYRECAVKETST--- 101
Ha_XP_022028419.1       SR-----------------------------------------------------------------------FGSLSAIREMEGAYRECAVKETST--- 78
Ha_XP_022034663.1       SR-----------------------------------------------------------------------CEYINHF-----FLQTVNVNCQPP--- 121
Ha_XP_022034664.1       SR-----------------------------------------------------------------------CEYINHF-----FLQTVNVNCQPP--- 102
Tc_TcCDS                YSCYLP-----------------------------------------------------------IACALLMFGENLEDHVQVKDILVELGMYYQIQ--- 284
Tc_Tci_192214           -----------------------------------------------------------------------------------KDVLIEMGIYYQVQ--- 62
Tc_Tci_209940           KKRPPML---------------------------------------------------------APDTYLNLFTMKIHHNDEFSKPLERRYKFDELDYVD 227
Tc_Tci_341816           KK--------------------------------------------------------------GLSNYSLSLNRRIIQ--------------------- 941
Tc_Tci_360266           YSCYLPLSIFRLFSILCT----------------------------CLYFPIILNDQIYHFLCFKFECALLMLGENLDDHVQVKDILVEMGIYAQVQ--- 376
Tc_Tci_528050           YSCYLP-----------------------------------------------------------IACALFMFGKNLEDHVQMKDILVELGMYDQIQ--- 203
Tc_Tci_544901           YSCYLP-----------------------------------------------------------IACALFMFGKNLEDHVQMKDILVELGMYDQIQ--- 136
```

```
Aa_PWA33869.1           ---------NDYLDIFGEPNVFGKTGTDIEECKCSWLIVKAMELANEEQKKILN-----ENYGMTDPEKVAKVKELY------------RNLNP------  358
Aa_PWA39802.1           ---------DDYFDTFGDPKVVGKIGTDIEDCKCSWLIAKALELANEEQKKILN-----ENYGRKDLDKVAKVKELY------------HTLNLQ-GAYE  343
Aa_PWA57147.1           ---------NDYLDTFGDPNVFGKTGTDIEECKCSWLIAKALELSNEEQKKILS-----ENYGKNDPAKVAKVKEVY------------HALDLK-SAYE  269
Aa_PWA57148.1           ---------NDYLDTFGDPNVFGKTGTDIEECKCSWLIAKALELSNEEQKKILS-----ENYGKNDPAKVAKVKEVY------------HALDLK-SAYE  209
Aa_PWA57149.1           ---------NDYLDTFGDPNVFGKTGTDIEECKCSWLIAKALELSNEEQKKILS-----ENYGKNDPAKVAKVKEVY------------HALDLK-SAYE  352
Aa_PWA67236.1           ---------DDYLDTFGDPNVVGKIGTDIENFKCSWLVAKALELSNEEQKKILS-----KNYGIKDPVKVANVKKLY------------HTLNLE-GVYQ  352
Aa_PWA71453.1           ---------YKGS-----------------YYSCYPS---DCVCTPYVG--------------EICEDQ------------------MLQVKRKK-NSL-  207
Aa_PWA98125.1           ---------NDYLDIFGEPNVFGKTGTDIEECKCSWLIVKAMELANEEQKKILN-----ENYGMKDPEKVAKVKELY------------HTLNLE-GAYE  303
Cs_Cse_sc010715.1_g020.1---------DDYLDTFGDPKVVGKVSPPISFILDDHAFLRNHNLCTICDIVSDW-----NRYRRLQVYLIAAFLRLYLYTGRPHMFLLPHLLDIAYGAYE  209
Cs_Cse_sc015168.1_g020.1---------NDYLDTFGEPNVFGKTGTDIEECKCSWLIVKAMEQANEDQKKILN-----ENYGMTDPEKVAIVKELY------------HTLNLE-GAYE  364
Cs_Cse_sc019333.1_g020.1---------DDYFDTFGDPNVVGKIGTDIENFKCSWLVAKALELSNEEQNKFLS-----ENYGIKDPVKVANVKKLY------------RTLNLE-RVYE  344
Ha_XP_021974467.1       ------------FVIFIS--FGGYSGLFQTFFKTIMHLRICTNP--------------------------------------------------------
Ha_XP_021993314.1       ---------NDYLDTFGDHNTVGKIGTDIEERKCSWLVAKALELASEEQKQILY-----ENYGKKDQACVEKVKELY------------HTLNLQ-GVYE  190
Ha_XP_022028418.1       ------------LWTYIIKVYYLRTGLDRSIWT-------CTNP--------------------------------------------------------
Ha_XP_022028419.1       ------------LWTYIIKVYYLRTGLDRSIWT-------CTNP--------------------------------------------------------
Ha_XP_022034663.1       ------------FVIFIS--FGGYSGLFQTFLR-----PLCT----------------------------------------------------------
Ha_XP_022034664.1       ------------FVIFIS--FGGYSGLFQTFLR-----PLCT----------------------------------------------------------
Tc_TcCDS                ---------NDYLDTFGDPNVFVKTGTDIEECKCSWLIAKALELANEEQKKILS-----ENYGIKDPAKVAKVKELY------------HALDLK-GAYE  357
Tc_Tci_192214           ---------NDYLDTYGDPNVFGKTGTDIEECKCSWLIVKALELANDEQKKILS-----ENYGSTDPEKVAKVKELY------------QTLKLK-GVYE  135
Tc_Tci_209940           IVDSDMFFLYEWSGMLKELGLGDNNKIPFTYYRIHVMSMDDCLVSLMADVGVIKLLNYVIGCKEIEEGLAVGETELY----------MRGVLRPK-GSFL  316
Tc_Tci_341816           ---------YKCS-----------------YYSFYLP--VACSLLMIGEN-------------------------------------LDDHVQVK-DILV  975
Tc_Tci_360266           ---------DDYFDTFGDPNIVGKIGTDIESFKCSWLVAKALELSNVEQKKILS-----ENYGIKDPVKVAKVKKLY------------HTLNLE-AVYE  449
Tc_Tci_528050           ---------NDYLDTFGDPDV-------------------------------------------------------------------------------
Tc_Tci_544901           ---------NDYLDTFGDPDV-------------------------------------------------------------------------------

Aa_PWA33869.1           --------------------------------------------------
Aa_PWA39802.1           DYEKSTHEELITSIEAH---PSKALRAVLKSFLEKIYKRQK--------- 381
Aa_PWA57147.1           DYETNLYEKSMKAIKAL---PSITVQAVLKSCLEKMYEGHK--------- 307
Aa_PWA57148.1           DYETNLYEKSMKAIKAL---PSITVQAVLKSCLEKMYEGHK--------- 247
Aa_PWA57149.1           DYETNLYEKSMKAIKAL---PSITVQAVLKSCLEKMYEGHK--------- 390
Aa_PWA67236.1           DYENKTREELIKSIDVL---PNKAVQAVLRSFLKKLFKRRK--------- 390
Aa_PWA71453.1           --------------------------------------------------
Aa_PWA98125.1           DYESNIYAELIKSIEAL---PSKAVQAVLKSCPGKIYKGHKAHSMPTILR 350
Cs_Cse_sc010715.1_g020.1DYEKSTHEELIRSIEAH---PSKALQAVLKSFLETIYKRQK--------- 247
Cs_Cse_sc015168.1_g020.1DYESNIYAELIKSIESV---PSKAVQAVLKSCLGKIYKGHK--------- 402
Cs_Cse_sc019333.1_g020.1DYENKTHEELIESIEVL---PNKAVQAVLRSFLKKLFKRRK--------- 382
Ha_XP_021974467.1       --------------------------------------------------
Ha_XP_021993314.1       EYEKMTHEEFMKLIESH---PSNVYKQC---------------------- 215
Ha_XP_022028418.1       --------------------------------------------------
Ha_XP_022028419.1       --------------------------------------------------
Ha_XP_022034663.1       --------------------------------------------------
Ha_XP_022034664.1       --------------------------------------------------
Tc_TcCDS                DYETNLYEKSMKAIKAH---PSISVQAVLKSCLEKMYKGHK--------- 395
Tc_Tci_192214           EYESNIYADLIKSIEAH---PSKAVQGVLKSCLGKIYKGHN--------- 173
Tc_Tci_209940           KFVEGDIAGVMPPMVYISMLFSWFAWHVEDHDFHNIKYMHKGVGKTRYCA 366
Tc_Tci_341816           EM------------------------------------------------ 977
Tc_Tci_360266           DYENKTHKELIKSIEVL---PNKAVQEVLKSFLRKLYKRRK--------- 487
Tc_Tci_528050           --------------------------------------------------
Tc_Tci_544901           --------------------------------------------------
```

Supplemental Figure 6. Molecular phylogenetic analysis of *T. cinerariifolium* chrysanthemyl diphosphate synthase (TcCDS). TcCDS-related proteins were searched by BLASTP against 14 different plant protein sets and incorporated into maximum likelihood tree analysis using ORTHOSCOPE method[56] (A). Proteins incorporated into this tree include 9 from *T. cinerariifolium*, 10 from *C. seticuspe*, 10 from *A. annua*, 8 from *H. annuus*, 9 from *Z. mays*, 7 from *V. vinifera*, 9 from *M. truncatula*, 9 from *S. lycopersicum*, 8 from *P. trichocarpa*, 10 from *O. sativa*, 4 from *G. raimondii*, 10 from *N. tabacum*, 9 from *G. max*, and 10 from *A. thaliana*. TcCDS nodes are indicated in blue; *T. cineariifolium* nodes and edges are indicated in red; other Asteraceae nodes and edges are indicated in orange. The amino acid sequence alignments for maximum likelihood tree analysis are shown in panel B.

Supplemental Figure 7.

A

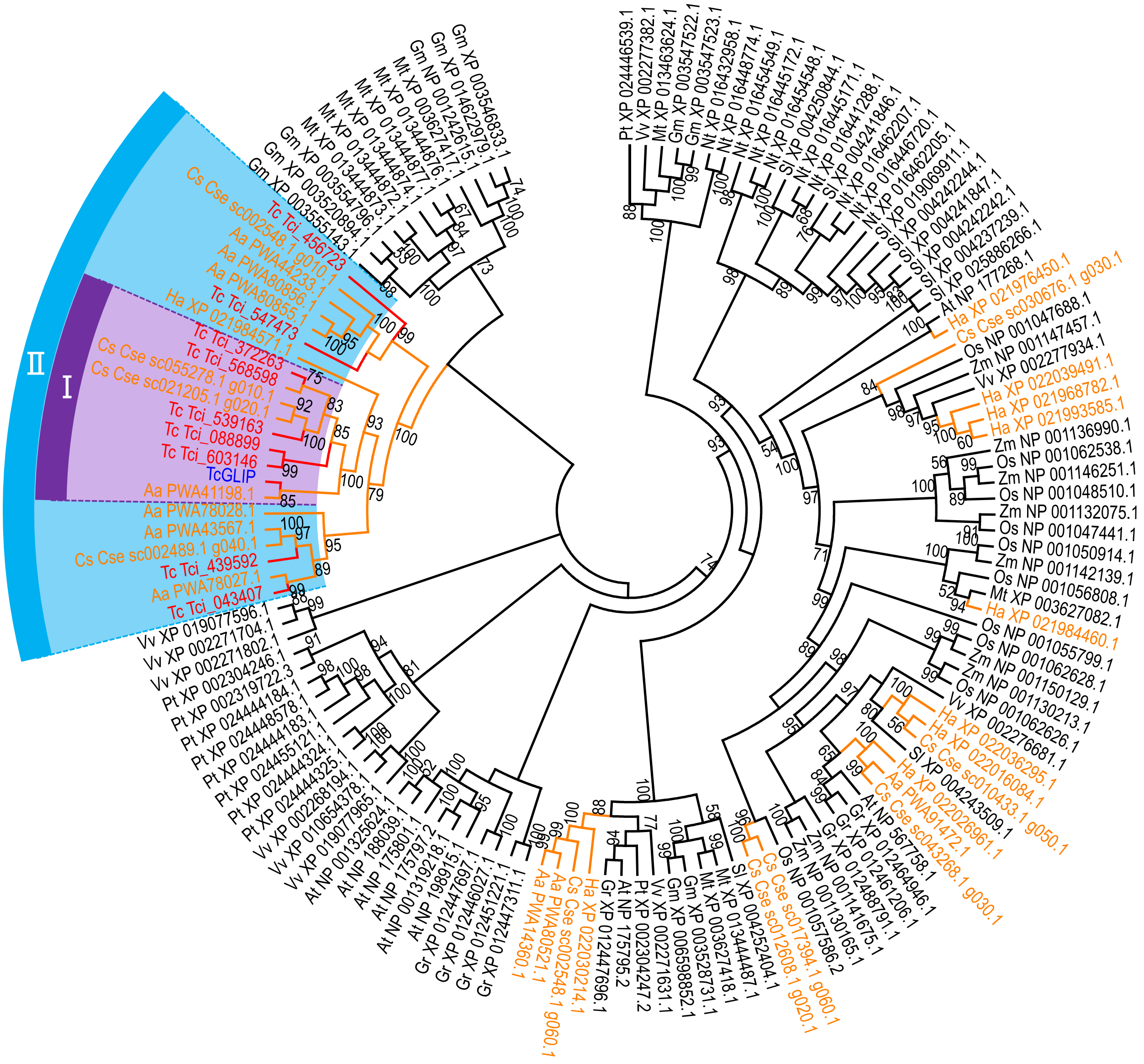

B

```
Aa_PWA41198.1           ------MAIASSKLGALVFVAVLCLSLPTG------------------------CLSSQQ-AALFVFGDSLFD-----------PGNNHHIN------TT 52
Aa_PWA43567.1           ------MTVASSKSG--FLVLFVFCLSVPTD----------------------SWGNIEKHTALFVFGDSLFD-----------PGNNNYIN------TT 53
Aa_PWA78027.1           IPDFIAMAIASSKSSRFAVVVIM-CLLIPTK----------------------CRENIHKQVLVFVLGDSLFD-----------PGNNNYIN------TT 141
Aa_PWA78028.1           IEDVFAMAVANRKSSFVLLLIIAVVVLCPLTRI-------------------DCHENIHKEVAVFVFGDSFFD-----------PGNNNYIN------TI 483
Aa_PWA80855.1           -----------------------------SSHG----------------------HYDKEHIPLFVFGDSLFD-----------PGNNNYIN------TT 53
Aa_PWA80856.1           -----------------------------SSHG----------------------HYDKEHIPLFVFGDSLFD-----------PGNNNYIN------TT 53
Cs_Cse_sc002489.1_g040.1FKVINPMTVASSKSG--FLVLFVFCLSVPID----------------------SWGNIEKHVALFVFGDSLFD-----------PGNNNYIN------TT 135
Cs_Cse_sc002548.1_g010.1-----------------------------SCHG----------------------HYDKEHVPLFVFGDSLFD-----------PGNNNYIN------TT 53
Cs_Cse_sc021205.1_g020.1-------AELFWKGDPMVTGPYNLKALFDAMAVASSKLGALVFVAILCLSLPAVCLSSQQAALFV-FGDSLFD-----------PGNNHHIN------TT 423
Cs_Cse_sc055278.1_g010.1-------AELFWKGDPMVTGPYNLKALFDG------------------------CLSSQQAALFV-FGDSLFD-----------PGNNHHIN------TT 400
Ha_XP_021984571.1       ------MAIADGKSVFLLLPIVVVFSLSIPT---------------------GCLS-----SALFVFGDSFFD-----------PGNNNYIN------TI 51
Tc_TcGLIP               ------MAVASRKLGALVLVAVLCLSLPTG------------------------CLSSQQAAALFIFGDSVFD-----------PGNNNHIN------TH 53
Tc_Tci_043407           ------MANTSSESRRFAAVIIITCLSIPTN----------------------CHANIHKQVLVFVFGDSLFD-----------PGNNNYIN------TT 55
Tc_Tci_088899           ----------------------------------------------------------------------------------------------------
Tc_Tci_372263           ---------------------------KSG------------------------CLSSQQAALFV-FGDSFFD-----------PGNN-HIN------TA 30
Tc_Tci_439592           ------LSIYSRTFT--NRQYTDMVIGNLTK----------------------VIKGIHKKGGRKFGFLTVXD-----------PGNNNFIK------TI 87
Tc_Tci_456723           -------NPSNDSGGCIEAASLLAKLHNQALAK----------------------SLKRLAKQLHGFKYSLYDFHTNLNQRLKHPSKYGYKQGKTACCGT 234
Tc_Tci_539163           -------AKMFWKGYPPVTGPYNLKALFDG------------------------CLSSQQAALFV-FGDSLFD-----------PGNNHHIN------TT 101
Tc_Tci_547473           -------AELFWDGDSSVATPYNLKAFFHASYR----------------------NMISKHVALFVFGDSLYD-----------PGNNNYIN------TT 108
Tc_Tci_568598           ----------------------------------------------------------------------------------------------------
Tc_Tci_603146           -------EMTFEEAIGRLKTYEERIKYKGK------------------------QVDNQDRLLFTRYGEQGRR-----------RGHG-ESN------QS 83

Aa_PWA41198.1           ADFQANFWPYG---QSYFSLPTG------------------RFSDGRLIP----------------------DFIAEYASLP-LIPAYLEP-NNDFTHGV 107
Aa_PWA43567.1           ASFQANYWPYG---ESYFNPPTG------------------RFSNGRLIP----------------------DFIAEYARLP-LIPPYLEPGNNEFAYGA 109
Aa_PWA78027.1           TAFQANYTPYG---ESYFNPPTG------------------RFSNGRIIP----------------------DFIAEYAGLP-LIPAYLEPWNNEFTHGA 197
Aa_PWA78028.1           PVFQANYLPYG---ESYFSPPTG------------------RFSDGRLIP----------------------DFIAEYARLP-FIPPYLEPGNNAFTYGA 539
Aa_PWA80855.1           PDFQANYWPYG---VSYFHPASG------------------RFSDGRILS----------------------DFIAEYAGLP-LIPTYLDPQNDDFLYGA 109
Aa_PWA80856.1           PDFQANYWPYG---VSYFHPASG------------------RFSDGRILS----------------------DFIAEYAGLP-LIPTYLDPQNDDFLYGA 109
Cs_Cse_sc002489.1_g040.1ASFQANYWPYG---ESYFSPPTG------------------RFSNGRLIP----------------------DFIAEYARLP-LIPPYLEPGNNEFAYGA 141
Cs_Cse_sc002548.1_g010.1PDFQANYWPYG---VSYFNPASG------------------RFSDGRILS----------------------DFIAEYAGLP-LIPTYLDPRNDDFLYGA 109
Cs_Cse_sc021205.1_g020.1IDFQANFWPYG---QSYFSLPTG------------------RFSDGRLIP----------------------DFIAEYASLP-LIPAYLEP-NNDFTHGA 478
Cs_Cse_sc055278.1_g010.1ADFQANFWPYG---QSYFSLPTG------------------RFSDGHLIP----------------------DFIAEHASLP-LIPAYLE---------- 446
Ha_XP_021984571.1       TDFQANHWPYG---QSYFSNPSG------------------RFSDGRLIP----------------------DFIAEYAKLP-PIPAYLEPSNKEYTHGA 107
Tc_TcGLIP               VNFKANFWPYG---QSYFSSPTG------------------RFSDGRIIP----------------------DFIAEYASLP-IIPAYLEP-NNDFTHGA 108
Tc_Tci_043407           AAFQANFTPYG---ESYFYPPTG------------------RFSNGRLMP----------------------DFIAEYAGLPPLIPAYLEPGNNEFTYGA 112
Tc_Tci_088899           -----------------------------------------------------------------------------------MIPAYLEP-NNDFTHGA 16
Tc_Tci_372263           TDFQANFWPYA---EYAS--------------------------------------------------------------LP-IIPAYLEP-NNDFTHGA 63
Tc_Tci_439592           PVFQANFLPYG---ESHFTPPTG------------------RFSNGRLIP----------------------DFIAEYARLP-PIRPYLAPGNTELTYGT 143
Tc_Tci_456723           GRFRGTFSCGGKRPVKEFQLCNNPNEYVARVLGTI------SMSMSRFLFLAIHFLILETITTLTLQQASKLTXIAEYARLP-LIPAYLDPQHNEFVHGA 327
Tc_Tci_539163           ADFQANFWPYG---QSYFSPPTG------------------RFSDGRLIP----------------------DFIGR----------------------- 135
Tc_Tci_547473           ADFRANFWPYG---VSYFSPPGG------------------RFSDGRLIP----------------------DFIAEFAGLP-LIPTYLDPRNTEFTYGA 164
Tc_Tci_568598           ------------------------------------------------------------------------------------------------MEQI 4
Tc_Tci_603146           RGQENNFKKET---HNNSNKLTHDKSKETITTSTPLLIFKRTFSHMVNPT----------------------SVLQLELCMP-IIPAYLEP-NNDFTHGA 156

Aa_PWA41198.1           NFASGGAGALIESHAGL----------------------KNLGDTKSKQLLSNAVYLFSCGGNDYQSP----------FYP--YTREQYVDLVIRNMTKV 173
Aa_PWA43567.1           NFASVGAGALIDTRAGFV---VDLQTQLRYLGELENNYRKNLGDTEARQLLSSAVYLFSCGANDYGTFVDNNLSI--Y-HL--FTDEQYTDMVIGNLTNV 201
Aa_PWA78027.1           NFASAGAGALIETFAGFV---VDLQTQLRYFGDLEDHYRQNLGDTKARQLLSSAVYLFSSGANDYLSPVGNNISI--Y-YP--YTHEQYTDMVIGNLTNV 289
Aa_PWA78028.1           NFASSGAGTLIDNLAGLV---VDLQTQLQYFGDLENLYRQNLGDTKAEQLLSNAVYLFSCGANDYISHVPNNLSISSIYPT--LNNEQYTELVIGNLTNV 634
Aa_PWA80855.1           NFASGGAGALVESNAGFV---VDLKTQLEYFGDLEKQFRRNLGDAKAEQLLSNAVYMFGCGGNDYLSSVGNNDS---ILYP--YTHEQYVRMVIGNLTDV 201
Aa_PWA80856.1           NFASGGAGALVESNAGFV---VDLKTQLEYFGDLEKQFRRNLGDAKAEQLLSNAVYMFGCGGNDYLSSVGNNDS---ILYP--YTHEQYVRMVIGNLTDV 201
Cs_Cse_sc002489.1_g040.1NFASAGAGALIDTRAGFV---VDLQTQLRYLGEVENNYRKNLGDKEARKLLSSAVYLFSCGANDYGTFVDNNLSI--Y-HL--FTNEQYTDMVIGNVTNV 150
Cs_Cse_sc002548.1_g010.1NFASGGAGALVESNAGFV---VDLKTQLEYFGDLEKQFRQNLGDAKAEELLSNAVYMLNGGGNDYFSSVGNNDS---ILYP--YTHEQYVGMVIGNLTDV 201
Cs_Cse_sc021205.1_g020.1NFASGGAGALIESHTGF---VVDLQTQLRYFGDLVNLYRQNLGDTKSKQLLSDAVYLLGCGANDYQSP----------YYP--YTREQYVDLVIGNMTNV 563
Cs_Cse_sc055278.1_g010.1------PNNDFTHGANFGPGRTDLQTQLRYFGDLVNHYRQNLGDTKSRQLLSDVVCLFSCGANDYQIP----------YYP--YTREQDMDLVIGNMTNV 528
Ha_XP_021984571.1       NFASGGAGTLIDTYAGFV---VDLHTQLRHFGDLVNHYRQNLGDTKARQMLSSAVYLFSCGSNDYISPVVNNQ---SIYYQ--YTHQQYVDMVIGNLTTV 199
Tc_TcGLIP               NFASAGAGALIASHAGLA---VGLQTQLRYFGDLVDHYRQNLGDIKSRQLLSDAVYLFSCGGNDYQSP----------YYP--YTQEQYVDIVIGNMTNV 193
Tc_Tci_043407           NFASAGAGALIGTRAGFV---VGLQTQLRYFGDLEDHYRHNLGDTKARQLLSTAVYLFSCGANDYLSPVGNNT--------------------------- 182
Tc_Tci_088899           NFASGGAGALISSHAGF-----------------------NLGDAKSRKLLSTAVYLFSCGANDYQSP----------YYP--YTHEQYVDIVTGNMTNV 81
Tc_Tci_372263           NFASGGA--------------VDLQTQLRYFGDLVNHYRQNLGDAKSRQLLSDAVYLFSCGANDYQSP----------YYS--YTQEQYANIVIGNTTKV 137
Tc_Tci_439592           NFASTGSGALNDTDAGFV---VDLQTQLQHFGDLENRYKQNLGDTEAQKLLSNAVYLFSSGANDYITFSVSNLSI--YSRT--FTNRQYTDMVIGNLTKV 236
Tc_Tci_456723           NFASGGAGALVETHAGSV---VDLKTQLKYISNLKKHFRRNFGDLKAEQILSNAVYLFSFGGNDYLTPVGNNDS---VLYL--YTHEAYVGMVIGNLTNA 419
Tc_Tci_539163           ------AGALINSHAGF---VVDLQTQLRYFGNLVNHYRQNLGDTKSRQLPSDAVYLFGYGGNDYQIP----------YYP--YTQEQYVDIMIGNMTNV 214
Tc_Tci_547473           NFASGGAGALVESHDGFV---IDLKTQLQYFKDLEKQFRQNLGDAETNRLLSDAVYLFSCGGNDYLSPEGNNDS---VLYPYTYTHEEYVGLVIGNLTNV 258
Tc_Tci_568598           KLCFRRAGALIDSHAGFV---VDLQTQLRYFGDLVNHYRRNLGDAKSRQLLSDAVYLFSCGANDYQSP----------YYS--YTQEQYADIVIGNTTNV 89
Tc_Tci_603146           NFASGGAGALISSHAGFV---VDLQTQLRYFCDLVNYYRQNLGDAKSRKLLSAAVYLFSCGANDYQSP----------YYP--YTHEQYVDIVTGNMTNV 241

Aa_PWA41198.1           IKA-----IYEKGGREFGVLTAPLIGCWPGIRIRQPGNTCNTEIDELTRLHNQALAKRLEHLEKKLEGFM---------YAKFDISTAISN--------- 250
Aa_PWA43567.1           IKG-----IYAKGGRKFGFLTVPLSGCSPGVRIGQPGYICHKGIDDIARIHNQKLAITLEHLEKQLEGLM---------YAKCDISTAITN--------- 278
Aa_PWA78027.1           IKG-----IYDKGGRKFGFLNVALLGCFPAIRVGQPGNTCNKEMDDIVRLHNQKLSKKLQHLEKQLEGFM---------YANFDISTAMYN--------- 366
Aa_PWA78028.1           IKG-----IYRKGGRKFGFLTVPLIGCFPALRVGQPGNTCNKEMNDIVRLHNQKLARKLEHLEKQLEGFM---------YAKFDISTAITN--------- 711
Aa_PWA80855.1           IKG-----IYEKGGRKIAFATIPLLACWPSVRAGRVGNTCNEELDLISSLHNQELTKKLQELENHLDGFM---------YSKFDLANEVNK--------- 278
Aa_PWA80856.1           IKG-----IYEKGGRKIAFATIPLLACWPSVRAGRVGNTCNEELDLISSLHNQELTKKLQELENHLDGFM---------YSKFDLANEVNK--------- 278
Cs_Cse_sc002489.1_g040.1IKG-----IYAKGGRKFGFLTVPLSGCSPGVRIGQPGYICHKGIDDIARIHNQKLAIKLDHLRKQLEGFM---------YSKFDISAAIIH--------- 158
Cs_Cse_sc002548.1_g010.1IKG-----IYEKGGRKFGIAAVPFLACWPSVRAGRVGNTCNEELDLISSLHNQELTKKLQELENHLDGFM---------YSKFDLANEVNK--------- 278
Cs_Cse_sc021205.1_g020.1IK-----GIYEKGGRKFGILTAPLIGCWPGIRMRQPGNTCNTEIDELTRLHNQALAKRLEHLEKQLEGFMY---------AKFDISTAISN--------- 640
Cs_Cse_sc055278.1_g010.1IKVATYRGIYEKGGRKFGVLTAPLIGCWPGIRIRQPGNTCNTEIDELTRLHNQALAKRLRTFG---ETYLC----------------------------- 596
Ha_XP_021984571.1       IKG-----IYEVGGRRFGFLNVPPLGCWPAIRLRQPGNICSKEIDDVIRLHNQALARKLENLETHMEGFM---------LSKLDFYTSMSN--------- 276
Tc_TcGLIP               IKG-----IYEKGGRKFGVVNVPLIGCWPGMRAKQPGNTCNTEVDELTRLHNQAFAKRLEQLEKQLEGFV---------YAKFDLSTAILN--------- 270
Tc_Tci_043407           --G-----IYDKGGRKFGFLNVALLGCFQAIRVGQPGNTCNKEMDDIV----------------------------------------------------
Tc_Tci_088899           IKG-----IYEKSGRKFGVVTAPLIGCWPGIRIRQSRNTCNTEIDELTRLHNQALAKRLEHLEKPLEGFM---------YARFDISTAISY--------- 158
Tc_Tci_372263           IKG-----IYEKGGRNFGVVTAPLIGCWPRMRVRQPGNTCNTEIDELTRLHNQAHAKRLEHLEKQLEGFMALSSLMVHLYHVNKPSIAISYCFLFTICQV 232
Tc_Tci_439592           IKG-----IHKKGGRKFGFLTVPPVGCFPATRSSQPGNTCNKEVDDIIRLHNRKLARKLQHLEKQLEGFM---------YSKFDISTAFTN--------- 313
Tc_Tci_456723           FKG-----IYKNGGRKFGILTVPPLACLPSIRAGRSGNTCNEELDILSSLHNQKLSKKLQELEKQLEGFK---------YAKFDLSTAVSN--------- 496
Tc_Tci_539163           IK-----EIYEKGGRKFEVVTAPLIGCWLGMRVRQPGNTCNTENNELTRLYNQALAKRLEHLEKQLEGFMY---------AKFDLSTAISN--------- 291
Tc_Tci_547473           FKG-----IYAKGGRKFGIITVPPLACWPSIRAERPGNTCSEELGVIVSLHNQKLSEKLQELENQLEGFI---------YAKFDQSTEINK--------- 335
Tc_Tci_568598           IK------VTTNGGIKFGVVTAPLIGCWPGMRVRQPGNTCNTEIDELTRLHNQALAKRLEHLEKQLEGFM---------YAKFDLSTAITN--------- 165
Tc_Tci_603146           IKG-----IYEKGRRKFRVVTAPLIGCWPGIRIRQSGNTCNTEINELTRLHNQALAKRLEHLEKPLEGFM---------YAKFDISTAISY--------- 318
```

```
Aa_PWA41198.1           ------------------------------RMKSPSKYGFKEGERACCGSGPFG----------------------------------------------  274
Aa_PWA43567.1           ------------------------------RMNNPSKYGFKDGETACCGSGPFR----------------------------------------------  302
Aa_PWA78027.1           ------------------------------RMNNPSKYGFKVGDTACCGSGPFK----------------------------------------------  390
Aa_PWA78028.1           ------------------------------RMNNPSKYGFNEGETACCGNGPLR----------------------------------------------  735
Aa_PWA80855.1           ------------------------------RMRNPSKYGFKVGDSACCGTGPFR----------------------------------------------  302
Aa_PWA80856.1           ------------------------------RMRNPSKY--------------------------------------------------------------
Cs_Cse_sc002489.1_g040.1------------------------------RMNNPSKYGFKDVETACCGSGPFR----------------------------------------------  160
Cs_Cse_sc002548.1_g010.1------------------------------RMTNPSKYGFKVGDSSCCGTGPFR----------------------------------------------  302
Cs_Cse_sc021205.1_g020.1------------------------------RMKNPSKYGFKEGERACCGSGPFRGIYSCGGMRGIKEFELCDNATEYL----------------------  688
Cs_Cse_sc055278.1_g010.1----------------------------------------------------------------------------------------------------
Ha_XP_021984571.1       ------------------------------RMKNPSQYGFKEGETACCGSGPFG----------------------------------------------  300
Tc_TcGLIP               ------------------------------RMKNPSKYGFKEGESACCGSGPFG----------------------------------------------  294
Tc_Tci_043407           ------------------------------------------GDTACCGSGPFR----------------------------------------------  235
Tc_Tci_088899           ------------------------------RMKNPSKY-----ERACCGSGPFG----------------------------------------------  177
Tc_Tci_372263           LYSCNFLSLCRAQEQYFLASLVLPVPRYPFKRQNVSFSSFVPHSSSPTKSSPYSFASVFFLPFFSRSNKVFSHQCVWCPRLSSSLCTLFAICRSLGNPQF  332
Tc_Tci_439592           ------------------------------RMNNPSKYGFKDGETACCGSGPFR----------------------------------------------  337
Tc_Tci_456723           ------------------------------RMNNPSKYGFKVGTSACCGSGPYR----------------------------------------------  520
Tc_Tci_539163           ------------------------------RMKNPSKYGFR-----------------------------------------------------------
Tc_Tci_547473           ------------------------------RMTNPSKYGFKVGDTACCGSGP------------------------------------------------  357
Tc_Tci_568598           ------------------------------RMKSHSIYGFKEGERACCGSGPFG----------------------------------------------  189
Tc_Tci_603146           ------------------------------RMKNPSKYGFKEAERACCGSGPFG----------------------------------------------  342

Aa_PWA41198.1           ------------------------------------------------GIYSCGGMRGIKEFELCEN------------ATEYLFFDCFHPNELASRQFA  314
Aa_PWA43567.1           ------------------------------------------------GKNSCGGKRGTTEYELCDN------------ATEFLFFDSAHPSEQASQQIA  342
Aa_PWA78027.1           ------------------------------------------------GIFSCGGKRGITDYELCDD------------VTEFFLFDSSHPTELAYRQFA  430
Aa_PWA78028.1           ------------------------------------------------GIYSCGGRRGITDYELCDN------------ATEFLFFDSIHPNEMADRQFA  775
Aa_PWA80855.1           ------------------------------------------------GIDSCGGKREVKEFELCDN------------ISDYLFFDSNHCTEVAIRQYA  342
Aa_PWA80856.1           ----------------------------------------------------------------------------------------------------
Cs_Cse_sc002489.1_g040.1------------------------------------------------GKNSCGGKRGTTEYELCDN------------ATEFLFFDSAHPNELANRQLS  164
Cs_Cse_sc002548.1_g010.1------------------------------------------------GIDSCGGKREVKEFELCDN------------ISDYLFFDSNHCTEVAIRQYA  342
Cs_Cse_sc021205.1_g020.1---------FFDCFHPNELASRQFAEMFWDGDSMVTQPYNLKALFEAMAVASSKSGALAAAVVVLCLSISSDCHENIHKQVLVVVLGDSLFDPGNNNYIN  779
Cs_Cse_sc055278.1_g010.1----------------------------------------------------------------------------------------------------
Ha_XP_021984571.1       ------------------------------------------------GVYSCGGMRRIKEYEVCDN------------VLEYLFFDSYHPNELASRQFA  340
Tc_TcGLIP               ------------------------------------------------GNYDCG---RIKEFGLCDN------------ATEYFFFDPFHPNELASRQFA  331
Tc_Tci_043407           ------------------------------------------------GIFSCGGKRGITEYELCDD------------VTEFFLFDSSHPTELAYRQFA  275
Tc_Tci_088899           ------------------------------------------------GIYSCGGMRRIKEFELCNN------------ATDYLFFDNFHPIELASHQFA  217
Tc_Tci_372263           FITFTCNVKWPEIKRYMAQYPELTRTDRADIMCKVFEQKVKDFVNFLKKVRTFGYVLQIEEYIYAEIPDHVEDPRGYKLVTDLMMHGPCGAANLGAPXIP  432
Tc_Tci_439592           ------------------------------------------------GNFSCGGKRGTMQYELCDN------------ASEYLFFDSLHPNELASSQFA  377
Tc_Tci_456723           ------------------------------------------------GIYSCGGKRGLQEYQLCDN------------INDYLFFDSYHPNEVANRQYA  560
Tc_Tci_539163           -----------------------------------------------------KAREHVAVVVLLEG--------------FIVVEGR------------  323
Tc_Tci_547473           ----------------------------------------------------------------------------------------------------
Tc_Tci_568598           ------------------------------------------------GIYSCGRMRGIKEFELCDT------------TTER---SKDTKGTLGTEESR  226
Tc_Tci_603146           ------------------------------------------------GIYSCGGMRRIKEFELCDN------------ATDYLFFDSFHPNELASHQFA  382

Aa_PWA41198.1           EMFWD-GDSMVTQPYNMKSLFEG-----------KPSTNFLPNDEL---- 348
Aa_PWA43567.1           EMFWK-GDSKVTAPYNLQALFS---------------------------- 363
Aa_PWA78027.1           EMFWE-GDYMVTAPYNLQALFEAMTVASSNSGFIVIIVLCLSIPTGSQAH 479
Aa_PWA78028.1           ELFWK-GDPTVTGPYNLKALFDGKP------------------------- 799
Aa_PWA80855.1           ELFWD-GDSDVTTPYNLKAFLQGELTTTSRMQQL---------------- 375
Aa_PWA80856.1           ------GTSELRNPSNLV-------------------------------- 298
Cs_Cse_sc002489.1_g040.1EMFWK-GDSVVTGPYNLQALFKGTLFFPMLFMISFGKFAYLFIMIEPVDK 1697
Cs_Cse_sc002548.1_g010.1ELFWD-GDSDVTTPYNLKAFFQAELAGLPLIPTYLDPRNNEFTYGANFAS 391
Cs_Cse_sc021205.1_g020.1TTTSFQSNFTPYGESYFNPPTGRFSNGRLIPDFIAEYAGLPLISAYLEPE 829
Cs_Cse_sc055278.1_g010.1--------------------------------------------------
Ha_XP_021984571.1       EMFWN-GDSIVTEPNSLEALFDGAASTTFLVHPNNEL------------- 376
Tc_TcGLIP               EMFWD-GDSMVTQPYNLKALFEG-----------KPSTKYLPNDEL---- 365
Tc_Tci_043407           EMFWK-GDYMVTAPYNLQALFKNN-------------------------- 298
Tc_Tci_088899           ETF----------------------------------------------- 220
Tc_Tci_372263           NYIVNTAELQVYILYELEAILNGFEKSVTDFGLQAPPNHLLKDLENKLLM 482
Tc_Tci_439592           KMFWK-GNSVVTKPYNLKKLFEV--------------------------- 399
Tc_Tci_456723           QLFWD-ADPNVTTPYNLQTLFXMSSSSLQVLPDRPARMLGKQANGG---T 606
Tc_Tci_539163           ------------GE------------------------------------ 325
Tc_Tci_547473           --------------------------------------------------
Tc_Tci_568598           --------------------------------------------------
Tc_Tci_603146           ETF----------------------------------------------- 385
```

Supplemental Figure 7. Molecular phylogenetic analysis of *T. cinerariifolium* GDSL lipase (TcGLIP). TcGLIP-related proteins were searched by BLASTP against 14 different plant protein sets and incorporated into maximum likelihood tree analysis using ORTHOSCOPE method[56] (A). Proteins incorporated into this tree include 9 from *T. cinerariifolium*, 10 from *C. seticuspe*, 10 from *A. annua*, 10 from *H. annuus*, 9 from *Z. mays*, 10 from *V. vinifera*, 10 from *M. truncatula*, 10 from *S. lycopersicum*, 10 from *P. trichocarpa*, 10 from *O. sativa*, 8 from *G. raimondi* , 10 from *N. tabacum*, 10 from *G. max*, and 9 from *A. thaliana*. TcGLIP nodes are indicated in blue; *T. cineariifolium* nodes and edges are indicated in red; other Asteraceae nodes and edges are indicated in orange. The amino acid sequence alignments for maximum likelihood tree analysis are shown in panel B.

Supplemental Figure 8.

A

```
Tci_094327          ----MSFDFSRVGVVCSFSLIAER--------------------FPGVLGGSGGSGSISASTESSFKDTTKVTKFSVDFG   56
AaSPB (PWA57483.1)  MEWNTKWDWENLAMFGSKGVASPKKLQSTDWGFKEGQDIDGSSNHSGVLGGSGGSGSISASTESTFKDTTKVTKFSVDFG   80

Tci_094327          GGELNKISPSLQASVFSSDQSIGLKLSERTYCENNLPRSNSSVSTDK-------------------------KDYHRKHM   121
AaSPB (PWA57483.1)  GGELNKSSPSLEASVCSSDQSIGLKLGKRTYFENNLPRSNSSVSTVKKVKLSQQSAPIICQVMGCDRDLSSAKDYHRKHR   160

Tci_094327          VYDIHLKALKVIVVGLK----------HRLDEFRRWARYCYSRIFCGDNEK----------------------LKRRFIV   169
AaSPB (PWA57483.1)  VCDVHSKSLKVIVAGLERRFCQQCSRFHGLPEFDGKKRSCRKRLADHNARRRKPHQETIQFNSRSLSSSYYDGPRQLSFA   240

Tci_094327          FAGSVIVYNERRLQELGN---------------------------------------RLKMNNIAFDKGNSHVVYVPFSS   210
AaSPB (PWA57483.1)  FNNGPMVQTKPAVSSMWENTCNSKHSVATVKAEKYGGHDVLTHSQGVQFPNPVSMTPTLSFNRNISSKGTTAGVFDQGSS   320

Tci_094327          SIYKAILSLPFFPHIGQAVRRLAARGTIFTRNGD--------------------------------------QGSTKRNG   252
AaSPB (PWA57483.1)  LELRRALSLLSNNSWGSSESDFGCLDHPMHTNGPNIAQQGMHSAPHGLPLLSPEYWQIDHQSVDPHIHGTHFQGSQILKA   400

Tci_094327          CYML-------------     256
AaSPB (PWA57483.1)  PYGIQYSNPMDEIVQPL     417
```

B

```
TcGLIP(AFJ04755.1)  -MAVASRKLGALVLVAVLCLSLPTGCLS-SQQAAALFIFGDSVFDPGNNNHINTHVNFKANFWPYGQSYFSSPTGRFSDG   78
Tci_043407          MANTSSESRRFAAVIIITCLSIPTNCHANIHKQVLVFVFGDSLFDPGNNNYINTTAAFQANFTPYGESYFYPPTGRFSNG   80
Tci_043410          ----------------------------------------------------------MANFWPYGESYFTSPTGRFSNG   22

TcGLIP(AFJ04755.1)  RIIPDFIAEYASLP-IIPAYLEP-NNDFTHGANFASAGAGALIASHAGLAVGLQTQLRYFGDLVDHYRQNLGDIKSRQLL   156
Tci_043407          RLMPDFIAEYAGLPPLIPAYLEPGNNEFTYGANFASAGAGALIGTRAGFVVGLQTQLRYFGDLEDHYRHNLGDTKARQLL   160
Tci_043410          RLIPDFIAEYARLP-LIPPYLEPGNNEFMYGANFASAGAGVLIDTKAGFVVDLQTQLRYLGDVENKYRENLGDTGARQLL   101

TcGLIP(AFJ04755.1)  SDAVYLFSCGGNDYQSPYYP---YTQEQYVDIVIGNMTNVIKGIYEKGGRKFGVVNVPLIGCWPGMRAKQPGNTCNTEVD   233
Tci_043407          STAVYLFSCGANDYLSPVG--------------------NNTGIYDKGGRKFGFLNVALLGCFQAIRVGQPGNTCNKEMD   220
Tci_043410          SSAVYLFSCGINDYVSFVGSNVSNTDEQYTDMVIGSLTTVIKGIYAKGGRKFGFVTVPLLGCSPGFRISQPGNICNKRID   181

TcGLIP(AFJ04755.1)  ELTRLHNQAFAKRLEQLEKQLEGFVYAKFDLSTAILNRMKNPSKYGFKEGESACCGSGP-------FGGNYDCG---RIK   303
Tci_043407          DIV----------------------------------------------GDTACCGSGP-------FRGIFSCGGKRGIT   247
Tci_043410          DIARLHNRKFAMKLEHLQKQLDGFMYAKFDISAAIINRMNNPSEYANRLVDSLQRCFGKGIPSKLGFLVLFVFCLWVPTD   261

TcGLIP(AFJ04755.1)  EFGLCDNATEYFFFDPFHPN-------------ELASRQFAEMFWDGDSMVTQPYNLKALFEGKPSTKYLPNDEL-----   365
Tci_043407          EYELCDDVTEFFLFDSSHPT-------------ELAYRQFAEMFWKGDYMVTAPYNLQALFKNN----------------   298
Tci_043410          SWGNIDKRVALFVFGDSLFDPGNNNYINTTSYQANFWPYGESYFNPPTGRFSNGRLIPDFIAEYARLPLIPPYLKPGNK   341

TcGLIP(AFJ04755.1)  --
Tci_043407          --
Tci_043410          EF 343
```

Supplemental Figure 8. Amino acid sequence alignment of Tci_094327 and *A. annua* squamosa promoter-binding (SPB) transcription factor (A), Tci_043407, Tci_043410, and TcGLIP (B).
